# Updating Health Canada’s Heat-Health Messages for the Environment and Climate Change Canada Heat Warning System: A Collaboration with Canadian Experts

**DOI:** 10.3390/ijerph22081266

**Published:** 2025-08-13

**Authors:** Emily J. Tetzlaff, Melissa MacDonald, Glen P. Kenny, Brittany Murphy, Rachel F. Siblock, Ahmed Al-Hertani, Rebecca C. Stranberg, Peter Berry, Melissa Gorman

**Affiliations:** 1 Climate Change and Health Office, Health Canada, Government of Canada, Ottawa, ON K1A 0K9, Canadarachel.siblock@hc-sc.gc.ca (R.F.S.); ahmed.al-hertani@hc-sc.gc.ca (A.A.-H.);; 2 Human and Environmental Physiology Research Unit, University of Ottawa, Ottawa, ON K1N 6N5, Canada; 3 Health and Air Quality Forecast Services, Meteorological Service of Canada, Environment and Climate Change Canada, Government of Canada, Gatineau, QC J8X 4C6, Canada; 4 Clinical Epidemiology Program, Ottawa Hospital Research Institute, Ottawa, ON K1Y 4E9, Canada

**Keywords:** heat event, heat wave, health communication, heat alert response system, public health

## Abstract

It is critical to inform the public of the threat heat poses to health and provide actionable guidance on mitigating this risk before, during, and after heat events. To help educate the public during heat events, Health Canada works closely with Environment and Climate Change Canada (ECCC) to distribute heat-health messaging through a weather warning system. However, the warning system’s current list of messages dates back over a decade. Continually evaluating and updating messages is critical to ensure they are based on the best evidence available. A review was conducted to assess the existing heat-health messages and propose new messages based on recent empirical studies. The proposed messages were reviewed to ensure that readability and equity considerations were integrated. Academic, public health and meteorology experts across Canada reviewed the proposed messages and applied further revisions. The original list of heat-health messages included 12 messages. Based on the evidence and external reviews provided by 42 experts (academic: *n* = 9; public health: *n* = 22; meteorology: *n* = 11), messages were removed, merged, added and revised. The final list used by ECCC includes 30 messages. Health Canada’s heat-health messages disseminated through ECCC’s weather warning system were revised to ensure they are important, action-oriented, evidence-based, readable, equitable, regionally applicable, and timely. Ensuring these messages reflect current knowledge will be an ongoing and iterative process to support the public’s preparedness efforts to protect themselves and others during heat events.

## 1. Introduction

Extreme heat significantly impacts morbidity and mortality and affects health systems in Canada [1,2]. For this reason, governments at all levels, in partnership with community-level organizations, have generated targeted strategies and measures to reduce heat-related vulnerability [3]. Most common among these strategies are heat alert and response systems (HARS) that trigger community preparedness response plans and strategic communication efforts [3]. Critically, HARS communication plans seek to inform health professionals, community organizations and the Canadian public of the significant threat heat poses to health and to provide timely, actionable guidance to help people make informed decisions and modify their behaviours before, during and after heat events [3,4,5].

One avenue in which this heat-health messaging is shared is through the Environment and Climate Change Canada (ECCC) weather warning system. The public receives these messages alongside special weather statements and heat warnings [3] that are issued up to one day before potential heat events on the WeatherRadio, Canada.ca/weather, and the WeatherCAN app. However, this provides the public with minimal lead time and limits their ability to proactively prepare for a heat event or take protective measures during one (e.g., checking if their air conditioning is functioning before the heat event has commenced or rescheduling non-essential activities to limit exposure to the heat). Further, heat can have a lagged effect and continue to pose a risk even after the heat event concludes (as indicated when meteorological threshold criteria are no longer met) because temperatures may remain elevated indoors [6]. The British Columbia Coroners Service detailed this phenomenon in a post-event report, which indicated that heat-related deaths continued for over two weeks after the historic 2021 Western Heat Dome began [7]. Thus, releasing proactive guidance via early warnings and including action messages when heat events are declared over can better position the public to prevent adverse heat-related health outcomes or act if a health effect is experienced. Although some public health authorities provide pre-heat season messaging through other avenues (i.e., webpages) [8], the weather warning system does not currently do so.

The current list of heat-health messages in the warning system was developed in 2011 through collaboration among Health Canada, ECCC, and regional health agencies [3]. Since the development of these messages, various experimental and observational studies have been published on the efficacy of common heat-mitigation strategies. Reviewing the literature for each message to ensure it is based on the best available scientific evidence is essential for enhancing community protection from adverse heat-related health outcomes. Furthermore, when reviewing the risks and consequences of a message’s inclusion and its potential for positive or adverse impacts, consideration should be given to the presence of scientific evidence, as well as any scientific uncertainty or disagreement. The importance of continual evaluation is a critical component of HARS, as it increases the effectiveness of health adaptation measures [3].

The effects of heat events are disproportionately experienced by those at risk due to demographics and health conditions (e.g., older adults aged ≥65 years), those at risk due to socioeconomics and living conditions (e.g., people who are experiencing homelessness), and those at risk due to work or recreational activities (e.g., people working inside without air conditioning) [9]. Additionally, individuals facing communication challenges, literacy or language barriers are disproportionately affected [10,11,12]. With heat-health communication initiatives considered a critical public health protection strategy, understanding the additional challenges groups may experience in accessing or interpreting the information is vital. Furthermore, personal, home-based heat-mitigation strategies are often geared toward those with the financial means to implement them (e.g., air conditioning is costly for households with lower incomes) [13]. Thus, there is a need to consider alternative adaptive strategies that could be used by those who are socially or materially marginalized [14]. Therefore, heat-health messages developed with an equity lens are essential, given the increase in heat event occurrence and intensity, combined with a growing and diverse population in Canada. To ensure that all members of the public can access and interpret information related to heat-health protection, factors such as readability and equity (e.g., age, gender, race, disability) must be considered during message development and refinement.

Lastly, Canada spans a large geographic area with unique climatology between and within provinces and territories; thus, there are regional differences in how heat is experienced and impacts health. For example, individuals living in rural settings experience and respond to heat differently from those in densely populated cities due to factors such as the urban heat island effect. In addition, people residing in northern communities may have fewer opportunities for seasonal acclimatization [6]. Thus, it is essential that regional applicability be considered; however, this is not reflected in the current heat-health messaging.

With Canada experiencing a rising frequency of extremely hot days and constant changes in the drivers of health vulnerability at the individual and community level [15], heat-health messaging can become outdated if not regularly reviewed. This paper aims to review and refine Health Canada’s current list of heat-health messages disseminated through ECCC’s weather warnings system to ensure they are important, action-oriented, evidence-based, and reflect considerations such as readability, equity, and regional applicability. Furthermore, this work proposes additional messages to support proactive protection before heat events and the continued reduction in health risks after heat events are declared over.

## 2. Materials and Methods

This study received ethical approval from the Privacy Management Division (HC-PR-2024-000008, 21 June 2024) and the Health Canada Research Ethics Board (REB 2024-008H, 9 July 2024). The methods followed align with the Strengthening the Reporting of Observational Studies in Epidemiology (STROBE) guidelines [16] (Supplementary Material File S1).

### 2.1. Developing the Revised Messaging

The existing list of Health Canada’s heat-health messages disseminated through ECCC’s weather warning system included 12 messages. To support the determination of whether each message should be retained or removed, a peer-reviewed [17] literature search was conducted in MEDLINE (Ovid), Embase (Ovid), CINAHL (EBSCOhost), and Global Health (EBSCOhost) on 23 November 2022. The literature review aimed to gather evidence on the effectiveness of the interventions mentioned within the heat-health messages employed in the ECCC warning system. Therefore, the primary search concepts comprised terms related to heat exposure, such as heat illness, heat stroke, dehydration, and cooling interventions listed in the existing messages, including air conditioning, fans, curtains, blinds, shade, water immersion, and clothing. No limits to language or publication date were applied. Search results were exported to Covidence—a web-based tool for conducting reviews (Melbourne, Australia)—and duplicates were eliminated using the platform’s duplicate identification feature. The title and abstract of the remaining records underwent a screening protocol based on the Cochrane Rapid Review Method [18]. The complete search strategy is presented in Supplementary Material File S2.

**Figure 1:**
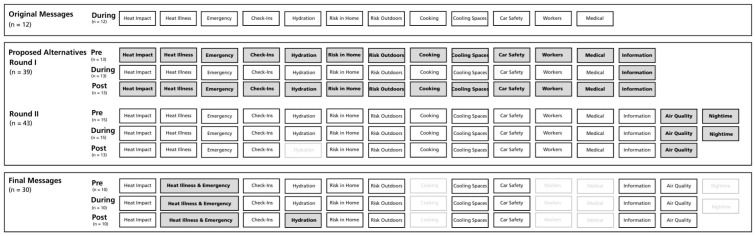
Visual depiction of the review and revision process for heat-health messages in Environment and Climate Change Canada’s weather warning system. The process began with 12 original messages. From the literature review, 1 new message (Information) was proposed, and each message was duplicated and adapted for before, during and after heat events. This produced 13 messages per timepoint and 39 total. These messages were sent for expert review, after which 1 new message (Air Quality) was added to all 3 timepoints, 1 new message (Nighttime) was added to the before and during timepoints, and 1 message (Hydration) was removed from the after timepoint. This brought the totals to 15 messages for the before and during timepoints, 13 for after, and 43 overall. A second expert review then led to merging 2 messages (Heat Illness and Emergency), removing 4 messages (Cooking, Workers, Medical and Nighttime) from all timepoints, and restoring 1 message (Hydration) to the latter timepoint. This final set included 10 messages per timepoint and 30 total. Note: Grey-filled cells with bold text indicate messages added or merged, and light grey semi-transparent cells indicate messages that were removed.

The literature review results identified several opportunities to revise the existing messages, developed initially in 2011, based on new evidence. First, modifications were made to expand the list of heat-vulnerable populations to include individuals susceptible based on exposure, sensitivity and adaptive capacity. Second, revisions were made to more clearly distinguish the severity of the heat-related illnesses listed and to ensure that all primary signs and symptoms were included. Third, additions were also made related to features of performing wellness check-ins (i.e., timing, frequency) that were omitted from the original version but identified as necessary in the literature. Fourth, alternative strategies that can support cooling (i.e., air conditioning, fan use, relocating to a cooler space) and methods to reduce heat production (i.e., avoiding use of heat-generating appliances) were added. Other public spaces to seek reprieve from the heat were included in the messaging based on the empirical evidence available in the literature. Furthermore, the statement on workers at risk was revised to provide additional guidance based on current occupational health and safety literature. Lastly, it was also identified that the existing messaging primarily addressed actions to be taken during a heat event but offered little guidance on preparations before an event and did not address health effects that occur past the conclusion of a heat event. Therefore, the statements were triplicated and revised to be tailored for release before the heat event (issued before the event), during a heat event, and after the heat event (issued when warning criteria are no longer met). This resulted in 39 messages (Figure 1). The complete reporting of the message modifications from the original to the subsequent version is documented in Supplementary Material File S3 (English) and File S4 (French).

### 2.2. Developing the Assessment Instrument

To facilitate the review of the modified messages by a group of external experts, an electronic assessment form was developed based on critical elements of consideration for risk communication, including importance, action-orientation, evidence base, readability, equity, and regional applicability [19,20,21,22,23,24,25]. Each component included two to three questions. Additionally, the instrument was designed to provide an opportunity for reviewers to reflect on a series of prompts related to the presentation/organization and comprehensiveness of the messages.

Once developed, the project leads consulted with a Public Engagement Advisor from the Communications and Public Affairs Branch (Health Canada/Public Health Agency of Canada, Ottawa, Canada) to develop the online tool on Qualtrics (Qualtrics XM Platform^®^). During this phase of development, three internal pilots were conducted with team members (E.J.T., M.G., R.F.S.) (*n* = 6) to ensure the assessment reflected the key elements of consideration and to verify the online tool’s functionality. Once finalized in English, the tool was professionally translated (Lionbridge Technologies, LLC) and then reverse-translated by a bilingual colleague to ensure accuracy (see Acknowledgements). A copy of the finalized assessment instrument for Round I is provided in Supplementary Material File S5, available in both English and French. A second iteration of the assessment instrument was developed for Round II and is provided in Supplementary Material File S6, available in both English and French.

### 2.3. Conducting External Expert Review: Round I and II

A list of researchers and public health experts from across Canada was developed by scanning recent publications, reviewing Canadian research institutes and public health authorities, and reviewing existing contact lists (*n* = 38). The list reflected expertise in a range of knowledge areas, including human physiology, communications, climate change, population and public health, and disaster and emergency preparedness. Each identified contact (*n* = 41) received a letter of invitation to participate and was given three weeks, with one reminder, to provide written consent to participate and complete their online assessment. Feedback was received from 26 experts (English: *n* = 20, French: *n* = 6) (Supplementary Material File S7). The results were reviewed, and modifications were applied, which resulted in 43 messages (see Section 3: Results) (Figure 1).

The revised messaging was then recirculated to the external experts for a second round of review. The external experts for Round II (*n* = 64) included those who participated in Round I, as well as additional contacts recommended by the participating experts. The reviewers were given two weeks, with one reminder, to provide final comments and feedback on the messaging for Health Canada’s consideration. Feedback was received from 31 experts (English: *n* = 24, French: *n* = 7) (Supplementary Material File S7).

Final modifications were applied after the internal project team reviewed the feedback (see Section 3: Results). Modifications included combining messages due to comments regarding length, redundancy, and how an ECCC meteorologist would determine which to include. Some messages were also removed based on low priority ranking, equity-based concerns, the need to include essential caveats to support the advice that would exceed the word count limits of the ECCC warning system and based on the opinion that some messages were more appropriate for other dissemination methods focused on education and awareness rather than for a public heat warning. This stage of review resulted in 30 messages (Figure 1).

### 2.4. Data Analysis

#### 2.4.1. Descriptive Statistics

Data analysis was conducted at two points: first, after Round I and then again following Round II of the expert review. The number of respondents and percentage of the total sample were calculated for numerical data based on individual response rates to each question. The number and percentage of respondents selecting each response option were reported for multi-select questions. For each value, the mean, standard deviation (SD), median (x͂) and range (R) were reported, as well as the number of respondents to the individual question (*n*). All descriptive findings are reported in Supplementary Material File S7.

#### 2.4.2. Text-Based Analysis

For open-ended responses, quotations were taken verbatim from the online submission form. French responses were translated into English. For all quotes, basic spelling and grammar modifications were performed where necessary for readability. The written responses were then thematically collated by message and by the critical elements of consideration (important, action-oriented, evidence-based, readable, equitable, applicable), and the internal project team analyzed the content. Based on the feedback, various modifications were applied to the messaging (see Section 3: Results).

#### 2.4.3. Readability Assessment

During each iteration of the messages, a readability assessment was conducted to assess whether the proposed messages were written in plain language to support the public in understanding and taking action to mitigate health risks [26,27]. The readability of each message was calculated using the Simplified Measure of Gobbledygook (SMOG) [28], Flesch Reading Ease (FRE), Flesch-Kincaid Grade Level (FKGL) [29], Automated Readability Index (ARI) [30], and Gunning Fog Index (GFI) [31]. These assessment tools are used to estimate the complexity of the text and calculate the number of formal years of schooling needed to comprehend written material, thus representing the literacy sensitivity of the webpage or resource [32]. These measures have all been previously used to assess the difficulty of vocabulary and concept needs of health education documents [33,34,35,36,37]. However, as there is currently no gold standard or universal agreement on which tool is best suited for specific materials, a selection of indexes considering both characters, words and sentences, and polysyllabic words (i.e., words with multiple syllables) was used [38]. To apply these formulas, each message was analyzed using the online tool WebFX (https://www.webfx.com/tools/read-able/#enter-text-tab, accessed on 23 November 2022), which provided us with a score for each readability scale and text statistics (words and complex words). The resulting readability scores were then compared against the recommended reading grade level for public health materials, indicating that the language level should be appropriate to the age and comprehension/reading ability of approximately a grade six [39,40,41,42,43,44].

### 2.5. Final Revisions and Application in the Weather Warning System

The internal project team then worked with ECCC to finalize the messages for the weather warning system. This process required independent senior management review and approvals within ECCC and Health Canada, as well as modifications due to limitations of the software system. One primary consideration raised during this process was ensuring that the most important messages would be shared with the public, as the current method required system end-users (i.e., meteorologists) to typically select two to three messages from the complete list to include. Additionally, there were restrictions on the word count of the messages and limits on the number of embedded hyperlinks used to direct individuals to Health Canada’s extreme heat webpage and online resources. To address these concerns, the revised messages were made more succinct, the number of hyperlinks was reduced, and messages were further grouped based on their requirement for inclusion within each heat warning and the specifics of the heat event (i.e., Message 15: Air Quality). No changes were made to the content or intent of the messages at this stage. The final 30 messages are presented in Supplementary Material File S3 (English) and File S4 (French).

## 3. Results

### 3.1. Expert Reviewer Descriptors

Overall, responses were received from 42 experts. Fifteen experts participated in both rounds; the remaining experts participated in only one round (Round I: *n* = 11, Round II: *n* = 16). Responses were received in English (79%, *n* = 33) and French (21%, *n* = 9). All provinces and territories in Canada were represented, with the majority of responses coming from Ontario (29%, *n* = 12) and Quebec (19%, *n* = 8), followed by British Columbia (12%, *n* = 5) and Newfoundland and Labrador (12%, *n* = 5). The remaining provinces and territories had fewer than three respondents; some respondents represented more than one region. Respondents represented 24 different public health or environmental groups/organizations, including provincial and territorial (19%, *n* = 19) and federal health (3%, *n* = 3) and meteorological authorities (11%, *n* = 11), as well as academic/research institutions (21%, *n* = 9). The respondents reported an average of 14 ± 12 years (median: 13, range: 2–24) of experience in environmental health, epidemiology, public health, health promotion, physiology, climate change, disaster and emergency management, and/or risk communications. All respondents had a college diploma or higher, with the majority having a post-graduate degree specializing in their discipline (Master’s: 29%, *n* = 12; Doctorate: 36%, *n* = 15).

### 3.2. Ranking Agreement and Inclusion

Based on the ranking exercise, the messages were reordered from most important to least important (Table 1). In addition to ranking, each participant was asked to reflect on whether they agreed with the modifications and whether the message(s) should be included within the ECCC warning system (Table 1). Most experts agreed with the changes applied for each message, with an average agreement of 82 ± 8% (median: 83%, range: 64–93%). However, messages 2 (heat illness) and 3 (emergencies) had slightly more participants who disagreed with the modifications (17% and 17%, respectively). Further, message 15 on air quality achieved only 64% agreement, with 10 participants identifying they were unsure (18%) or disagreed with the modifications (18%). Most experts also felt the proposed messages should be included in the ECCC warning system, with an average agreement of 81 ± 10% (median: 83%, range: 59–97%). However, messages 12–14 received more unsure and disagreement scores (17%, 17%, and 28%, respectively) (Table 1). The ranking results and feedback related to agreement with modifications and inclusion within the final set of messages were used to help reorder the messages (Supplementary Material Files S3 and S4).

### 3.3. Alignment with the Critical Elements of Risk Communication

In response to the reflective prompts related to the importance, action-orientation, evidence base, readability, equity, and regional applicability, the experts typically felt these considerations were reflected in all (‘yes’) or most (‘some’) of the proposed messages (Table 2). The following subsections present the responses to each point of consideration for risk communication with quotations from participants embedded to provide examples and further context. The results presented below were used to help revise the messages (Supplementary Material Files S3 and S4).

#### 3.3.1. Important

Many experts felt that the proposed messages are important to include in the ECCC weather warning system and are important at the time points indicated (preheat event, during, post-event) (Table 2). For example, one expert said: *“Important! We are hearing clearly from our [interest holders] that preheat event messaging is vital to help them be prepared for the events.”* However, others felt the proposed messages lacked value at the indicated time points. In particular, a few comments related to adjusting the timing of the messaging or the importance of specific messages being released both before and during a heat event. There were a few comments related to some of the messaging also being appropriate for the preheat season, instead of or in addition to being delivered before a heat event. For example, *“I feel that some preheat event messages would be more valuable preheat season and before an event. With only 48 h before the heat warning, some suggested activities and information may not be implemented and may require more time for the public/user to implement.”* Similarly, some respondents indicated that the post-event messaging should better align with the other time points or questioned the necessity of the post-event messaging. A few respondents also raised the importance of ensuring the messages have credibility.

#### 3.3.2. Action-Oriented

Most experts felt the messages were sufficiently action-oriented and would motivate behavioural change, and many thought the actionability improved between iterations (Table 2). Some respondents suggested further methods to modify the messages to strengthen motivation for action. Other participants highlighted specific examples of additional actions or steps that should be integrated into the proposed messages (e.g., *“It would be helpful to have specific actions rather than ‘check-on,’ such as ‘call’ or ‘visit’”*). Many experts also commented on the reframing of messages to begin with the action, for example: *“Possible alternative wording that begin with the action verb. Learn about (OR Know, become familiar with…) your rights, employer responsibilities, and workplace’s policies concerning excessive heat.”* Further, many experts felt that the messages were appropriate to action at the indicated time. However, participants also suggested emphasizing the urgency of action within some messages. Some respondents also commented on the need for additional direction on ‘who’ messages should be directed at to ensure action is taken. Many experts also recommended embedding hyperlinks within the messages to provide readers with additional awareness information tools to facilitate action. For example: *“When warning messages get too long, there is a tendency not to read the entire message. The recommendations listed here can be incorporated into a link where additional information/resources are provided.”*

#### 3.3.3. Evidence-Based

When asked about the evidence base for the messaging, most experts felt the messages were supported (Table 2)—the most discussed topic related to evidence on fan use. Comments in Round I emphasized the need for greater specificity on fan use; however, Round II debated the effective threshold. For example: *“Mostly, it is action-oriented, but the note for older adults about fans is vague…What are people supposed to do then? If this is too difficult to nuance, is there a point at which fan use becomes harmful—to help qualify the message.”* Some experts did feel that a few of the proposed messages were missing necessary conditional disclaimers (e.g., limitations for specific at-risk populations). Some typical comments related to the need to be more direct and concrete with the messages based on evidence, specifically for hydration, indoor temperature thresholds, car safety, risk period and event duration. One public health expert also raised the question regarding the inclusion of the recommendation for a cool shower, stating: *“I wonder about ‘cool shower’ and how this will be interpreted. Could result in peripheral vasoconstriction and not promote heat loss?”*

#### 3.3.4. Readability

When asked whether the messages were written at a reading grade level appropriate for the public and free of jargon or complex terms, most experts indicated that they were appropriate (Table 2). However, some commented that they felt the reading level exceeded the recommended level of grade 6 [39,40,41,42,43,44]. In doing so, some participants highlighted specific examples of terms that may be challenging for members of the public and proposed substitutions or definitions to enhance clarity. Some participants also highlighted opportunities to improve consistency in language and information between the timing of the messages. Some francophone reviewers suggested opportunities to improve the translation by removing phrases or terms that do not translate well. Lastly, a few participants commented on the overall sentence structure, typically related to message length, sentence phrasing, prepositional phrases, and syntax inconsistencies.

Further, and in alignment with some expert responses, the readability analysis demonstrated that each message’s average reading grade level was typically grade 7, slightly above the recommended reading level (grade 6). The readability results for each iteration of the messages are available in Supplementary Material File S7. On average, the finalized messages had 27 ± 12 words (median: 25, range: 8–73) and 3 ± 2 complex words (median: 3, range: 0–11). The FRE scale reported an average reading level of grade 8–9 (65 ± 15%, median: 66%, range: 25–94%) and found that 3 (9%) messages achieved the ideal reading level (grade 6). The FKGL indicated an average reading level of grade 7 ± 3 (median: 6, range: 2–19), with 16 (48%) of the messages achieving the recommended grade 6 reading level. The GFI indicated an average reading level of grade 9 ± 3 (median: 8, range: 3–19), and 7 (21%) of the messages achieved the recommended score. The SMOG indicated an average reading level of grade 7 ± 2 (median: 6, range: 2–14), and 19 (58%) of the messages achieved the optimal reading score (grade 6). Lastly, the ARI indicated an average reading level of grade 7 ± 4 (median: 6, range: 0–22) but found that 19 (58%) of the messages achieved the recommended reading level.

#### 3.3.5. Equity

Many experts indicated that the messages were equitable and that the proposed heat-protective measures were feasible for individuals of various socio-economic backgrounds (Table 2). However, some participants raised specific points of concern that *“not [all messages are] necessarily appropriate for those in all socio-economic conditions.”* For example, a few respondents highlighted that some messages assume people have a home, a car, a phone or access to cooling resources. In Round I, some experts noted that the terms ‘home’ and ‘house’ were frequently used and may not be inclusive for those facing homelessness or housing precarity. Similarly, some experts highlighted cases where *“the wording used was exclusive,”* with most examples related explicitly to references of family or friends, for example: *“Could use more inclusive language to consider those who do not have social networks or are unhoused.”* The experts raised concerns that, with the knowledge that poverty is one of the most significant risk factors for heat-related injury and death, the messages could be more focused on the most at-risk populations.

**Figure 2:**
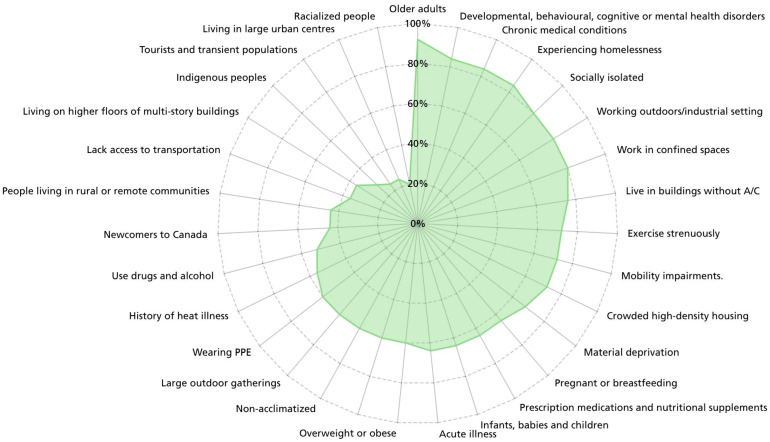
Visual depiction of the respondent feedback on which heat-vulnerable populations should be included in the heat-health messages shared through the Environment and Climate Change Canada weather warning system. Note: PPE, personal protective equipment; A/C, air conditioning.

Respondents also commented on the list of proposed heat-vulnerable populations (Figure 2), often indicating that other specific at-risk groups could or should be included (Supplementary Material File S7). For example, some participants raised the need to include people with mobility restrictions, Indigenous communities, those with cognitive or neurodevelopmental challenges, those who cannot drive themselves to a cooling centre, pregnant people or those who engage in the use of non-prescription substances.

#### 3.3.6. Regional Applicability

Most experts agreed that the messages are regionally applicable and appropriately reflect various climate conditions in Canada (Table 2). However, a few comments raised concerns that some regional differences were not reflected in the messaging, which could result in certain populations not seeing themselves portrayed in the messaging. For example, in message 3 (Heat Emergency), it was raised that *“911 doesn’t exist in all of Canada…, so I would ask that it say, ‘call the emergency health care provider or go directly to your nearest health centre.’”* It was also noted that a greater focus on messaging specific to indoor environments is needed, especially considering that not all regions have prevalent use of air conditioning. Some respondents also commented on the need to address cascading and compounding crises, such as air quality issues and wildfires. For example, during Round I of the review, many experts called for including messages specifically addressing heat and special air quality messages or air quality advisements. The following quote illustrates this request: *“Heat and wildfire smoke is unfortunately common in our summer months, with often overlapping events. Focused messaging with these events in mind would be well received. Additional consideration of heat events during active emergency evacuations would be appropriate, even if a link to appropriate provincial sites is provided for more information during these events.”* A few participants also commented on the differences between urban and rural regions that must be considered; for example, not all rural communities have cooling centres, the inclusion of terms like shopping centres may be urbanized, and many remote areas have limited forecasting and cooling infrastructure.

### 3.4. Finalized Messages

Based on the feedback, 30 messages were retained, including 10 messages for release with early warnings and advisories, 10 messages for release during heat events, and 10 additional messages for when an event is declared over (Table 3). The complete reporting of all message variations and revisions is documented in Supplementary Material File S3 (English) and File S4 (French). The during heat event messages will become effective in the summer of 2025. Furthermore, the ECCC meteorologists will endeavour to incorporate the pre- and post-event messages into their social media posts and communicate with provincial, territorial, and regional partners where possible, until the warning system program is further reconfigured to release these messages. 

## 4. Discussion

### 4.1. Important, Action-Oriented and Evidence-Based

Clear and concise messaging that communicates only the most critical health-protective advice is needed during heat events to support the public in reducing their risk of adverse health outcomes. Therefore, a foundational principle of this project was to ensure that the list of messages for the ECCC weather warning system reflects the most important messages. By using the expert reviewers’ ranking exercise (Table 1) and overall feedback (Supplementary Material File S7), the list of messages was simplified, and messages that were best suited for other dissemination platforms were removed. The remaining messages were then positioned in order of priority (Table 3). Further, through this process, it was ensured that the messages reflected the most important contexts. For example, experts reiterated that during heat events, the most significant risk is within indoor environments (e.g., people’s living spaces or homes). This is supported by the literature [6] and reviews of recent heat events such as the 2021 Western Heat Dome (i.e., 98% of deaths occurred indoors) [7]. Within the original messages, there was no direct reference to indoor overheating, but there were references to outdoor environments. Therefore, this review process enabled a greater emphasis on indoor temperatures.

This study also sought to ensure the messages included were based on the latest scientific evidence. As a result of the evidence review and input from senior scientists and public health officials, some of the original messages were removed because they were not evidence-based or were deemed to provide a lower level of impact for heat-health protection (e.g., *“When it’s hot, eat cool, light meals”* was removed) (Table 1 and Table 2, Figure 1). However, the expert review process revealed some disagreement around certain heat-mitigating behaviours. For example, the expert review’s findings highlighted that conditions related to fan use caused disagreement and raised questions regarding the degree of specificity required for other messages (e.g., how much water a person should drink?; how long they should spend in a cooling centre?) (Supplementary Material File S7). Messaging developed for ECCC’s weather warning system must be generalized for a broad public audience and adhere to platform constraints (e.g., word count). As a result, providing detailed guidance for specific populations, settings, or temperature thresholds was not feasible within the message format itself. To address this, hyperlinks were included in the messages that direct the public to Health Canada’s extreme heat webpage, where more detailed and context-specific heat-health information is available in various formats. As new evidence emerges, the heat-health messages disseminated by ECCC’s weather warning system and Health Canada’s extreme heat webpage will continue to be revised.

In event-based risk messaging, effective risk reduction requires that messages raise awareness and improve knowledge, which supports behaviour change [45]. A common finding from expert reviewer feedback was that earlier versions of the messages lacked emphasis on the actual behaviour an individual should be taking to mitigate risk (Table 2). As a result, numerous messages were revised to begin with a verb (e.g., watch, check, drink, close, plan, move, monitor) to make the messages action-oriented (Supplementary Material File S7). Beyond the written structure of the messages, expert participants also posed questions about the proposed cascade of actions. For example, one iteration of message 9 (Cooling Spaces) included, *“Check your thermostat or thermometer. Move to a cool public space…if your living space is hot”*; however, the message did not define the temperature of what would be considered ‘hot’ and requires the initiation of the move to a cooler space. Therefore, future work and subsequent revisions to the ECCC weather warning messages will be needed to enhance the specificity of the proposed heat-mitigating actions.

### 4.2. Readability, Equity and Regional Applicability

The usefulness and effectiveness of health promotion materials for public education and personal decision-making largely depend on the use of language that is accessible to people with varying levels of health literacy [46]. Therefore, a readability analysis was conducted to ensure that the heat-health messages addressed barriers that individuals with literacy challenges, newcomers to Canada [11], and non-official language speakers [11,12] may face when interpreting the presented information. By combining feedback from the experts (Supplementary Material File S7) and best practices [47], the overall reading grade level of the original messages was slightly reduced (e.g., SMOG: 7.5 > 7.3; ARI: 9 > 8.5; complex words: 11.5% > 9.7%). The reading level became more closely aligned with the recommended sixth-grade reading level promoted by health authorities [39,40,41,42,43,44]. However, some of our messages still exceed the recommended reading level, particularly those related to signs and symptoms and first aid response, due to the inclusion of complex polysyllabic medical terms (e.g., exhaustion, dizziness, fatigue, loss of consciousness). Other studies have noted the challenge of using medical terminology in public health materials [47]. In the absence of being able to eliminate these terms, various hyperlinks were embedded within the messages to enhance reader engagement and allow users to access additional information hosted on the Government of Canada’s extreme heat webpage.

Another priority of this review process was to ensure equity was considered. Experts consulted raised the importance of ensuring that messages did not discriminate based on ableism or financial capacity (Supplementary Material File S7). To address these concerns, changes were implemented to provide heat-mitigating strategies for individuals with reduced mobility and low income, such as those unable to purchase or operate fans or air conditioning units. The terms ‘home’, ‘house’ and ‘basement’ were also replaced with more equitable ones like ‘living space’ and ‘cooler area’ to recognize various living situations. Acknowledging that not all feel welcome in specific indoor public spaces due to real and perceived stigma (e.g., people experiencing homelessness [48] and people who use drugs [49,50]), shaded parks were also added to the list of cool public spaces. Further, the experts were presented with over 30 different at-risk groups to capture opinions on which priority populations should be included. In most cases, opinions strongly aligned (e.g., older adults; 98% yes, 2% unsure), but in other cases, perspectives diverged on the inclusion of certain groups (e.g., newcomers to Canada and those with language barriers; 44% yes, 48% unsure; 8% no) (Figure 2). As it was critical to balance enhancing specificity while reflecting generality, decisions were made to include only a few populations that showed the greatest agreement among experts and alignment with available empirical evidence. These population groups were included in both messages 1 (Heat Impact) and 3 (Check-Ins). As this decision limited the number of groups listed, hyperlinks were also added to direct the public to additional guidance that includes more detailed considerations for other at-risk groups [9] (Table 3).

Lastly, it was ensured that the heat-health messages developed were as generalizable as possible across Canada’s diverse regions, given that ECCC’s weather warning system disseminates alerts and notifications nationally. Expert reviewers from across the country offered critical insight into different climate challenges experienced in the Northern Territories compared to urban centres, the limited access to specific resources such as cooling centres in rural settings, and differences in availability of emergency response services (i.e., 9-1-1 is not available in some northern territories) (Supplementary Material File S7). While acknowledging that these messages cannot capture every provincial, territorial and regional need, changes were applied to enhance their applicability where possible. For example, terms like ‘malls’ were replaced with terms that also apply in more rural areas, like community centres and libraries, and the reference to calling 9-1-1 was extended to include “or your emergency health provider.”

### 4.3. Strengths and Limitations

While a pan-national perspective was sought to collect a representative dataset, most expert respondents were from Ontario, Quebec, and the provincial and territorial level (Supplementary Material File S7). Researchers looking to further this work may consider conducting similar inquiries, specifically targeting expert reviewers from regional authorities and local-level communities. This local-level representation would be particularly important in the northern territories because the warming rate and heat responses are likely to vary considerably compared to the provinces, which are more strongly represented in this current dataset. Further, although reminders were sent in both rounds of review, the response rate for repeat participation was low (i.e., only 15 of 42 experts participated in both rounds) (Table 2). Future work may consider alternative methods (e.g., interviews, focus groups) to increase the response rate and facilitate additional comparative analysis.

Next, due to the project’s focus, some expert reviewers’ specific suggestions could not be applied. For example, there were comments requesting that the messages be available in various languages and graphic forms (Supplementary Material File S7); however, the ECCC weather warning system only releases text-based messages in English and French. Additionally, many comments wanted the messages to target specific populations (Figure 2), which was not feasible given that the ECCC weather warning system needs to apply to the broader public and has word count limitations. However, this feedback has been captured to inform Health Canada’s future efforts to develop health promotion materials for specific groups at greater risk of negative heat impacts.

Next, although this review and revision process was well-supported by the literature and reviewed by experts, it did not integrate how the public perceives and interacts with these messages, which represents a limitation of the study. The presented study does not include empirical testing with end-users to assess public comprehension and behavioural impact when receiving these messages. Future revisions to the messages would benefit from engagement of non-subject matter experts and the broader public through public validation and evaluation to measure message effectiveness. Such investigations could include focus testing to assess whether the public is receiving and accessing the messages, how they interpret them and whether the messages lead to protective behaviour change during heat events. Further, a knowledge gap remains regarding how messaging is received through the electronic heat warning system, since this is the ECCC’s primary mechanism for message dissemination. Understanding public engagement with these messages could help improve the messages so they are actionable and accessible to the populations they intend to serve.

Lastly, this is an evolving research area, so additional studies may have been published since the initial literature review was completed. Therefore, the evidence base for the heat-health messages must be considered a snapshot of knowledge at the time of the literature search. Future studies could continue to expand on this work and seek to synthesize and evaluate the growing body of observational and experimental evidence on the effectiveness of personal heat-alleviation strategies, while also exploring how equity factors influence access to and use of these heat mitigation measures. This could be achieved through systematic reviews and meta-analyses to provide the most up-to-date account of empirical evidence and facilitate timely updating as new information becomes available. To support this iterative process, Health Canada, in collaboration with ECCC, will continue to lead the maintenance of the messages, as emerging evidence warrants.

## 5. Conclusions

Our external consultation with experts in environmental health, epidemiology, public health, health promotion, physiology, climate change, disaster and emergency management, and risk communications from across Canada helped inform modifications to the heat-health protective messages disseminated through the ECCC weather warning system. Through engagement with provincial, territorial and federal health and meteorological authorities and academics, this study sought to ensure the messages were important, action-oriented, and founded in evidence. Further, by integrating the expert responses, revisions were made to enhance readability and ensure equity and regional applicability. This work also proposed a tiered system based on the timing of messaging to provide proactive protection before heat events and sustained vigilance after heat events are declared over. During heat events, these messages help raise awareness and support the public in taking health-protective and potentially life-saving actions. Although subsequent investigations will be necessary to continue this work as new evidence emerges, our findings represent a crucial step toward enhancing heat preparedness and communication efforts targeting the Canadian public. Furthermore, the transparent reporting of this systematic and iterative process may also help inform the next steps for revising other public heat preparedness strategies and HARS in Canada and globally, ensuring they align with the current and future needs of the public.

## Figures and Tables

**Table 1:** Summary of expert opinions on ranking, agreement and inclusion.

	Ranking	Agreement with Modifications			Inclusion in the ECCC Warning System		
**Messages**	**Avg ± SD** **Median** **Range**	**Yes** ***n* (%)**	**Unsure** ***n* (%)**	**No** ***n* (%)**	**Yes** ***n* (%)**	**Unsure** ***n* (%)**	**No** ***n* (%)**
Message 1 Heat Impact	5th ± 5 2nd 1st–15th	24 (83%)	1 (3%)	4 (14%)	25 (86%)	3 (10%)	1 (3%)
Message 2 Heat Illness	3rd ± 2 2nd 1st–11th	23 (79%)	1 (3%)	5 (17%)	26 (90%)	3 (10%)	0 (0%)
Message 3 Emergency	5th ± 4 4th 1st–15th	22 (76%)	2 (7%)	5 (17%)	23 (79%)	4 (14%)	2 (7%)
Message 4 Check-Ins	4th ± 2 4th 1st–9th	23 (79%)	3 (10%)	3 (10%)	26 (90%)	2 (7%)	1 (3%)
Message 5 Hydration	6th ± 3 5th 1st–13th	23 (79%)	3 (10%)	3 (10%)	25 (86%)	4 (14%)	0 (0%)
Message 6 Risk in Home	8th ± 3 7th 3rd–14th	22 (76%)	4 (14%)	3 (10%)	20 (69%)	7 (24%)	2 (7%)
Message 7 Risk Outdoors	7th ± 3 7th 2nd–14th	26 (90%)	1 (3%)	2 (7%)	26 (90%)	1 (3%)	2 (7%)
Message 10 Car Safety	7th ± 4 7th 2nd–15th	26 (90%)	1 (3%)	2 (7%)	24 (83%)	3 (10%)	2 (7%)
Message 9 Cooling Spaces	10th ± 3 9th 4th–15th	24 (83%)	1 (3%)	4 (14%)	24 (80%)	3 (10%)	3 (10%)
Message 15 Nighttime	10th ± 2 10th 6th–13th	21 (75%)	5 (18%)	2 (7%)	24 (83%)	3 (10%)	2 (7%)
Message 13 Information	9th ± 5 10th 1st–15th	24 (83%)	3 (10%)	2 (7%)	28 (97%)	1 (3%)	0 (0%)
Message 11 Workers	10th ± 3 10.5th 3rd–15th	25 (86%)	0 (0%)	4 (14%)	21 (72%)	3 (10%)	5 (17%)
Message 14 Air Quality	11th ± 3 11th 3rd–15th	18 (64%)	5 (18%)	5 (18%)	21 (72%)	5 (17%)	3 (10%)
Message 12 Medical	11th ± 3 11.5th 5th–15th	27 (93%)	1 (3%)	1 (3%)	21 (72%)	3 (10%)	5 (17%)
Message 8 Cooking	13th ± 3 13th 4th–15th	26 (90%)	0 (0%)	3 (10%)	17 (59%)	4 (14%)	8 (28%)

Note: The messages are listed in order of the ranking exercise completed by the expert reviewers. *n*, count; ECCC, Environment and Climate Change Canada; Avg, average; SD, standard deviation.

**Table 2:** Summary of expert reviewer’s responses on importance, action-orientation, evidence base, readability, equity, and regional applicability.

		Yes *n* (%)	Some *n* (%)	No *n* (%)	Change in Response *n* (%)
**Importance**	**Are the proposed messages important for the ECCC weather warning system?**				
	Review Round I Review Round II	20 (74%) 17 (59%)	7 (26%) 12 (41%)	0 (0%) 0 (0%)	≡11 (74%) +2 (13%) -2 (13%)
	**Are the proposed messages important at the time points indicated (preheat event, during, post-event)?**				
	Review Round I Review Round II	15 (56%) 24 (83%)	10 (37%) 5 (17%)	1 (4%) 0 (0%)	≡6 (40%) +7 (47%) -2 (13%)
**Action-Orientation**	**Are the proposed messages sufficiently action-oriented?**				
	Review Round I Review Round II	15 (56%) 21 (72%)	12 (44%) 8 (28%)	0 (0%) 0 (0%)	≡11 (74%) +4 (27%) -0 (0%)
	**Are the proposed messages appropriate to action at the time points indicated?**				
	Review Round I Review Round II	15 (56%) 25 (86%)	11 (41%) 4 (14%)	1 (4%) 0 (0%)	≡8 (53%) +6 (40%) -1 (7%)
**Evidence Base**	**Are the proposed messages evidence-based?**				
	Review Round I Review Round II	19 (73%) 18 (62%)	5 (19%) 11 (38%)	1 (4%) 0 (0%)	≡12 (80%) +1 (7%) -2 (13%)
	**Where applicable, do the proposed messages include the necessary conditional disclaimers needed?**				
	Review Round I Review Round II	13 (48%) 17 (61%)	13 (48%) 10 (36%)	0 (0%) 1 (4%)	≡12 (80%) +1 (7%) -2 (13%)
**Readability**	**Are the proposed messages written at a reading grade level appropriate for the public?**				
	Review Round I Review Round II	18 (67%) 20 (69%)	9 (33%) 7 (24%)	0 (%) 2 (7%)	≡9 (60%) +4 (27%) -2 (13%)
	**Are the proposed messages free of jargon or complex terms?**				
	Review Round I Review Round II	16 (59%) 24 (83%)	11 (41%) 4 (14%)	0 (0%) 1 (3%)	≡6 (40%) +8 (53%) -1 (7%)
**Equity**	**Are the proposed messages equitable?**				
	Review Round I Review Round II	17 (63%) 16 (55%)	8 (30%) 13 (45%)	2 (7%) 0 (0%)	≡10 (67%) +2 (13%) -3 (20%)
	**Do the proposed messages provide heat-protective measures that are feasible for individuals of various socio-economic backgrounds?**				
	Review Round I Review Round II	16 (59%) 18 (62%)	9 (33%) 10 (34%)	2 (7%) 1 (3%)	≡9 (60%) +4 (27%) -2 (13%)
**Applicability**	**Are the proposed messages applicable to your geographic region?**				
	Review Round I Review Round II	23 (85%) 21 (75%)	4 (15%) 7 (25%)	0 (0%) 0 (0%)	≡11 (74%) +2 (13%) -2 (13%)
	**Are the proposed messages appropriately reflective of various climate conditions in Canada?**				
	Review Round I Review Round II	18 (67%) 19 (70%)	8 (30%) 8 (30%)	1 (4%) 0 (0%)	≡12 (80%) +3 (20%) -0 (0%)

Note: The final column reflects the change in response for participants that engaged in both Round I and Round II of the review process (*n* = 15); (≡) represents responses that did not change between rounds (e.g., yes to yes; same to same; no to no), (+) represents responses that scored higher in Round II compared to Round I (e.g., no to same; same to yes), and (-) represents scores that lowered from Round I to II (e.g., yes to same; same to no). ECCC, Environment and Climate Change Canada; *n*, count.

**Table 3:** Comparison of original messages to final revised messages for the Environment and Climate Change Canada weather warning system.

Original Message		Final Message
**Message 1:** Extreme heat affects everyone. The risks are greater for young children, pregnant women, older adults, people with chronic illnesses and people working or exercising outdoors.	✓	**Pre:** Extreme heat can affect everyone’s health. Determine if you or others around you are at greater risk of heat illness.
	✓	**During:** Take action to protect yourself and others—extreme heat can affect everyone’s health. Determine if you or others around you are at greater risk of heat illness such as older adults, people with chronic disease, and those who are socially isolated.
	✓	**Post:** Take precautions to reduce your risk of heat illness, as it may develop after the heat event is over. Continue to check yourself and others for signs.
**Message 2:** Watch for the symptoms of heat illness: dizziness/fainting; nausea/vomiting; rapid breathing and heartbeat; extreme thirst; decreased urination with unusually dark urine.	🗶	Merged.
**Message 3:** Heat stroke is a medical emergency! Call 911 or your local emergency number immediately if you are caring for someone, such as a neighbour, who has a high body temperature and is either unconscious, confused or has stopped sweating. While waiting for help—cool the person right away by: moving them to a cool place, if you can; applying cold water to large areas of the skin or clothing; and fanning the person as much as possible.	✓	**Pre:** Be aware of the early signs of heat exhaustion in yourself and others it can rapidly become a life-threatening emergency like heat stroke. Heat can cause dehydration and heat exhaustion, including swelling, rash, cramps, fainting, and worsening of pre-existing health conditions. Heat stroke is a medical emergency.
	✓	**During:** Watch for the early signs of heat exhaustion in yourself and others. Signs may include headache, nausea, dizziness, thirst, dark urine and intense fatigue. Stop your activity and drink water. Heat stroke is a medical emergency! Call 9-1-1 or your emergency health provider if you, or someone around you, is showing signs of heat stroke which can include red and hot skin, dizziness, nausea, confusion and change in consciousness. While you wait for medical attention, try to cool the person by moving them to a cool place, removing extra clothing, applying cold water or ice packs around the body.
	✓	**Post:** Continue to monitor yourself and others for signs of heat exhaustion and heat stroke. The effects of heat can continue to be experienced even after an extreme heat event is over.
**Message 4:** Check on older family, friends and neighbours. Make sure they are cool and drinking water.	✓	**Pre:** Talk to family, friends and neighbours to see how they are preparing for the heat. Create a plan to support each other and check in multiple times a day with those at greater risk.
	✓	**During:** Check on older adults, those living alone and other at-risk people in-person or on the phone multiple times a day.
	✓	**Post:** Check in on older adults, those living alone and other at-risk people in-person or on the phone, for a few days after the heat event ends, as it can remain hot indoors.
**Message 5:** Drink plenty of cool liquids, especially water, before you feel thirsty to decrease your risk of dehydration. Thirst is not a good indicator of dehydration.	✓	**Pre:** Drink water often to avoid dehydration. Dehydration can lead to a heat illness.
	✓	**During:** Drink water often and before you feel thirsty to replace fluids. Exposure to heat will cause your body to lose fluids through sweat.
	✓	**Post:** Continue to drink water to stay hydrated as the heat may remain high.
**Message 6:** Keep your house cool. Block the sun by closing curtains or blinds.	✓	**Pre:** Find ways to keep your living space cool and make sure air-conditioning, fans, and windows are working.
	✓	**During:** Close blinds, or shades and open windows if outside is cooler than inside. Turn on air conditioning, use a fan, or move to a cooler area of your living space.
	✓	**Post:** The temperature of your living space can remain high even after a heat event is over. Continue to stay cool by opening windows and using a fan to move cool air indoors.
**Message 7:** Avoid sun exposure. Shade yourself by wearing a wide-brimmed, breathable hat or using an umbrella.	✓	**Pre:** Plan and schedule outdoor activities during the coolest parts of the day or reschedule them until the heat event has passed. If outdoors, seek shaded areas.
	✓	**During:** Plan and schedule outdoor activities during the coolest parts of the day. Limit direct exposure to the sun and heat. Wear lightweight, light-coloured, loose-fitting clothing and a wide-brimmed hat.
	✓	**Post:** Be careful outdoors as it remains hot.
**Message 8:** When it’s hot eat cool, light meals.	🗶	Removed.
**Message 9:** Seek a cool place such as a tree-shaded area, swimming pool, shower or bath, or air-conditioned spot like a public building.	✓	**Pre:** Find air-conditioned or cool spots in your area where you can go such as community centre, library, stores or shaded park. Plan for help with transport if needed.
	✓	**During:** Move to a cool public space such as a cooling centre, community centre, library or shaded park if your living space is hot.
	✓	**Post:** Monitor your living space and keep cool as needed. It can still be hot inside even after a heat event is over.
**Message 10:** Never leave people or pets inside a parked vehicle.	✓	**Pre:** Never leave people, especially children, or pets inside a parked vehicle. Check the vehicle before locking it to make sure no one is left behind.
	✓	**During:** Never leave people, especially children, or pets inside a parked vehicle. Check the vehicle before locking to make sure no one is left behind.
	✓	**Post:** Never leave people, especially children, or pets inside a parked vehicle. Check the vehicle before locking it to make sure no one is left behind.
**Message 11:** Outdoor workers should take regularly scheduled breaks in a cool place.	🗶	Removed.
**Message 12:** Ask a health professional how medications or health conditions can affect your risk in the heat.	🗶	Removed.
**Message 13:** N/A	✓	**Pre:** Be aware of advice and resources from local and public health authorities that can help you stay cool and safe from the heat.
	✓	**During:** Check heat alerts via the Public Weather Alerts website or the WeatherCAN app. Follow the advice of your region’s public health authority.
	✓	**Post:** Keep helpful contacts and heat-health web links to be prepared for the next heat event.
**Message 14:** N/A	✓	**Pre:** When there is an extreme heat event occurring with wildfire smoke, prioritize keeping cool.
	✓	**During:** When there is an extreme heat event occurring with wildfire smoke, prioritize keeping cool.
	✓	**Post:** If air quality has improved, open windows and doors to move cool air into the space at night, if safe.
**Message 15:** N/A	🗶	Removed.

Note: Underlining represents terms that are hyperlinked in the final messages used in the Environment and Climate Change Canada weather warning system. N/A, not applicable; Pre, pre-heat event; During, during heat event; Post, post-heat event.

## Data Availability

The original contributions presented in this study are included in the article/Supplementary Materials. Further inquiries can be directed to the corresponding author.

## Supplementary Materials

The following supporting information can be downloaded at https://www.mdpi.com/article/10.3390/ijerph22081266/s1, Supplementary Material File S1—STROBE Checklist; Supplementary Material File S2—Search Strategy; Supplementary Material File S3—Statement Iterations English; Supplementary Material File S4—Statement Iterations French; Supplementary Material File S5—Qualtrics Round 1; Supplementary Material File S6—Qualtrics Round 2; Supplementary Material File S7—Descriptive Results.

## Abbreviations

The following abbreviations are used in this manuscript:

ECCC	Environment and Climate Change Canada
HARS	Heat Alert and Response System(s)
SMOG	Simplified Measure of Gobbledygook
FRE	Flesch Reading Ease
FKGL	Flesch–Kincaid Grade Level
ARI	Automated Readability Index
GFI	Gunning Fog Index
