# Supplementary material for: Updating Health Canada’s Heat-Health Messages for the Environment and Climate Change Canada Heat Warning System: A Collaboration with Canadian Experts
Source: Int J Environ Res Public Health. 2025 Aug 13;22(8):1266. doi: 10.3390/ijerph22081266 (PMC12386431; doi:10.3390/ijerph22081266)
Supplement: Supplementary file 1 [file ijerph-22-01266-s001.zip › IJERPH_Supplementary Material File S2_Search Strategy.pdf]

Updating Health Canada’s Heat-Health Messages for the Environment and Climate Change Canada Heat Warning System: A Collaboration with Canadian Experts

Supplement B

Search Methods

A peer reviewed (1) search strategy was conducted on November 23, 2022 in MEDLINE (Ovid), Embase (Ovid), CINAHL (EBSCOhost), and Global Health (EBSCOhost) (see supplemental files for full search details). No limits to language or publication date were applied. The main search concepts comprised of terms related to residential housing, heat exposure, and cooling interventions. Search results were exported to Covidence (Melbourne, Australia) and duplicates were eliminated using the platform’s duplicate identification feature.

Acknowledgements

We thank Evan Sterling (P.Eng, MLIS) and Nicholas Dehler (MI) from the University of Ottawa Library for peer review of the MEDLINE search strategy.

Bibliography

McGowan J, Sampson M, Salzwedel DM, Cogo E, Foerster V, Lefebvre C. PRESS Peer Review of Electronic Search Strategies: 2015 guideline statement. Journal of Clinical Epidemiology. 2016;75:40–6.

Table 1. MEDLINE (Ovid) Search Strategy
Ovid MEDLINE(R) ALL: 1946 to November 22, 2022

#	Searches	Results
1	exp housing/	36224
2	exp residential facilities/	57512
3	exp home environment/	299
4	independent living/	10512
5	(house* or housing* or home? or facility or facilities or residential* or residence? or apartment* or condo* or accommodation* or dwelling* or domicile* or building* or lodging* or shelter? or indoor* or in-door*).ti,ab,kf.	999870
6	((space* or room* or setting*) adj3 (personal or confined or living)).ti,ab,kf.	7767
7	(within door* or withindoor*).ti,ab,kf.	0
8	(independent* adj2 (live? or living)).ti,ab,kf.	7363
9	or/1-8	1041855
10	hot temperature/	123503
11	extreme heat/	527
12	extreme hot weather/	21
13	global warming/	4181
14	exp heat stress disorders/	6779
15	((hot* or warm*) adj4 (temperature* or condition? or environment* or weather* or indoor* or spell? or climate*)).ti,ab,kf.	28331
16	((extreme* or intens* or high* or summer* or severe*) adj2 (heat* or warm* or hot* or temperature*)).ti,ab,kf.	112364
17	(heatwave* or heat-wave*).ti,ab,kf.	3073
18	(global warming or greenhouse effect*).ti,ab,kf.	12002
19	(climate* adj2 chang*).ti,ab,kf.	57558
20	((heat or thermal) adj3 (stress* or ill* or sick* or fatigu* or exhaust* or strain* or exert* or cramp* or collaps* or syncop* or prostrat* or stroke* or expos* or shock* or disorder* or injur* or diseas* or trauma*)).ti,ab,kf.	93695
21	(heatstroke* or hypertherm* or overheat* or over-heat*).ti,ab,kf.	42388
22	or/10-21	396561
23	air conditioning/	2857
24	cryotherapy/	5644
25	ventilation/ and (cool* or cold*).ti,ab,kf.	253
26	(baths/ or immersion/) and (cool* or cold* or ice* or icy or cryo*).ti,ab,kf.	1310
27	(air adj1 (condition* or revitali* or central)).ti,ab,kf.	4675
28	(climate* adj1 control*).ti,ab,kf.	704
29	(fan or fans).ti,ab,kf.	7356
30	((evaporative or air or desert) adj2 (cooler* or cooling)).ti,ab,kf.	1415
31	(swamp adj2 (cooler* or box*)).ti,ab,kf.	5
32	((glaz* or film* or tint* or window*) adj3 (photochromic or thermochromic)).ti,ab,kf.	181
33	(window* adj4 (tint* or block* or alumin?um or blind? or curtain* or film? or cover*)).ti,ab,kf.	887
34	((close* or closing or shut* or draw* or pull* or open*) adj4 (curtain* or blind? or window* or shade*)).ti,ab,kf.	7017
35	((heat pump* or ductless) adj6 (cool* or cold*)).ti,ab,kf.	31

36	((cool* or cold* or phase change or ice* or icy or cryo*) adj4 (vest? or garment* or attire* or cloth* or apparel or gear or scarf or scarves or towel* or personal* or wear* or therap* or pack? or compress* or spray* or mist*)).ti,ab,kf.	7842
37	((wet* or damp*) adj3 (cloth* or spong*)).ti,ab,kf.	254
38	((cool* or cold* or ice* or icy) adj3 (immers* or bath* or shower*)).ti,ab,kf.	2982
39	((ice? or icy or cold* or cool*) adj4 (drink* or beverage* or hydrat* or fluid* or pack?)).ti,ab,kf.	3312
40	((drink* or ingest*) adj2 water?).ti,ab,kf.	61382
41	((light* or loose* or air* or less*) adj4 (cloth* or dress* or apparel* or attire* or garment*)).ti,ab,kf.	1285
42	or/23-41	103469
43	9 and 22 and 42	924
44	(exp animals/ or exp animal experimentation/ or exp models, animal/) not (humans/ or exp human experimentation/ or exp persons/ or human*.ti.)	5047541
45	(animal* or ape or apes or chimpanzee* or gerbil* or guineapig* or guinea pig* or hamster? or hare or hares or macaque* or mammal* or mice or monkey* or mouse or primate* or rabbit* or rat or rats or rodent? or calf or calves or bovine* or cow*).ti.	2068712
46	43 not (44 or 45)	737

Table 2. Embase (Ovid) Search Strategy

Embase Classic+Embase: 1947 to 2022 November 22

#	Searches	Results
1	housing/	32318
2	residential home/	8142
3	home environment/	6520
4	home/	9403
5	building/	8795
6	independent living/	6791
7	(house* or housing* or home? or facility or facilities or residential* or residence? or apartment* or condo* or accommodation* or dwelling* or domicile* or building* or lodging* or shelter? or indoor* or in-door*).ti,ab,kf.	1334302
8	((space* or room* or setting*) adj3 (personal or confined or living)).ti,ab,kf.	9387
9	(within door* or withindoor*).ti,ab,kf.	3
10	(independent* adj2 (live? or living)).ti,ab,kf.	10224
11	or/1-10	1362025
12	high temperature/	36399
13	critical thermal limit/	12
14	critical thermal maximum/	126
15	exp heat wave/	1175
16	heat/	100367
17	greenhouse effect/	16664
18	exp heat stress/	20943
19	exp heat injury/	9245
20	hyperthermia/	25478
21	heat intolerance/	910
22	heat intolerance/	910
23	((hot* or warm*) adj4 (temperature* or condition? or environment* or weather* or indoor* or spell? or climate*)).ti,ab,kf.	30858

24	((extreme* or intens* or high* or summer* or severe*) adj2 (heat* or warm* or hot* or temperature*)).ti,ab,kf.	110410
25	(heatwave* or heat-wave*).ti,ab,kf.	3258
26	(global warming or greenhouse effect*).ti,ab,kf.	12986
27	(climate* adj2 chang*).ti,ab,kf.	56268
28	((heat or thermal) adj3 (stress* or ill* or sick* or fatigu* or exhaust* or strain* or exert* or cramp* or collaps* or syncop* or prostrat* or stroke* or expos* or shock* or disorder* or injur* or diseas* or trauma*)).ti,ab,kf.	109157
29	(heatstroke* or hypertherm* or overheat* or over-heat*).ti,ab,kf.	54196
30	or/12-29	437568
31	cooling/	28298
32	cooling system/	665
33	exp cold compress/	910
34	air conditioning/	32180
35	cryotherapy/	21223
36	cooling water/	1055
37	ventilation/ and (cool* or cold*).ti,ab,kf.	1219
38	(air adj1 (condition* or revitali* or central)).ti,ab,kf.	6387
39	(climate* adj1 control*).ti,ab,kf.	743
40	(fan or fans).ti,ab,kf.	9994
41	((evaporative or air or desert) adj2 (cooler* or cooling)).ti,ab,kf.	1650
42	(swamp adj2 (cooler* or box*)).ti,ab,kf.	8
43	((glaz* or film* or tint* or window*) adj3 (photochromic or thermochromic)).ti,ab,kf.	117
44	(window* adj4 (tint* or block* or alumin?um or blind? or curtain* or film? or cover*)).ti,ab,kf.	1088
45	((close* or closing or shut* or draw* or pull* or open*) adj4 (curtain* or blind? or window* or shade*)).ti,ab,kf.	9366
46	((heat pump* or ductless) adj6 (cool* or cold*)).ti,ab,kf.	23
47	((cool* or cold* or phase change or ice* or icy or cryo*) adj4 (vest? or garment* or attire* or cloth* or apparel or gear or scarf or scarves or towel* or personal* or wear* or therap* or pack? or compress* or spray* or mist*)).ti,ab,kf.	11808
48	((wet* or damp*) adj3 (cloth* or spong*)).ti,ab,kf.	356
49	((cool* or cold* or ice* or icy) adj3 (immers* or bath* or shower*)).ti,ab,kf.	4168
50	((ice? or icy or cold* or cool*) adj4 (drink* or beverage* or hydrat* or fluid* or pack?)).ti,ab,kf.	4593
51	((drink* or ingest*) adj2 water?).ti,ab,kf.	83605
52	((light* or loose* or air* or less*) adj4 (cloth* or dress* or apparel* or attire* or garment*)).ti,ab,kf.	1861
53	or/31-52	206277
54	11 and 30 and 53	1565
55	(animals/ or exp animal experiment/ or exp animal model/) not (humans/ or exp human experiment/ or exp named groups of persons/ or human*.ti.)	4035690
56	(animal* or ape or apes or chimpanzee* or gerbil* or guineapig* or guinea pig* or hamster? or hare or hares or macaque* or mammal* or mice or monkey* or mouse or primate* or rabbit* or rat or rats or rodent? or calf or calves or bovine* or cow*).ti.	2532417
57	54 not (55 or 56)	1387

Table 3. CINAHL (EBSCOhost) Search Strategy

#	Searches	Results
S41	S10 AND S21 AND S40	151
S40	S22 OR S23 OR S24 OR S25 OR S26 OR S27 OR S28 OR S29 OR S30 OR S31 OR S32 OR S33 OR S34 OR S35 OR S36 OR S37 OR S38 OR S39	17,012
S39	TI ((light* or loose* or air* or less*) N4 (cloth* or dress* or apparel* or attire* or garment*)) OR AB ((light* or loose* or air* or less*) N4 (cloth* or dress* or apparel* or attire* or garment*))	533
S38	TI ((drink* or ingest*) N2 water#) OR AB ((drink* or ingest*) N2 water#)	6,137
S37	TI ((ice# or icy or cold* or cool*) N4 (drink* or beverage* or hydrat* or fluid* or pack#)) OR AB ((ice# or icy or cold* or cool*) N4 (drink* or beverage* or hydrat* or fluid* or pack#))	974
S36	TI ((cool* or cold* or ice* or icy) N3 (immers* or bath* or shower*)) OR AB ((cool* or cold* or ice* or icy) N3 (immers* or bath* or shower*))	702
S35	TI ((wet* or damp*) N3 (cloth* or spong*)) OR AB ((wet* or damp*) N3 (cloth* or spong*))	66
S34	TI ((cool* or cold* or phase change or ice* or icy or cryo*) N4 (vest# or garment* or attire* or cloth* or apparel or gear or scarf or scarves or towel* or personal* or wear* or therap* or pack# or compress* or spray* or mist*)) OR AB ((cool* or cold* or phase change or ice* or icy or cryo*) N4 (vest# or garment* or attire* or cloth* or apparel or gear or scarf or scarves or towel* or personal* or wear* or therap* or pack# or compress* or spray* or mist*))	2,715
S33	TI ((heat pump* or ductless) N6 (cool* or cold*)) OR AB ((heat pump* or ductless) N6 (cool* or cold*))	5
S32	TI ((close* or closing or shut* or draw* or pull* or open*) N4 (curtain* or blind# or window* or shade*)) OR AB ((close* or closing or shut* or draw* or pull* or open*) N4 (curtain* or blind# or window* or shade*))	1,388
S31	TI (window* N4 (tint* or block* or alumin#um or blind# or curtain* or film# or cover*)) OR AB (window* N4 (tint* or block* or alumin#um or blind# or curtain* or film# or cover*))	134
S30	TI ((glaz* or film* or tint* or window*) N3 (photochromic or thermochromic)) OR AB ((glaz* or film* or tint* or window*) N3 (photochromic or thermochromic))	3
S29	TI (swamp N2 (cooler* or box*)) OR AB (swamp N2 (cooler* or box*))	1
S28	TI ((evaporative or air or desert) N2 (cooler* or cooling)) OR AB ((evaporative or air or desert) N2 (cooler* or cooling))	154
S27	TI (fan or fans) OR AB (fan or fans)	1,493
S26	TI (climate* N1 control*) OR AB (climate* N1 control*)	121
S25	TI (air N1 (condition* or revitali* or central)) OR AB (air N1 (condition* or revitali* or central))	709
S24	(MH "Ventilation") AND (TI (cool* or cold*) OR AB (cool* or cold*))	43
S23	(MH "Air Conditioning")	424
S22	(MH "Cryotherapy")	3,217
S21	S11 OR S12 OR S13 OR S14 OR S15 OR S16 OR S17 OR S18 OR S19 OR S20	31,552
S20	TI (heatstroke* or hypertherm* or overheat* or over-heat*) OR AB (heatstroke* or hypertherm* or overheat* or over-heat*)	3,909
S19	TI ((heat or thermal) N3 (stress* or ill* or sick* or fatigu* or exhaust* or strain* or exert* or cramp* or collaps* or syncop* or prostrat* or stroke* or expos* or shock* or disorder* or injur* or diseas* or trauma*)) OR AB ((heat or thermal) N3 (stress* or ill* or sick* or fatigu* or exhaust* or strain* or exert* or cramp* or collaps* or syncop* or prostrat* or stroke* or expos* or shock* or disorder* or injur* or diseas* or trauma*))	7,328
S18	TI (climate* N2 chang*) OR AB (climate* N2 chang*)	6,058

S17	TI (global warming or greenhouse effect*) OR AB (global warming or greenhouse effect*)	952
S16	TI (heatwave* or heat-wave*) OR AB (heatwave* or heat-wave*)	670
S15	TI ((extreme* or intens* or high* or summer* or severe*) N2 (heat* or warm* or hot* or temperature*)) OR AB ((extreme* or intens* or high* or summer* or severe*) N2 (heat* or warm* or hot* or temperature*))	5,755
S14	TI ((hot* or warm*) N4 (temperature* or condition# or environment* or weather* or indoor* or spell# or climate*)) OR AB ((hot* or warm*) N4 (temperature* or condition# or environment* or weather* or indoor* or spell# or climate*))	3,251
S13	(MH "Heat Stress Disorders+")	2,532
S11	TI (heatstroke* or sunstroke* or sun-stroke* or hypertherm* or overheat* or over-heat*) OR AB (heatstroke* or sunstroke* or sun-stroke* or hypertherm* or overheat* or over-heat*)	3,848
S10	TI ((heat or thermal) N3 (stress* or ill* or sick* or fatigu* or exhaust* or strain* or exert* or cramp* or collaps* or syncop* or prostrat* or stroke* or expos* or shock* or disorder* or injur* or diseas* or trauma* or load*)) OR AB ((heat or thermal) N3 (stress* or ill* or sick* or fatigu* or exhaust* or strain* or exert* or cramp* or collaps* or syncop* or prostrat* or stroke* or expos* or shock* or disorder* or injur* or diseas* or trauma* or load*))	7,266
S9	(MH "Heat Stress Disorders+")	2,470
S8	TI ("global warming" or "greenhouse effect") OR AB ("global warming" or "greenhouse effect")	880
S7	(MH "Greenhouse Effect")	1,346
S6	TI (climate* N3 (tropic* or desert* or hot* or warm* or arid*)) OR AB (climate* N3 (tropic* or desert* or hot* or warm* or arid*))	688
S5	TI (heatwave* or heat-wave*) OR AB (heatwave* or heat-wave*)	641
S4	TI ((extreme* or intens*) N2 (heat* or warm* or hot*)) OR AB ((extreme* or intens*) N2 (heat* or warm* or hot*))	752
S3	TI (high* N2 temperature?) OR AB (high* N2 temperature?)	2,833
S2	TI ((hot* or warm*) N4 (temperature* or condition? or environment* or weather* or setting* or space* or outdoor* or indoor* or office* or building* or spell?)) OR AB ((hot* or warm*) N4 (temperature* or condition? or environment* or weather* or setting* or space* or outdoor* or indoor* or office* or building* or spell?))	2,996
S1	(MH "Heat")	8,772

Table 4. Global Health (EBSCOhost) Search Strategy

#	Searches	Results
S58	S16 AND S31 AND S57	363
S57	S32 OR S33 OR S34 OR S35 OR S36 OR S37 OR S38 OR S39 OR S40 OR S41 OR S42 OR S43 OR S44 OR S45 OR S46 OR S47 OR S48 OR S49 OR S50 OR S51 OR S52 OR S53 OR S54 OR S55 OR S56	55,157
S56	TI ((light* or loose* or air* or less*) N4 (cloth* or dress* or apparel* or attire* or garment*)) OR AB ((light* or loose* or air* or less*) N4 (cloth* or dress* or apparel* or attire* or garment*))	304
S55	TI ((drink* or ingest*) N2 water#) OR AB ((drink* or ingest*) N2 water#)	45,640
S54	TI ((ice# or icy or cold* or cool*) N4 (drink* or beverage* or hydrat* or fluid* or pack#)) OR AB ((ice# or icy or cold* or cool*) N4 (drink* or beverage* or hydrat* or fluid* or pack#))	983
S53	TI ((cool* or cold* or ice* or icy) N3 (immers* or bath* or shower*)) OR AB ((cool* or cold* or ice* or icy) N3 (immers* or bath* or shower*))	424

S52	TI ((wet* or damp*) N3 (cloth* or spong*)) OR AB ((wet* or damp*) N3 (cloth* or spong*))	82
S51	TI ((cool* or cold* or phase change or ice* or icy or cryo*) N4 (vest# or garment* or attire* or cloth* or apparel or gear or scarf or scarves or towel* or personal* or wear* or therap* or pack# or compress* or spray* or mist*)) OR AB ((cool* or cold* or phase change or ice* or icy or cryo*) N4 (vest# or garment* or attire* or cloth* or apparel or gear or scarf or scarves or towel* or personal* or wear* or therap* or pack# or compress* or spray* or mist*))	816
S50	TI ((heat pump* or ductless) N6 (cool* or cold*)) OR AB ((heat pump* or ductless) N6 (cool* or cold*))	12
S49	TI ((close* or closing or shut* or draw* or pull* or open*) N4 (curtain* or blind# or window* or shade*)) OR AB ((close* or closing or shut* or draw* or pull* or open*) N4 (curtain* or blind# or window* or shade*))	1,072
S48	TI (window* N4 (tint* or block* or alumin#um or blind# or curtain* or film# or cover*)) OR AB (window* N4 (tint* or block* or alumin#um or blind# or curtain* or film# or cover*))	120
S47	TI ((glaz* or film* or tint* or window*) N3 (photochromic or thermochromic)) OR AB ((glaz* or film* or tint* or window*) N3 (photochromic or thermochromic))	1
S46	TI (swamp N2 (cooler* or box*)) OR AB (swamp N2 (cooler* or box*))	3
S45	TI ((evaporative or air or desert) N2 (cooler* or cooling)) OR AB ((evaporative or air or desert) N2 (cooler* or cooling))	335
S44	TI (fan or fans) OR AB (fan or fans)	1,404
S43	TI (climate* N1 control*) OR AB (climate* N1 control*)	174
S42	TI (air N1 (condition* or revitali* or central)) OR AB (air N1 (condition* or revitali* or central))	2,213
S41	(DE "ventilation" OR DE "artificial ventilation" OR DE "natural ventilation") AND (TI (cool* or cold*) OR AB (cool* or cold*))	121
S40	DE "cryotherapy"	377
S39	DE "air conditioning"	419
S38	DE "hydration"	1,697
S37	DE "fans"	63
S36	DE "coolers"	163
S35	DE "cooling systems"	235
S34	DE "double glazing"	1
S33	DE "glazing"	14
S32	DE "windows"	119
S31	S17 OR S18 OR S19 OR S20 OR S21 OR S22 OR S23 OR S24 OR S25 OR S26 OR S27 OR S28 OR S29 OR S30	42,565
S30	TI (heatstroke* or hypertherm* or overheat* or over-heat*) OR AB (heatstroke* or hypertherm* or overheat* or over-heat*)	1,796
S29	TI ((heat or thermal) N3 (stress* or ill* or sick* or fatigu* or exhaust* or strain* or exert* or cramp* or collaps* or syncop* or prostrat* or stroke* or expos* or shock* or disorder* or injur* or diseas* or trauma*)) OR AB ((heat or thermal) N3 (stress* or ill* or sick* or fatigu* or exhaust* or strain* or exert* or cramp* or collaps* or syncop* or prostrat* or stroke* or expos* or shock* or disorder* or injur* or diseas* or trauma*))	11,378
S28	TI (global warming or greenhouse effect*) OR AB (global warming or greenhouse effect*)	2,835
S27	TI (heatwave* or heat-wave*) OR AB (heatwave* or heat-wave*)	1,140

S26	TI ((extreme* or intens* or high* or summer* or severe*) N2 (heat* or warm* or hot* or temperature*)) OR AB ((extreme* or intens* or high* or summer* or severe*) N2 (heat* or warm* or hot* or temperature*))	22,027
S25	TI ((hot* or warm*) N4 (temperature* or condition# or environment* or weather* or indoor* or spell# or climate*)) OR AB ((hot* or warm*) N4 (temperature* or condition# or environment* or weather* or indoor* or spell# or climate*))	6,598
S24	DE "hyperthermia"	646
S23	DE "heat injury"	105
S22	DE "heat shock"	1,342
S21	DE "heat exhaustion"	211
S20	DE "heat stress"	3,103
S19	DE "greenhouse effect"	169
S18	DE "global warming"	2,016
S17	DE "heat"	3,490
S16	S1 OR S2 OR S3 OR S4 OR S5 OR S6 OR S7 OR S8 OR S9 OR S10 OR S11 OR S12 OR S13 OR S14 OR S15	314,356
S15	TI (independent* N2 (live# or living)) OR AB (independent* N2 (live# or living))	1,360
S14	TI (within door* or withindoor*) OR AB (within door* or withindoor*)	27
S13	TI ((space* or room* or setting*) N3 (personal or confined or living)) OR AB ((space* or room* or setting*) N3 (personal or confined or living))	2,473
S12	TI (house* or housing* or home# or facility or facilities or residential* or residence# or apartment* or condo* or accommodation* or dwelling* or domicile* or building* or lodging* or shelter# or indoor* or in-door*) OR AB (house* or housing* or home# or facility or facilities or residential* or residence# or apartment* or condo* or accommodation* or dwelling* or domicile* or building* or lodging* or shelter# or indoor* or in-door*)	310,288
S11	DE "buildings"	5,436
S10	DE "retirement homes"	97
S9	DE "mobile homes"	8
S8	DE "single family housing"	10
S7	DE "rural housing"	57
S6	DE "public housing"	231
S5	DE "homes"	4,262
S4	DE "dwellings"	7,914
S3	DE "cooperative housing"	600
S2	DE "cooperative housing"	0
S1	DE "housing"	2,313
