# Supplementary material for: Updating Health Canada’s Heat-Health Messages for the Environment and Climate Change Canada Heat Warning System: A Collaboration with Canadian Experts
Source: Int J Environ Res Public Health. 2025 Aug 13;22(8):1266. doi: 10.3390/ijerph22081266 (PMC12386431; doi:10.3390/ijerph22081266)
Supplement: Supplementary file 1 [file ijerph-22-01266-s001.zip › IJERPH_Supplementary Material File S3_Statement Iterations English.pdf]

# Updating Health Canada's Heat-Health Messages for the Environment and Climate Change Canada Heat Warning System: A Consultation Process with Canadian Experts

Supplemental File C: Statement Iterations

ENGLISH

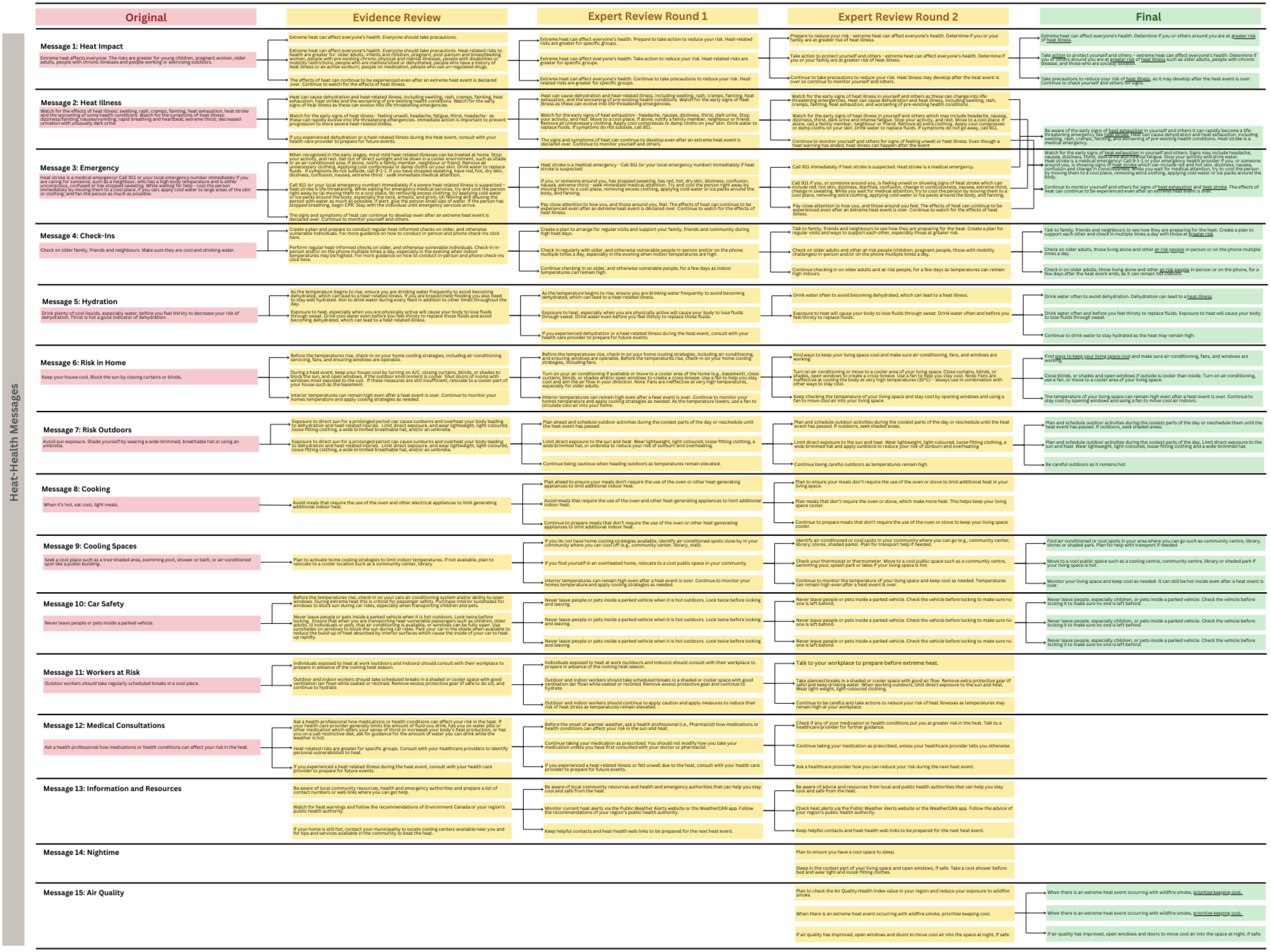
Heat-Health Messages
Original
Evidence Review
Expert Review Round 1
Expert Review Round 2
Final
Message 1: Heat Impact
Message 2: Heat Illness
Message 3: Emergency
Message 4: Check-Ins
Message 5: Hydration
Message 6: Risk in Home
Message 7: Risk Outdoors
Message 8: Cooking
Message 9: Cooling Spaces
Message 10: Car Safety
Message 11: Workers at Risk
Message 12: Medical Consultations
Message 13: Information and Resources
Message 14: Nightime
Message 15: Air Quality

## Message 1: Heat Impact

| | Released in Advance of a Heat Event | Released During a Heat Event | Released After a Heat Event Ends |
|---|---|---|---|
| **Original** | - | Extreme heat affects everyone. The risks are greater for young children, pregnant women, older adults, people with chronic illnesses and people working or exercising outdoors. | - |
| **Revised** (Evidence Review) | Extreme heat can affect everyone's health. Everyone should take precautions. | Extreme heat can affect everyone's health. Everyone should take precautions. Heat-related risks to health are greater for: older adults, infants and children, pregnant, post-partum and breastfeeding women, people with pre-existing chronic physical and mental illnesses, people with disabilities or mobility restrictions, people who are malnourished or dehydrated, people who have a history of heat illness or an active sunburn, people on medication, people who use un-regulated drugs. | The effects of heat can continue to be experienced even after an extreme heat event is declared over. Continue to watch for the effects of heat illness. |
| **Revised** (Expert Review Round 1) | Extreme heat can affect everyone's health. Prepare to take action to reduce your risk. Heat-related risks are greater for specific groups. | Extreme heat can affect everyone's health. Take action to reduce your risk. Heat-related risks are greater for specific groups. | Extreme heat can affect everyone's health. Continue to take precautions to reduce your risk. Heat-related risks are greater for specific groups. |
| **Revised** (Expert Review Round 2) | Prepare to reduce your risk - extreme heat can affect everyone's health. Determine if you or your family are at greater risk of heat illness. | Take action to protect yourself and others - extreme heat can affect everyone's health. Determine if you or your family are at greater risk of heat illness. | Continue to take precautions to reduce your risk. Heat illness may develop after the heat event is over so continue to monitor yourself and others. |
| **Final** | Extreme heat can affect everyone's health. Determine if you or others around you are at greater risk of heat illness. | Take action to protect yourself and others – extreme heat can affect everyone's health. Determine if you or others around you are at greater risk of heat illness such as older adults, people with chronic disease, and those who are socially isolated. | Take precautions to reduce your risk of heat illness, as it may develop after the heat event is over. Continue to check yourself and others for signs. |

## Message 2: Heat Illness

| | Released in Advance of a Heat Event | Released During a Heat Event | Released After a Heat Event Ends |
|---|---|---|---|
| **Original** | Watch for the effects of heat illness: swelling, rash, cramps, fainting, heat exhaustion, heat stroke and the worsening of some health conditions. | Watch for the symptoms of heat illness: dizziness/fainting; nausea/vomiting; rapid breathing and heartbeat; extreme thirst; decreased urination with unusually dark urine. | - |
| **Revised** (Evidence Review) | Heat can cause dehydration and heat-related illness, including swelling, rash, cramps, fainting, heat exhaustion, heat stroke and the worsening of pre-existing health conditions. Watch for the early signs of heat illness as these can evolve into life-threatening emergencies. | Watch for the early signs of heat illness - feeling unwell, headache, fatigue, thirst, headache - as these can rapidly evolve into life-threatening emergencies. Immediate action is important to prevent progression to more severe heat-related illness. | If you experienced dehydration or a heat-related illness during the heat event, consult with your health care provider to prepare for future events. |
| **Revised** (Expert Review Round 1) | Heat can cause dehydration and heat-related illness, including swelling, rash, cramps, fainting, heat exhaustion, and the worsening of pre-existing health conditions. Watch for the early signs of heat illness as these can evolve into life-threatening emergencies. | Watch for the early signs of heat exhaustion - headache, nausea, dizziness, thirst, dark urine. Stop your activity, and rest. Move to a cool place. If alone, notify a family member, neighbour or friend. Remove all unnecessary clothing. Apply cool compresses or damp cloths on your skin. Drink water to replace fluids. If symptoms do not subside, call 911. | The signs and symptoms of heat can continue to develop even after an extreme heat event is declared over. Continue to monitor yourself and others. |
| **Revised** (Expert Review Round 2) | Watch for the early signs of heat illness in yourself and others as these can change into life-threatening emergencies. Heat can cause dehydration and heat illness, including swelling, rash, cramps, fainting, heat exhaustion, and worsening of pre-existing health conditions. | Watch for the early signs of heat illness in yourself and others which may include headache, nausea, dizziness, thirst, dark urine and intense fatigue. Stop your activity, and rest. Move to a cool place. If alone, call a family member, neighbour or friend. Remove all extra clothing. Apply cool compresses or damp cloths on your skin. Drink water to replace fluids. If symptoms do not go away, call 911. | Continue to monitor yourself and others for signs of feeling unwell or heat illness. Even though a heat warning has ended, heat illness can happen after the event |
| **Final** | Combined with Statement 3 | Combined with Statement 3 | Combined with Statement 3 |

## Message 3: Emergency

| | Released in Advance of a Heat Event | Released During a Heat Event | Released After a Heat Event Ends |
|---|---|---|---|
| **Original** | - | Heat stroke is a medical emergency! Call 911 or your local emergency number immediately if you are caring for someone, such as a neighbour, who has a high body temperature and is either unconscious, confused or has stopped sweating. While waiting for help - cool the person immediately by moving them to a cool place, if you can; apply cold water to large areas of the skin or clothing; and fan the person as much as possible. | - |
| **Revised** (Evidence Review) | When recognized in the early stages, most mild heat-related illnesses can be treated at home. Stop your activity, and rest. Get out of direct sunlight and lie down in a cooler environment, such as shade or an air-conditioned area. If alone, notify a family member, neighbour or friend. Remove all unnecessary clothing. Applying cool compresses or damp cloths on your skin. Drink water to replace fluids. If symptoms do not subside, call 9-1-1. If you have stopped sweating, have red, hot, dry skin, dizziness, confusion, nausea, extreme thirst - seek immediate medical attention. | Call 911 (or your local emergency number) immediately if a severe heat related illness is suspected – heat stroke is life threatening. While waiting for emergency medical services, try and cool the person right away by (a) moving them to a cool place, (b) removing excess clothing, (c) applying cold water or ice packs around the body, especially the neck, armpits, and groin, (d) fanning and dousing the person with water as much as possible. If alert, give the person small sips of water. If the person has stopped breathing, begin CPR. Stay with the individual until emergency services arrive. | The signs and symptoms of heat can continue to develop even after an extreme heat event is declared over. Continue to monitor yourself and others. |
| **Revised** (Expert Review Round 1) | Heat stroke is a medical emergency - Call 911 (or your local emergency number) immediately if heat stroke is suspected. | If you, or someone around you, has stopped sweating, has red, hot, dry skin, dizziness, confusion, nausea, extreme thirst - seek immediate medical attention. Try and cool the person right away by moving them to a cool place, removing excess clothing, applying cold water or ice packs around the body, and fanning. | Pay close attention to how you, and those around you, feel. The effects of heat can continue to be experienced even after an extreme heat event is declared over. Continue to watch for the effects of heat illness. |

| | | | |
|---|---|---|---|
| **Revised** (Expert Review Round 2) | Call 911 immediately if heat stroke is suspected. Heat stroke is a medical emergency. | Call 911 if you, or someone around you, is feeling unwell or showing signs of heat stroke which can include red, hot skin, dizziness, diarrhea, confusion, change in consciousness, nausea, extreme thirst, change in sweating. While you wait for medical attention, try to cool the person by moving them to a cool place, removing extra clothing, applying cold water or ice packs around the body, and fanning. | Pay close attention to how you, and those around you feel. The effects of heat can continue to be experienced even after an extreme heat event is over. Continue to watch for the effects of heat illness. |
| **Final** | Be aware of the early signs of heat exhaustion in yourself and others it can rapidly become a life-threatening emergency like heat stroke. Heat can cause dehydration and heat exhaustion, including swelling, rash, cramps, fainting, and worsening of pre-existing health conditions. Heat stroke is a medical emergency. | Watch for the early signs of heat exhaustion in yourself and others. Signs may include headache, nausea, dizziness, thirst, dark urine and intense fatigue. Stop your activity and drink water.<br>Heat stroke is a medical emergency! Call 9-1-1 or your emergency health provider if you, or someone around you, is showing signs of heat stroke which can include red and hot skin, dizziness, nausea, confusion and change in consciousness. While you wait for medical attention, try to cool the person by moving them to a cool place, removing extra clothing, applying cold water or ice packs around the body. | Continue to monitor yourself and others for signs of heat exhaustion and heat stroke. The effects of heat can continue to be experienced even after an extreme heat event is over. |

## Message 4: Check-Ins

| | Released in Advance of a Heat Event | Released During a Heat Event | Released After a Heat Event Ends |
|---|---|---|---|
| **Original** | - | Check on older family, friends and neighbours. Make sure they are cool and drinking water. | - |
| **Revised** (Evidence Review) | Create a plan and prepare to conduct regular heat-informed checks on older, and otherwise vulnerable individuals. For more guidance on how to conduct in-person and phone check-ins click here. | Perform regular heat-informed checks on older, and otherwise vulnerable individuals. Check-in in-person and/or on the phone multiple times a day, especially in the evening when indoor temperatures may be highest. For more guidance on how to conduct in-person and phone check-ins click here. | - |
| **Revised** (Expert Review Round 1) | Create a plan to arrange for regular visits and support your family, friends and community during high heat days. | Check-in regularly with older, and otherwise vulnerable people in-person and/or on the phone multiple times a day, especially in the evening when indoor temperatures are high. | Continue checking in on older, and otherwise vulnerable people, for a few days as indoor temperatures can remain high. |
| **Revised** (Expert Review Round 2) | Talk to family, friends and neighbours to see how they are preparing for the heat. Create a plan for regular visits and ways to support each other, especially those at greater risk. | Check on older adults and other at-risk people (children, pregnant people, those with mobility challenges) in-person and/or on the phone multiple times a day. | Continue checking in on older adults and at-risk people, for a few days as temperatures can remain high indoors. |
| **Final** | Talk to family, friends and neighbours to see how they are preparing for the heat. Create a plan to support each other and check in multiple times a day with those at greater risk. | Check on older adults, those living alone and other at-risk people in-person or on the phone multiple times a day. | Check in on older adults, those living alone and other at-risk people in-person or on the phone, for a few days after the heat event ends, as it can remain hot indoors. |

## Message 5: Hydration

| | Released in Advance of a Heat Event | Released During a Heat Event | Released After a Heat Event Ends |
|---|---|---|---|
| **Original** | - | Drink plenty of cool liquids, especially water, before you feel thirsty to decrease your risk of dehydration. Thirst is not a good indicator of dehydration. | - |
| **Revised** (Evidence Review) | As the temperature begins to rise, ensure you are drinking water frequently to avoid becoming dehydrated, which can lead to a heat-related illness. If you are breast/chest-feeding you also need to stay well hydrated. Aim to drink water during every feed in addition to other times throughout the day. | Exposure to heat, especially when you are physically active will cause your body to lose fluids through sweat. Drink cool water even before you feel thirsty to replace those fluids and avoid becoming dehydrated, which can lead to a heat-related illness. | - |
| **Revised** (Expert Review Round 1) | As the temperature begins to rise, ensure you are drinking water frequently to avoid becoming dehydrated, which can lead to a heat-related illness. | Exposure to heat, especially when you are physically active will cause your body to lose fluids through sweat. Drink water even before you feel thirsty to replace those fluids. | If you experienced dehydration or a heat-related illness during the heat event, consult with your health care provider to prepare for future events. |
| **Revised** (Expert Review Round 2) | Drink water often to avoid becoming dehydrated, which can lead to a heat illness. | Exposure to heat will cause your body to lose fluids through sweat. Drink water often and before you feel thirsty to replace fluids. | - |
| **Final** | Drink water often to avoid dehydration. Dehydration can lead to a heat illness. | Drink water often and before you feel thirsty to replace fluids. Exposure to heat will cause your body to lose fluids through sweat. | Continue to drink water to stay hydrated as the heat may remain high. |

## Message 6: Risk in the Home

| | Released in Advance of a Heat Event | Released During a Heat Event | Released After a Heat Event Ends |
|---|---|---|---|
| **Original** | - | Keep your house cool. Block the sun by closing curtains or blinds. | - |
| **Revised** (Evidence Review) | Before the temperatures rise, check-in on your home cooling strategies, including air-conditioning servicing, fans, and ensuring windows are operable. | During a heat event, keep your house cool by turning on A/C, closing curtains, blinds, or shades to block the sun, and open windows, if the outdoor environment is cooler. Shut doors of rooms with windows most exposed to the sun. If these measures are still insufficient, relocate to a cooler part of your house such as the basement. | Interior temperatures can remain high even after a heat event is over. Continue to monitor your homes temperature and apply cooling strategies as needed. |
| **Revised** (Expert Review Round 1) | Before the temperatures rise, check-in on your home cooling strategies, including air-conditioning, and ensuring windows are operable.<br><br>Before the temperatures rise, check-in on your home cooling strategies, including fans. | Turn on your air conditioning if available or move to a cooler area of the home (e.g., basement), close curtains, blinds, or shades and/or open windows to create a cross-breeze.<br><br>Use a fan to help you stay cool and aim the air flow in your direction. Note: Fans are ineffective at very high temperatures, especially for older adults. | Interior temperatures can remain high even after a heat event is over. Continue to monitor your homes temperature and apply cooling strategies as needed.<br><br>As the temperature lowers, use a fan to circulate cool air into your home. |
| **Revised** (Expert Review Round 2) | Find ways to keep your living space cool and make sure air-conditioning, fans, and windows are working. | Turn on air conditioning or move to a cooler area of your living space. Close curtains, blinds, or shades, open windows to create a cross-breeze. Use a fan to help you stay cool. Note: Fans are ineffective at cooling the body at very high temperatures (35°C) – always use in combination with other ways to stay cool. | Keep checking the temperature of your living space and stay cool by opening windows and using a fan to move cool air into your living space. |
| **Final** | Find ways to keep your living space cool and make sure air-conditioning, fans, and windows are working. | Close blinds, or shades and open windows if outside is cooler than inside. Turn on air conditioning, use a fan, or move to a cooler area of your living space. | The temperature of your living space can remain high even after a heat event is over. Continue to stay cool by opening windows and using a fan to move cool air indoors. |

## Message 7: Risk Outdoors

| | Released in Advance of a Heat Event | Released During a Heat Event | Released After a Heat Event Ends |
|---|---|---|---|
| **Original** | Avoid sun exposure. Shade yourself by wearing a wide-brimmed, breathable hat or using an umbrella. | Shade yourself with an umbrella or a wide-brimmed hat. | - |
| **Revised** (Evidence Review) | Exposure to direct sun for a prolonged period can cause sunburns and overheat your body leading to dehydration and heat-related injuries. Limit direct exposure, and wear lightweight, light-coloured, loose-fitting clothing, a wide brimmed breathable hat, and/or an umbrella. | Exposure to direct sun for a prolonged period can cause sunburns and overheat your body leading to dehydration and heat-related injuries. Limit direct exposure, and wear lightweight, light-coloured, loose-fitting clothing, a wide brimmed breathable hat, and/or an umbrella. | - |
| **Revised** (Expert Review Round 1) | Plan ahead and schedule outdoor activities during the coolest parts of the day or reschedule until the heat event has passed. | Limit direct exposure to the sun and heat. Wear lightweight, light-coloured, loose-fitting clothing, a wide brimmed hat, or umbrella to reduce your risk of sunburn and overheating. | Continue being cautious when heading outdoors as temperatures remain elevated. |
| **Revised** (Expert Review Round 2) | Plan and schedule outdoor activities during the coolest parts of the day or reschedule until the heat event has passed. If outdoors, seek shaded areas. | Limit direct exposure to the sun and heat. Wear lightweight, light-coloured, loose-fitting clothing, a wide brimmed hat and apply sunblock to reduce your risk of sunburn and overheating. | Continue being careful outdoors as temperatures remain high. |
| **Final** | Plan and schedule outdoor activities during the coolest parts of the day or reschedule them until the heat event has passed. If outdoors, seek shaded areas. | Plan and schedule outdoor activities during the coolest parts of the day. Limit direct exposure to the sun and heat. Wear lightweight, light-coloured, loose-fitting clothing and a wide-brimmed hat. | Be careful outdoors as it remains hot. |

## Message 8: Cooking

| | Released in Advance of a Heat Event | Released During a Heat Event | Released After a Heat Event Ends |
|---|---|---|---|
| **Original** | - | When it's hot, eat cool, light meals. | - |
| **Revised** (Evidence Review) | - | Avoid meals that require the use of the oven and other electrical appliances to limit generating additional indoor heat. | - |
| **Revised** (Expert Review Round 1) | Plan ahead to ensure your meals don't require the use of the oven or other heat-generating appliances to limit additional indoor heat. | Avoid meals that require the use of the oven and other heat-generating appliances to limit additional indoor heat. | Continue to prepare meals that don't require the use of the oven or other heat-generating appliances to limit additional indoor heat. |
| **Revised** (Expert Review Round 2) | Plan to ensure your meals don't require the use of the oven or stove to limit additional heat in your living space. | Plan meals that don't require the oven or stove, which make more heat. This helps keep your living space cooler. | Continue to prepare meals that don't require the use of the oven or stove to keep your living space cooler. |
| **Final** | Removed | Removed | Removed |

## Message 9: Cooling Spaces

| | Released in Advance of a Heat Event | Released During a Heat Event | Released After a Heat Event Ends |
|---|---|---|---|
| **Original** | - | Seek a cool place such as a tree-shaded area, swimming pool, shower or bath, or air-conditioned spot like a public building. | - |
| **Revised** (Evidence Review) | Plan to activate home cooling strategies to limit indoor temperatures. If not available, plan to relocate to a cooler location such as a community center, library. | - | - |
| **Revised** (Expert Review Round 1) | If you do not have home cooling strategies available, identify air-conditioned spots close by in your community where you can cool off (e.g., community center, library, mall). | If you find yourself in an overheated home, relocate to a cool public space in your community. | Interior temperatures can remain high even after a heat event is over. Continue to monitor your homes temperature and apply cooling strategies as needed. |
| **Revised** (Expert Review Round 2) | Identify air-conditioned or cool spots in your community where you can go (e.g., community center, library, stores, shaded parks). Plan for transport help if needed. | Check your thermostat or thermometer. Move to a cool public space such as a community centre, swimming pool, splash park or lakes if your living space is hot. | Continue to monitor the temperature of your living space and keep cool as needed. Temperatures can remain high even after a heat event is over. |
| **Final** | Find air-conditioned or cool spots in your area where you can go such as community centre, library, stores or shaded park. Plan for help with transport if needed. | Move to a cool public space such as a cooling centre, community centre, library or shaded park if your living space is hot. | Monitor your living space and keep cool as needed. It can still be hot inside even after a heat event is over. |

## Message 10: Car Safety

| | Released in Advance of a Heat Event | Released During a Heat Event | Released After a Heat Event Ends |
|---|---|---|---|
| **Original** | - | Never leave people or pets inside a parked vehicle. | - |
| **Revised** (Evidence Review) | Before the temperatures rise, check-in on your cars air conditioning system and/or ability to open windows. During extreme heat this is critical for passenger safety. Purchase interior sunshades for windows to block sun during car rides, especially when transporting children and pets. | Never leave people or pets inside a parked vehicle when it is hot outdoors. Look twice before locking. Ensure that when you are transporting heat-vulnerable passengers such as children, older adults, ill individuals or pets, that air conditioning is available, or windows can be fully open. Use sunshades on windows to block the sun during car rides. Park your car in the shade when available to reduce the build-up of heat absorbed by interior surfaces which cause the inside of your car to heat up rapidly. | - |
| **Revised** (Expert Review Round 1) | Never leave people or pets inside a parked vehicle when it is hot outdoors. Look twice before locking and leaving. | Never leave people or pets inside a parked vehicle when it is hot outdoors. Look twice before locking and leaving. | Never leave people or pets inside a parked vehicle when it is hot outdoors. Look twice before locking and leaving. |
| **Revised** (Expert Review Round 2) | Never leave people or pets inside a parked vehicle. Check the vehicle before locking to make sure no one is left behind. | Never leave people or pets inside a parked vehicle. Check the vehicle before locking to make sure no one is left behind. | Never leave people or pets inside a parked vehicle. Check the vehicle before locking to make sure no one is left behind. |
| **Final** | Never leave people, especially children, or pets inside a parked vehicle. Check the vehicle before locking it to make sure no one is left behind. | Never leave people, especially children, or pets inside a parked vehicle. Check the vehicle before locking to make sure no one is left behind. | Never leave people, especially children, or pets inside a parked vehicle. Check the vehicle before locking it to make sure no one is left behind. |

## Message 11: Workers

| | Released in Advance of a Heat Event | Released During a Heat Event | Released After a Heat Event Ends |
|---|---|---|---|
| **Original** | - | Outdoor workers should take regularly scheduled breaks in a cool place. | - |
| **Revised** (Evidence Review) | Individuals exposed to heat at work (outdoors and indoors) should consult with their workplace to prepare in advance of the coming heat season. | Outdoor and indoor workers should take scheduled breaks in a shaded or cooler space with good ventilation (air flow) while seated or reclined. Remove excess protective gear (if safe to do so), and continue to hydrate. | - |
| **Revised** (Expert Review Round 1) | Individuals exposed to heat at work (outdoors and indoors) should consult with their workplace to prepare in advance of the coming heat season. | Outdoor and indoor workers should take scheduled breaks in a shaded or cooler space with good ventilation (air flow) while seated or reclined. Remove excess protective gear and continue to hydrate. | Outdoor and indoor workers should continue to apply caution and apply measures to reduce their risk of heat stress as temperatures remain elevated. |
| **Revised** (Expert Review Round 2) | Talk to your workplace to prepare before extreme heat. | Take planned breaks in a shaded or cooler space with good air flow. Remove extra protective gear (if safe) and keep drinking water. When working outdoors, limit direct exposure to the sun and heat. Wear light-weight, light-coloured clothing. | Continue to be careful and take actions to reduce your risk of heat illnesses as temperatures may remain high at your workplace. |
| **Final** | Removed | Removed | Removed |

## Message 12: Medical

| | Released in Advance of a Heat Event | Released During a Heat Event | Released After a Heat Event Ends |
|---|---|---|---|
| **Original** | - | Ask a health professional how medications or health conditions can affect your risk in the heat. | - |
| **Revised** (Evidence Review) | Ask a health professional how medications or health conditions can affect your risk in the heat. If your health care provider generally limits the amount of fluid you drink, has you on water pills or other medication which alters your sense of thirst or increases your body's heat production, or has you on a salt-restrictive diet, ask for guidance for the amount of water you can drink while the weather is hot. | Heat-related risks are greater for specific groups. Consult with your healthcare providers to identify personal vulnerabilities to heat. | If you experienced a heat-related illness during the heat event, consult with your health care provider to prepare for future events. |
| **Revised** (Expert Review Round 1) | Before the onset of warmer weather, ask a health professional (i.e., Pharmacist) how medications or health conditions can affect your risk in the sun and heat. | Continue taking your medication as prescribed. You should not modify how you take your medication unless you have first consulted with your doctor or pharmacist. | If you experienced a heat-related illness or felt unwell due to the heat, consult with your health care provider to prepare for future events. |
| **Revised** (Expert Review Round 2) | Check if any of your medication or health conditions put you at greater risk in the heat. Talk to a healthcare provider for further guidance. | Continue taking your medication as prescribed, unless your healthcare provider tells you otherwise. | Ask a healthcare provider how you can reduce your risk during the next heat event. |
| **Final** | Removed | Removed | Removed |

## Message 13: Information and Resources

| | Released in Advance of a Heat Event | Released During a Heat Event | Released After a Heat Event Ends |
|---|---|---|---|
| **Original** | - | - | - |
| **Revised** (Evidence Review) | - | - | - |
| **Revised** (Expert Review Round 1) | Be aware of local community resources, health and emergency authorities and prepare a list of contact numbers or web links where you can get help. | Watch for heat warnings and follow the recommendations of Environment Canada or your region's public health authority. | If your home is still hot, contact your municipality to locate cooling centers available near you and for tips and services available in the community to beat the heat. |
| **Revised** (Expert Review Round 2) | Be aware of local community resources and health and emergency authorities that can help you stay cool and safe from the heat. | Monitor current heat alerts via the Public Weather Alerts website or the WeatherCAN app. Follow the recommendations of your region's public health authority. | Keep helpful contacts and heat-health web links to be prepared for the next heat event. |
| **Final** | Be aware of advice and resources from local and public health authorities that can help you stay cool and safe from the heat. | Check heat alerts via the Public Weather Alerts website or the WeatherCAN app. Follow the advice of your region's public health authority. | Keep helpful contacts and heat-health web links to be prepared for the next heat event. |

## Message 14: Air Quality

| | Released in Advance of a Heat Event | Released During a Heat Event | Released After a Heat Event Ends |
|---|---|---|---|
| **Original** | - | - | - |
| **Revised** (Evidence Review) | - | - | - |
| **Revised** (Expert Review Round 1) | - | - | - |
| **Revised** (Expert Review Round 2) | Plan to check the Air Quality Health Index value in your region and reduce your exposure to wildfire smoke. | When there is an extreme heat event occurring with wildfire smoke, prioritize keeping cool. | If air quality has improved, open windows and doors to move cool air into the space at night, if safe. |
| **Final** | When there is an extreme heat event occurring with wildfire smoke, prioritize keeping cool. | When there is an extreme heat event occurring with wildfire smoke, prioritize keeping cool. | If air quality has improved, open windows and doors to move cool air into the space at night, if safe. |

## Message 15: Nighttime

| | Released in Advance of a Heat Event | Released During a Heat Event | Released After a Heat Event Ends |
|---|---|---|---|
| **Original** | - | - | - |
| **Revised** (Evidence Review) | - | - | - |
| **Revised** (Expert Review Round 1) | - | - | - |
| **Revised** (Expert Review Round 2) | Plan to ensure you have a cool space to sleep. | Sleep in the coolest part of your living space and open windows, if safe. Take a cool shower before bed and wear light and loose-fitting clothes. | - |
| **Final** | Removed | Removed | Removed |
