# Supplementary material for: Updating Health Canada’s Heat-Health Messages for the Environment and Climate Change Canada Heat Warning System: A Collaboration with Canadian Experts
Source: Int J Environ Res Public Health. 2025 Aug 13;22(8):1266. doi: 10.3390/ijerph22081266 (PMC12386431; doi:10.3390/ijerph22081266)
Supplement: Supplementary file 1 [file ijerph-22-01266-s001.zip › IJERPH_Supplementary Material File S4_Statement Iterations French.pdf]

Updating Health Canada’s Heat-Health Messages for the Environment and Climate Change Canada Heat Warning System: A Consultation Process with Canadian Experts

Supplemental File D: Statement Iterations

FRENCH

Message 1: Heat Impact

	Diffusés avec les alertes précoces	Diffusés avec l'alerte de chaleur	Diffusés lorsque l'événement prend fin
**Original**	-	La chaleur extrême affecte tout le monde. Les risques sont plus importants pour les jeunes enfants, les femmes enceintes, les personnes âgées, les personnes souffrant de maladies chroniques et les personnes qui travaillent ou font de l'exercice à l'extérieur.	-
**Révisé** (Examen des éléments de preuve)	La chaleurs extrême peuvent affecter la santé de chacun. Chacun doit prendre des précautions.	La chaleur extrême peut affecter la santé de chacun. Tout le monde devrait prendre des précautions. Les risques liés à la chaleur sont plus importants pour les personnes âgées, les nourrissons et les enfants, les femmes enceintes, les femmes en post-partum et celles qui allaitent, les personnes souffrant de maladies physiques et mentales chroniques préexistantes, les personnes handicapées ou à mobilité réduite, les personnes souffrant de malnutrition ou de déshydratation, les personnes ayant des antécédents de maladies liées à la chaleur ou un coup de soleil actif, les personnes sous traitement médicamenteux, les personnes qui consomment des drogues non réglementées.	Les effets de la chaleur peuvent continuer à se faire sentir même après la fin d'un épisode de chaleur extrême. Continuez à surveiller les effets des maladiea assoiées à la chaleur.
**Révisé** (Consultation d'experts 1ère ronde)	La chaleur extrême peut avoir une incidence sur la santé de tous. Préparez-vous à prendre des mesures afin de réduire vos risques. Les risques relatifs à la chaleur sont plus importants pour certains groupes.	La chaleur extrême peut avoir une incidence sur la santé de tous. Prenez des mesures afin de réduire vos risques. Les risques relatifs à la chaleur sont plus importants pour certains groupes.	La chaleur extrême peut avoir une incidence sur la santé de tous. Continuez à prendre des précautions afin de réduire vos risques. Les risques relatifs à la chaleur sont plus importants pour certains groupes.
**Révisé** (Consultation d'experts 2ième ribde)	Prenez des mesures pour réduire les risques pour vous – la chaleur extrême peut avoir des effets sur la santé de tous. Déterminez	Prenez des mesures pour vous protéger et protéger les autres – la chaleur extrême peut avoir des effets sur la santé de tous. Déterminez si vous ou votre famille êtes	Continuez à prendre des précautions afin de réduire vos risques. Les maladies associées à la chaleur peuvent se manifester après la fin de l'événement de chaleur; vous devez

	si vous ou votre famille êtes plus à risque de maladies associées à la chaleur.	plus à risque de maladies associées à la chaleur.	donc continuer de vous surveiller et de surveiller les autres.
**Finale**	La chaleur extrême peut avoir une incidence sur la santé de tous. Déterminez si vous ou d'autres personnes de votre entourage êtes plus à risque de maladie liée à la chaleur.	Prenez des mesures pour vous protéger et protéger les autres – la chaleur extrême peut avoir des effets sur la santé de tous. Déterminez si vous ou d'autres personnes de votre entourage êtes plus à risque de maladie liée à la chaleur, comme les personnes âgées, les personnes atteintes de maladies chroniques et les personnes isolées socialement.	Prenez des précautions pour réduire votre risque de maladie liée à la chaleur, car elle peut se manifester après l'événement de chaleur. Continuez de vous surveiller et de surveiller les autres pour déceler les signes.

Message 2: Heat Illness

	Diffusés avec les alertes précoces	Diffusés avec l'alerte de chaleur	Diffusés lorsque l'événement prend fin
**Original**	Surveillez les effets de la chaleur : gonflement, éruption cutanée, crampes, évanouissement, épuisement dû à la chaleur, coup de chaleur et aggravation de certains problèmes de santé.	Surveillez les symptômes des maladies associées à la chaleur : vertiges, nausées, vomissements, accélération de la respiration et du rythme cardiaque, soif extrême, diminution de la miction avec des urines inhabituellement foncées.	-
**Révisé** (Examen des éléments de preuve)	La chaleur peut entraîner une déshydratation et des maladies associées à la chaleur, notamment des gonflements, des éruptions cutanées, des crampes, des évanouissements, un épuisement dû à la chaleur, un coup de chaleur et l'aggravation de problèmes de santé préexistants. Surveillez les premiers signes d'une maladie associée à la chaleur, car ils peuvent évoluer vers des situations d'urgence potentiellement mortelles.	Surveillez les premiers signes d'une maladie associée à la chaleur - malaise, maux de tête, fatigue, soif, maux de tête - car ils peuvent rapidement évoluer vers des situations d'urgence mettant en jeu le pronostic vital. Il est important d'agir immédiatement pour empêcher l'évolution vers une maladie associée à la chaleur plus grave.	Si vous avez souffert de déshydratation ou d'une maladie associée à la chaleur pendant l'épisode de chaleur, consultez votre prestataire de soins de santé pour vous préparer à de futurs événements.
**Révisé** (Consultation d'experts 1ère ronde)	La chaleur peut provoquer une déshydratation et des maladies associées à la chaleur, notamment des gonflements, des éruptions cutanées, des crampes, des évanouissements, un épuisement attribuable à la chaleur et l'aggravation de problèmes de santé préexistants. Surveillez les premiers signes d'une maladie attribuable à la chaleur, car ils peuvent évoluer vers des situations d'urgence potentiellement mortelles.	Surveillez les premiers signes d'épuisement en raison de la chaleur : maux de tête, nausées, vertiges, soif, urines foncées. Cessez la pratique de votre activité et reposez-vous. Déplacez-vous dans un endroit frais. Si vous êtes seul, prévenez un membre de votre famille, un voisin ou encore un ami. Retirez tous les vêtements inutiles. Appliquez des compresses froides ou encore des linges humides sur votre peau. Buvez de l'eau afin de remplacer les liquides. Si les symptômes ne disparaissent pas, composez le 911.	Les signes et symptômes de la chaleur peuvent continuer à se développer même après la fin d'un événement de chaleur extrême. Continuez à vous surveiller et à surveiller les autres.
**Révisé** (Consultation d'experts 2ième ribde)	Surveillez les premiers signes d'une maladie attribuable à la chaleur chez vous et chez les autres, car ils peuvent évoluer vers des situations d'urgence	Surveillez les premiers signes d'une maladie attribuable à la chaleur chez vous et chez les autres, lesquels signes peuvent comprendre les suivants : maux de tête,	Continuez de vous surveiller et de surveiller les autres pour déceler tout signe de malaise ou de maladie attribuable à la chaleur. Des maladies attribuables à la

	potentiellement mortelles. La chaleur peut provoquer une déshydratation et des maladies associées à la chaleur, notamment des gonflements, des éruptions cutanées, des crampes, des évanouissements, un épuisement attribuable à la chaleur et l'aggravation de problèmes de santé préexistants.	nausées, vertiges, soif, urines foncées et intense fatigue. Cessez la pratique de votre activité et reposez-vous. Déplacez-vous dans un endroit frais. Si vous êtes seul, prévenez un membre de votre famille, un voisin ou encore un ami. Retirez tous les vêtements superflus. Appliquez des compresses froides ou encore des linges humides sur votre peau. Buvez de l'eau afin de remplacer les liquides. Si vos symptômes persistent, composez le 911.	chaleur peuvent se manifester même après la fin de l'avertissement de chaleur.
**Finale**	Combiné avec la déclaration 3	Combiné avec la déclaration 3	Combiné avec la déclaration 3

Message 3: Emergency

	Diffusés avec les alertes précoces	Diffusés avec l'alerte de chaleur	Diffusés lorsque l'événement prend fin
**Original**	-	Le coup de chaleur est une urgence médicale ! Appelez immédiatement le 911 ou votre numéro d'urgence local si vous vous occupez d'une personne, par exemple un voisin, dont la température corporelle est élevée et qui est inconsciente, confuse ou qui a cessé de transpirer. En attendant les secours, rafraîchissez immédiatement la personne en la déplaçant dans un endroit frais, si vous le pouvez ; appliquez de l'eau froide sur de grandes parties de la peau ou sur les vêtements ; et ventilez la personne autant que possible.	-
**Révisé** (Examen des éléments de preuve)	Lorsqu'elles sont détectées dès les premiers stades, la plupart des maladies légères associées à la chaleur peuvent être traitées à domicile. Arrêtez votre activité et reposez-vous. Éloignez-vous de la lumière directe du soleil et allongez-vous dans un environnement plus frais, par exemple à l'ombre ou dans un endroit climatisé. Si vous êtes seul, prévenez un membre de votre famille, un voisin ou un ami. Enlevez tous les vêtements inutiles. Appliquez des compresses fraîches ou des linges humides sur votre peau. Boire de l'eau pour remplacer les liquides. Si les symptômes ne disparaissent pas, appelez le 9-1-1. Si vous avez cessé de transpirer, si votre peau est rouge, chaude et sèche, si vous avez des vertiges, de la confusion, des nausées ou une soif extrême, consultez immédiatement un médecin.	Appelez immédiatement le 911 (ou le numéro d'urgence local) si vous soupçonnez une maladie grave associée à la chaleur - le coup de chaleur met la vie en danger. En attendant les services médicaux d'urgence, essayez de refroidir la personne immédiatement en (a) la déplaçant dans un endroit frais, (b) en enlevant l'excès de vêtements, (c) en appliquant de l'eau froide ou des poches de glace autour du corps, en particulier au niveau du cou, des aisselles et de l'aine, (d) en l'éventant et en l'aspergeant d'eau autant que possible. Si la personne est alerte, donnez-lui de petites gorgées d'eau. Si la personne a cessé de respirer, commencez la réanimation cardio-pulmonaire. Restez avec la personne jusqu'à l'arrivée des services d'urgence.	Les signes et symptômes de la chaleur peuvent continuer à se développer même après la fin d'un épisode de chaleur extrême. Continuez à vous surveiller et à surveiller les autres.

**Révisé** (Consultation d'experts 1ère ronde)	Un coup de chaleur est une urgence médicale – Composez immédiatement le 911 ou votre numéro d'urgence local si vous soupçonnez souffrir d'un coup de chaleur.	Si vous, ou quelqu'un de votre entourage, avez cessé de transpirer, avez la peau rouge, chaude et sèche, des vertiges, de la confusion, des nausées, une soif extrême, veillez à consulter immédiatement un médecin. Essayez de rafraîchir la personne immédiatement en la déplaçant dans un endroit frais, en enlevant les vêtements superflus, en appliquant de l'eau froide ou des poches de glace autour du corps et en l'éventant.	Prêtez attention à ce que vous ressentez et à ce que ressentent les personnes qui vous entourent. Les effets de la chaleur peuvent continuer à se faire sentir même après la fin d'un événement de chaleur extrême. Continuez à surveiller les effets des maladies attribuables à la chaleur.
**Révisé** (Consultation d'experts 2ième ribde)	Composez immédiatement le 911 si vous croyez présenter un coup de chaleur. Un coup de chaleur est une urgence médicale.	Composez le 911 si vous, ou quelqu'un de votre entourage, ressentez un malaise ou présentez des signes de coup de chaleur, par exemple une peau rouge et chaude, des étourdissements, de la diarrhée, de la confusion, un niveau de conscience altéré, des nausées, une soif extrême ou des changements au niveau de la transpiration. En attendant l'arrivée des secours médicaux, essayez de rafraîchir la personne en la déplaçant dans un endroit frais, en retirant ses vêtements superflus, en appliquant de l'eau froide ou des blocs réfrigérants autour de son corps et en l'éventant.	Prêtez attention à ce que vous ressentez et à ce que ressentent les personnes qui vous entourent. Les effets de la chaleur peuvent continuer à se faire sentir même après la fin d'un événement de chaleur extrême. Continuez à surveiller les effets des maladies attribuables à la chaleur.
**Finale**	Soyez conscient des premiers signes d'épuisement dû à la chaleur que vous ou d'autres personnes pourriez présenter, car cela peut rapidement devenir une urgence potentiellement mortelle comme un coup de chaleur. La chaleur peut provoquer une déshydratation et un épuisement dû à la chaleur, notamment des gonflements, des éruptions cutanées, des crampes, des évanouissements et l'aggravation de	Surveillez les premiers signes d'épuisement dû à la chaleur que vous ou d'autres personnes pourriez présenter. Les signes peuvent comprendre des maux de tête, des nausées, des étourdissements, la soif, une urine foncée et une fatigue intense. Cessez votre activité et buvez de l'eau. Un coup de chaleur est une urgence médicale! Composez le 9-1-1 ou communiquez avec votre fournisseur de soins de santé d'urgence si vous ou	Continuez de vous surveiller et de surveiller les autres pour déceler les signes d'épuisement dû à la chaleur et de coup de chaleur. Les effets de la chaleur peuvent continuer à se faire sentir même après la fin d'un événement de chaleur extrême.

	problèmes de santé préexistants. Un coup de chaleur est une urgence médicale.	quelqu'un de votre entourage présentez des signes de coup de chaleur, notamment une peau rouge et chaude, des étourdissements, des nausées, de la confusion et un niveau de conscience altéré. En attendant l'arrivée des secours médicaux, essayez de rafraîchir la personne en la déplaçant dans un endroit frais, en retirant ses vêtements superflus, en appliquant de l'eau froide ou des blocs réfrigérants autour de son corps.	

Message 4: Check-Ins

	Diffusés avec les alertes précoces	Diffusés avec l'alerte de chaleur	Diffusés lorsque l'événement prend fin
**Original**	-	Prenez des nouvelles des membres de votre famille, de vos amis et de vos voisins les plus âgés. Assurez-vous qu'ils sont au frais et qu'ils boivent de l'eau.	-
**Révisé** (Examen des éléments de preuve)	Élaborer un plan et se préparer à effectuer des visites régulières chez des personnes âgées et des autres personnes vulnérables à la chaleur. Pour plus d'informations sur la manière de procéder à des visites en personne ou par téléphone, cliquez ici.	Prenez couramment des nouvelles des personnes âgées et des autres personnes vulnérables en fonction de la chaleur. Prenez couramment des nouvelles en personne et/ou par téléphone plusieurs fois par jour, en particulier le soir, lorsque les températures intérieures peuvent être les plus élevées. Pour plus de conseils sur la manière de procéder à des visites en personne et par téléphone, cliquez ici.	-
**Révisé** (Consultation d'experts 1ère ronde)	Préparez un plan pour organiser des visites périodiques et soutenir votre famille, vos amis et votre communauté pendant les jours de forte chaleur.	Prenez couramment des nouvelles des personnes âgées ou vulnérables, en personne ou par téléphone, plusieurs fois par jour, en particulier le soir lorsque la température intérieure est élevée.	Continuez à surveiller les personnes âgées et les autres personnes vulnérables pendant quelques jours, car les températures intérieures peuvent demeurer élevées.
**Révisé** (Consultation d'experts 2ième ribde)	Parlez avec votre famille, vos amis et vos voisins pour savoir comment ils se préparent à la chaleur. Établissez un plan prévoyant des visites régulières et des moyens de vous soutenir mutuellement, en particulier les personnes les plus à risque.	Prenez des nouvelles des personnes âgées et des autres personnes à risque (enfants, femmes enceintes, personnes à mobilité réduite), en personne ou par téléphone, plusieurs fois par jour.	Continuez à surveiller les personnes âgées et les personnes à risque pendant quelques jours, car les températures peuvent demeurer élevées à l'intérieur.
**Finale**	Parlez avec votre famille, vos amis et vos voisins pour savoir comment ils se préparent à la chaleur. Créez un plan pour vous soutenir mutuellement et prenez des nouvelles plusieurs fois par jour des personnes les plus à risque.	Prenez des nouvelles des personnes âgées, de celles qui vivent seules et d'autres personnes à risque, en personne ou par téléphone, plusieurs fois par jour.	Prenez des nouvelles des personnes âgées, de celles qui vivent seules et d'autres personnes à risque, en personne ou par téléphone, pendant quelques jours après la fin de l'événement de chaleur, car il est possible qu'il fasse toujours chaud à l'intérieur.

Message 5: Hydration

	Diffusés avec les alertes précoces	Diffusés avec l'alerte de chaleur	Diffusés lorsque l'événement prend fin
**Original**	-	Buvez beaucoup de liquides frais, en particulier de l'eau, avant d'avoir soif, afin de réduire le risque de déshydratation. La soif n'est pas un bon indicateur de déshydratation.	-
**Révisé** (Examen des éléments de preuve)	Lorsque la température commence à augmenter, veillez à boire fréquemment de l'eau afin d'éviter la déshydratation, qui peut entraîner une maladie associée à la chaleur. Si vous allaitez, vous devez également rester bien hydratée. Essayez de boire de l'eau à chaque tétée et à d'autres moments de la journée.	L'exposition à la chaleur, en particulier lorsque vous êtes physiquement actif, entraîne une perte de liquides par la transpiration. Buvez de l'eau fraîche avant même d'avoir soif pour remplacer ces liquides et éviter de vous déshydrater, ce qui peut entraîner une maladie associée à la chaleur.	-
**Révisé** (Consultation d'experts 1ère ronde)	Lorsque la température commence à augmenter, veillez à boire fréquemment de l'eau, en vue d'éviter la déshydratation, qui peut entraîner une maladie associée à la chaleur.	L'exposition à la chaleur, en particulier lorsque vous êtes physiquement actif, entraîne une perte de liquides au moyen de la transpiration. Buvez de l'eau avant même d'avoir soif afin de remplacer ces liquides.	Si vous avez souffert de déshydratation ou d'une maladie associée à la chaleur pendant l'événement de chaleur, consultez votre professionnel de la santé pour vous préparer à de futurs événements.
**Révisé** (Consultation d'experts 2ième ribde)	Buvez souvent de l'eau pour éviter la déshydratation, laquelle peut provoquer une maladie associée à la chaleur.	L'exposition à la chaleur cause une perte de liquides par la transpiration. Buvez de l'eau souvent et avant même d'avoir soif, afin de remplacer ces liquides.	-
**Finale**	Buvez de l'eau souvent pour éviter de vous déshydrater. La déshydratation peut entraîner une maladie liée à la chaleur.	Buvez de l'eau souvent et avant même d'avoir soif, afin de remplacer ces liquides. L'exposition à la chaleur cause une perte de liquides par la transpiration.	Continuez à boire de l'eau pour rester hydraté, car la température peut demeurer élevée.

Message 6: Risk in the Home

	Diffusés avec les alertes précoces	Diffusés avec l'alerte de chaleur	Diffusés lorsque l'événement prend fin
**Original**	-	Gardez votre maison au frais. Bloquez le soleil en fermant les rideaux ou les stores.	-
**Révisé** (Examen des éléments de preuve)	Avant que les températures n'augmentent, vérifiez les stratégies de refroidissement de votre maison, y compris l'entretien de la climatisation, les ventilateurs et l'ouverture des fenêtres.	Pendant un épisode de chaleur, gardez votre maison fraîche en allumant la climatisation, en fermant les rideaux, les stores ou les toiles pour bloquer le soleil, et en ouvrant les fenêtres si l'environnement extérieur est plus frais. Fermez les portes des pièces dont les fenêtres sont les plus exposées au soleil. Si ces mesures ne suffisent toujours pas, déménagez dans une partie plus fraîche de votre maison, comme le sous-sol.	Les températures intérieures peuvent rester élevées même après la fin d'un épisode de chaleur. Continuez à surveiller la température de votre maison et appliquez des stratégies de refroidissement si nécessaire.
**Révisé** (Consultation d'experts 1ère ronde)	Avant que les températures n'augmentent, informez-vous au sujet des stratégies pour refroidir votre domicile, y compris l'entretien des systèmes de climatisation, les ventilateurs et l'ouverture des fenêtres.<br><br>Avant que les températures n'augmentent, vérifiez les stratégies de rafraîchissement de votre domicile, y compris la présence de ventilateurs.	Faites fonctionner la climatisation de l'air, si vous en disposez d'un tel système, ou encore déplacez-vous dans un endroit plus frais du domicile (p. ex. le sous-sol), fermez les rideaux, les stores ou les toiles et/ou ouvrez les fenêtres pour créer un courant d'air transversal.<br><br>Utilisez un ventilateur pour vous aider à rester au frais et orientez le flux d'air dans votre direction. Remarque : les ventilateurs sont inefficaces à des températures très élevées, en particulier pour les personnes âgées.	Les températures intérieures peuvent demeurer élevées même après la fin d'un événement de chaleur. Continuez à surveiller la température de votre domicile et appliquez des stratégies de rafraîchissement si nécessaire.<br><br>Lorsque la température baisse, utilisez un ventilateur pour faire circuler l'air frais dans votre domicile.
**Révisé** (Consultation d'experts 2ième ribde)	Trouvez des moyens de garder votre espace de vie au frais et veillez à ce que la climatisation et les ventilateurs fonctionnent et à ce que les fenêtres s'ouvrent.	Mettez la climatisation en marche ou déplacez-vous dans un endroit plus frais de votre espace de vie. Fermez les rideaux, les stores ou les toiles et ouvrez les fenêtres pour créer un courant d'air transversal. Utilisez un ventilateur pour vous aider à rester au frais. Remarque : à des températures très élevées (35 °C), les	Vérifiez la température dans votre espace de vie et restez au frais en ouvrant les fenêtres et en utilisant un ventilateur pour faire entrer de l'air frais à l'intérieur.

		ventilateurs sont inefficaces pour refroidir le corps – utilisez-les toujours avec d’autres moyens de rester au frais.	
**Finale**	Trouvez des moyens de garder votre espace de vie au frais et veillez à ce que la climatisation et les ventilateurs fonctionnent et à ce que les fenêtres s’ouvrent.	Fermez les stores ou les toiles, et ouvrez les fenêtres s’il fait plus frais à l’extérieur qu’à l’intérieur. Mettez la climatisation en marche, utilisez un ventilateur ou déplacez-vous dans un endroit plus frais de votre espace de vie.	La température de votre espace de vie peut demeurer élevée même après la fin d’un événement de chaleur. Continuez à rester au frais en ouvrant les fenêtres et en utilisant un ventilateur pour déplacer l’air frais à l’intérieur.

Message 7: Risk Outdoors

	Diffusés avec les alertes précoces	Diffusés avec l'alerte de chaleur	Diffusés lorsque l'événement prend fin
**Original**	Évitez l'exposition au soleil. Mettez-vous à l'ombre en portant un chapeau à larges bords et perméable à l'air ou en utilisant un parapluie.	Protégez-vous à l'aide d'un parapluie ou d'un chapeau à large bord.	-
**Révisé** (Examen des éléments de preuve)	L'exposition directe au soleil pendant une période prolongée peut provoquer des coups de soleil et une surchauffe du corps, entraînant une déshydratation et des blessures associées à la chaleur. Limitez l'exposition directe et portez des vêtements légers, clairs et amples, un chapeau respirant à large bord et/ou un parapluie.	L'exposition directe au soleil pendant une période prolongée peut provoquer des coups de soleil et une surchauffe du corps, entraînant une déshydratation et des blessures assoiées à la chaleur. Limitez l'exposition directe et portez des vêtements légers, clairs et amples, un chapeau respirant à large bord et/ou un parapluie.	-
**Révisé** (Consultation d'experts 1ère ronde)	Planifiez vos activités en plein air pendant les heures les plus fraîches de la journée ou reportez-les jusqu'à ce que l'événement de chaleur soit passé.	Limitez l'exposition directe au soleil et à la chaleur. Portez des vêtements légers, clairs et amples, un chapeau à large bord ou encore utilisez un parapluie pour réduire les risques de coups de soleil et de surchauffe.	Restez prudent lorsque vous sortez à l'extérieur de votre domicile, car les températures demeurent élevées.
**Révisé** (Consultation d'experts 2ième ribde)	Planifiez vos activités en plein air pendant les heures les plus fraîches de la journée ou reportez-les après la fin de l'événement de chaleur. À l'extérieur, recherchez les endroits ombragés.	Limitez l'exposition directe au soleil et à la chaleur. Portez des vêtements légers, pâles et amples et un chapeau à large bord et appliquez un écran solaire pour réduire vos risques de coups de soleil et de surchauffe.	Restez prudent à l'extérieur, car les températures demeurent élevées.
**Finale**	Planifiez vos activités en plein air pendant les heures les plus fraîches de la journée ou reportez-les après la fin de l'événement de chaleur. À l'extérieur, recherchez les endroits ombragés.	Planifiez et organisez vos activités en plein air aux heures les plus fraîches de la journée. Limitez l'exposition directe au soleil et à la chaleur. Portez des vêtements légers, pâles et amples et un chapeau à larges bords.	Soyez prudent à l'extérieur, car la température demeure élevée.

Message 8: Cooking

	Diffusés avec les alertes précoces	Diffusés avec l'alerte de chaleur	Diffusés lorsque l'événement prend fin
**Original**	-	Lorsqu'il fait chaud, mangez des repas frais et légers.	-
**Révisé** (Examen des éléments de preuve)	-	Évitez les repas qui nécessitent l'utilisation du four et d'autres appareils électriques afin de ne pas générer de chaleur supplémentaire à l'intérieur.	-
**Révisé** (Consultation d'experts 1ère ronde)	Planifiez vos repas pour qu'ils ne nécessitent pas l'utilisation du four ou d'autres appareils générateurs de chaleur, en vue de limiter la chaleur intérieure supplémentaire.	Évitez les repas qui nécessitent l'utilisation du four et d'autres appareils générateurs de chaleur en vue de limiter la chaleur intérieure supplémentaire.	Continuez à préparer des repas qui ne nécessitent pas l'utilisation du four ou d'autres appareils générateurs de chaleur en vue de limiter la chaleur intérieure supplémentaire.
**Révisé** (Consultation d'experts 2ième ribde)	Planifiez vos repas de manière à ce qu'ils ne nécessitent pas l'utilisation du four ou de la cuisinière afin de limiter l'apport supplémentaire de chaleur dans votre espace de vie.	Planifiez des repas qui ne requièrent pas l'utilisation du four ou de la cuisinière, lesquels génèrent plus de chaleur. Cela vous aidera à garder votre espace de vie plus frais.	Continuez à préparer des repas qui ne requièrent pas l'utilisation du four ou de la cuisinière afin de garder votre espace de vie plus frais.
**Finale**	Supprimé	Supprimé	Supprimé

Message 9: Cooling Spaces

	Diffusés avec les alertes précoces	Diffusés avec l'alerte de chaleur	Diffusés lorsque l'événement prend fin
**Original**	-	Recherchez un endroit frais tel qu'une zone ombragée, une piscine, une douche ou un bain, ou un endroit climatisé tel qu'un bâtiment public.	-
**Révisé** (Examen des éléments de preuve)	Prévoyez d'activer des stratégies de refroidissement à domicile pour limiter les températures intérieures. Si ce n'est pas possible, prévoyez de vous installer dans un endroit plus frais, tel qu'un centre communautaire ou une bibliothèque.	-	-
**Révisé** (Consultation d'experts 1ère ronde)	Si vous ne disposez pas de stratégies de rafraîchissement à domicile, déterminez les endroits climatisés près de votre communauté où vous pouvez aller vous rafraîchir (p. ex. centre communautaire, bibliothèque, centre commercial).	Si vous vous trouvez dans un domicile surchauffé, rendez-vous dans un espace public frais de votre communauté.	Les températures intérieures peuvent demeurer élevées même après la fin d'un événement de chaleur. Continuez à surveiller la température de votre domicile et appliquez des stratégies de rafraîchissement si nécessaire.
**Révisé** (Consultation d'experts 2ième ribde)	Déterminez les endroits climatisés ou frais dans votre communauté où vous pouvez aller (p. ex. centre communautaire, bibliothèque, magasins, parcs ombragés). Prévoyez une aide au transport, s'il y a lieu.	Vérifiez votre thermostat ou thermomètre. S'il fait chaud dans votre espace de vie, allez dans un lieu public frais comme un centre communautaire, une piscine, un parc avec jeux d'eau ou un lac.	Continuez à surveiller la température à l'intérieur de votre espace de vie et à le garder au frais, s'il y a lieu. Les températures peuvent demeurer élevées même une fois l'événement de chaleur terminé.
**Finale**	Trouvez des endroits climatisés ou frais dans votre entourage où vous pouvez aller, comme un centre communautaire, une bibliothèque, des magasins ou des parcs ombragés. Prévoyez une aide au transport, s'il y a lieu.	S'il fait chaud dans votre espace de vie, allez dans un lieu public frais comme un centre de rafraîchissement, un centre communautaire, une bibliothèque ou des parcs ombragés.	Surveillez votre espace de vie et restez au frais au besoin. Il peut encore faire chaud à l'intérieur même après la fin d'un événement de chaleur.

Message 10: Car Safety

	Diffusés avec les alertes précoces	Diffusés avec l'alerte de chaleur	Diffusés lorsque l'événement prend fin
**Original**	-	Ne laissez jamais des personnes ou des animaux à l'intérieur d'un véhicule en stationnement.	-
**Révisé** (Examen des éléments de preuve)	Avant que les températures n'augmentent, vérifiez le système de climatisation de votre voiture et/ou sa capacité à ouvrir les fenêtres. En cas de chaleur extrême, cela est essentiel pour la sécurité des passagers. Achetez des pare-soleil intérieurs pour les fenêtres afin de bloquer le soleil pendant les trajets en voiture, en particulier lorsque vous transportez des enfants et des animaux de compagnie.	Ne laissez jamais des personnes ou des animaux à l'intérieur d'un véhicule en stationnement lorsqu'il fait chaud à l'extérieur. Regardez à deux fois avant de verrouiller. Lorsque vous transportez des passagers vulnérables à la chaleur, tels que des enfants, des personnes âgées, des personnes malades ou des animaux domestiques, assurez-vous que la climatisation est disponible ou que les fenêtres peuvent être complètement ouvertes. Utilisez des pare-soleil sur les fenêtres pour bloquer le soleil pendant les trajets en voiture. Stationnez votre voiture à l'ombre lorsque c'est possible afin de réduire l'accumulation de chaleur absorbée par les surfaces intérieures, ce qui fait chauffer rapidement l'intérieur de la voiture.	-
**Révisé** (Consultation d'experts 1ère ronde)	Ne laissez jamais des personnes ou encore des animaux à l'intérieur d'un véhicule garé lorsqu'il fait chaud à l'extérieur. Regardez à deux reprises avant de fermer à clé et de partir.	Ne laissez jamais des personnes ou encore des animaux à l'intérieur d'un véhicule garé lorsqu'il fait chaud à l'extérieur. Regardez à deux reprises avant de fermer à clé et de partir.	Ne laissez jamais des personnes ou encore des animaux à l'intérieur d'un véhicule garé lorsqu'il fait chaud à l'extérieur. Regardez à deux reprises avant de fermer à clé et de partir.
**Révisé** (Consultation d'experts 2ième ribde)	Ne laissez jamais des personnes ou des animaux à l'intérieur d'un véhicule garé. Vérifiez le véhicule avant de le verrouiller afin de vous assurer qu'il ne reste personne à l'intérieur.	Ne laissez jamais des personnes ou des animaux à l'intérieur d'un véhicule garé. Vérifiez le véhicule avant de le verrouiller afin de vous assurer qu'il ne reste personne à l'intérieur.	Ne laissez jamais des personnes ou des animaux à l'intérieur d'un véhicule garé. Vérifiez le véhicule avant de le verrouiller afin de vous assurer qu'il ne reste personne à l'intérieur.
**Finale**	Ne laissez jamais des personnes, en particulier des enfants, ou des animaux à	Ne laissez jamais des personnes, en particulier des enfants, ou des animaux à	Ne laissez jamais des personnes, en particulier des enfants, ou des animaux à

	l’intérieur d’un véhicule garé. Vérifiez le véhicule avant de le verrouiller afin de vous assurer qu’il ne reste personne à l’intérieur.	l’intérieur d’un véhicule garé. Vérifiez le véhicule avant de le verrouiller afin de vous assurer qu’il ne reste personne à l’intérieur.	l’intérieur d’un véhicule garé. Vérifiez le véhicule avant de le verrouiller afin de vous assurer qu’il ne reste personne à l’intérieur.

Message 11: Workers

	Diffusés avec les alertes précoces	Diffusés avec l'alerte de chaleur	Diffusés lorsque l'événement prend fin
**Original**	-	Les travailleurs à l'extérieur devraient faire des pauses régulières dans un endroit frais.	-
**Révisé** (Examen des éléments de preuve)	Les personnes exposées à la chaleur sur leur lieu de travail (à l'extérieur et à l'intérieur) devraient consulter leur employeur pour se préparer à la saison des chaleurs qui s'annonce.	Les travailleurs à l'extérieur et à l'intérieur devraient faire des pauses programmées dans un espace ombragé ou plus frais avec une bonne ventilation (circulation de l'air), en position assise ou allongée. Retirez l'équipement de protection excédentaire (si vous pouvez le faire en toute sécurité) et continuez à vous hydrater.	-
**Révisé** (Consultation d'experts 1ère ronde)	Les personnes exposées à la chaleur sur leur lieu de travail (à l'extérieur et à l'intérieur) devraient consulter leur employeur afin de se préparer à la saison chaude qui s'annonce.	Les travailleurs à l'extérieur et à l'intérieur devraient prendre des pauses programmées dans un espace ombragé ou plus frais avec une bonne ventilation (flux d'air), idéalement en position assise ou allongée. Retirez l'équipement de protection excédentaire et continuez à vous hydrater.	Les travailleurs à l'extérieur et à l'intérieur doivent continuer à faire preuve de prudence et à appliquer des mesures visant à réduire le risque de stress attribuable à la chaleur, car les températures restent élevées.
**Révisé** (Consultation d'experts 2ième ribde)	Parlez à votre employeur afin de vous préparer avant une chaleur extrême.	Prévoyez des pauses dans un lieu plus frais ou ombragé où il y a une bonne circulation d'air. Retirez l'équipement de protection excédentaire (s'il est sécuritaire de le faire) et continuez à boire de l'eau. Lorsque vous travaillez à l'extérieur, limitez votre exposition directe au soleil et à la chaleur. Portez des vêtements légers et de couleur pâle.	Restez prudent et prenez des mesures pour réduire votre risque de maladies liées à la chaleur, car les températures peuvent demeurer élevées sur votre lieu de travail.
**Finale**	Supprimé	Supprimé	Supprimé

Message 12: Medical Consultation

	Diffusés avec les alertes précoces	Diffusés avec l'alerte de chaleur	Diffusés lorsque l'événement prend fin
**Original**	-	Demandez à un professionnel de la santé si vos médicaments ou votre état de santé peuvent avoir une incidence sur les risques associés à la chaleur.	-
**Révisé** (Examen des éléments de preuve)	Demandez à un professionnel de la santé comment les médicaments ou l'état de santé peuvent influer sur les risques associés à la chaleur. Si votre professionnel de la santé limite généralement la quantité de liquide que vous buvez, vous fait prendre des comprimés d'eau ou d'autres médicaments qui modifient votre sensation de soif ou augmentent la production de chaleur de votre corps, ou vous fait suivre un régime pauvre en sel, demandez-lui de vous conseiller sur la quantité d'eau que vous pouvez boire lorsqu'il fait chaud.	Les risques associés à la chaleur sont plus importants pour certains groupes. Consultez vos prestataires de soins de santé pour identifier vos vulnérabilités personnelles à la chaleur.	Si vous avez souffert d'une maladie associée à la chaleur pendant l'épisode de chaleur, consultez votre prestataire de soins de santé pour vous préparer à d'autres épisodes.
**Révisé** (Consultation d'experts 1ère ronde)	Avant l'arrivée des beaux jours, demandez à un professionnel de la santé (p. ex. un pharmacien) comment les médicaments ou l'état de santé peuvent avoir une incidence sur les risques relatifs au soleil et à la chaleur.	Continuez à prendre vos médicaments, comme ils vous ont été prescrits. Vous ne devez pas modifier la façon dont vous prenez vos médicaments, et ce, sans avoir consulté au préalable votre médecin ou votre pharmacien.	Si vous avez souffert d'une maladie associée à la chaleur ou si vous vous êtes senti mal à cause de la chaleur, consultez votre professionnel de la santé afin de vous préparer à d'autres événements de chaleur.
**Révisé** (Consultation d'experts 2ième ribde)	Vérifiez si vos médicaments ou votre état de santé vous exposent à un risque accru en cas d'événement de chaleur. Consultez un professionnel de la santé pour obtenir plus de conseils à ce sujet.	Continuez à prendre vos médicaments tels qu'ils vous ont été prescrits, à moins d'avis contraire de la part de votre professionnel de la santé.	Demandez à un professionnel de la santé de vous indiquer comment réduire vos risques lors du prochain événement de chaleur.
**Finale**	Supprimé	Supprimé	Supprimé

Message 13: Information and Resources

	Diffusés avec les alertes précoces	Diffusés avec l'alerte de chaleur	Diffusés lorsque l'événement prend fin
**Original**	-	-	-
**Révisé** (Examen des éléments de preuve)	-	-	-
**Révisé** (Consultation d'experts 1ère ronde)	Renseignez-vous au sujet des ressources communautaires locales, des autorités de santé publique et des services d'urgence, et préparez une liste de numéros de personnes-ressources ou de liens Internet où vous pouvez obtenir de l'aide.	Surveillez les avertissements de chaleur et suivez les recommandations d'Environnement Canada ou encore de l'autorité de santé publique de votre région.	Si votre domicile est encore chaud, communiquez avec votre municipalité pour connaître les centres de rafraîchissement disponibles près de chez vous et pour obtenir des conseils et des services disponibles dans la communauté pour lutter contre la chaleur.
**Révisé** (Consultation d'experts 2ième ribde)	Renseignez-vous sur les ressources communautaires, les autorités sanitaires et les services d'urgence de votre région qui peuvent vous aider à rester au frais et à l'abri de la chaleur.	Surveillez les alertes de chaleur en cours en consultant le site Web sur les alertes météo publiques ou l'application MétéoCAN. Suivez les recommandations des autorités de santé publique de votre région.	Conservez sous la main des contacts utiles et des liens vers des sites Web traitant de la chaleur et de la santé, afin d'être prêt lors du prochain événement de chaleur.
**Finale**	Renseignez-vous sur les conseils et les ressources des autorités sanitaires locales et publiques de votre région qui peuvent vous aider à rester au frais et à l'abri de la chaleur.	Surveillez les alertes de chaleur en consultant le site Web sur les alertes météo publiques ou l'application MétéoCAN. Suivez les conseils des autorités de santé publique de votre région.	Conservez sous la main des contacts utiles et des liens vers des sites Web traitant de la chaleur et de la santé, afin d'être prêt lors du prochain événement de chaleur.

Message 14: Extreme Heat and Special Air Quality Statements or Air Quality Advisories

	**Diffusés avec l'événement d'alerte précoce** (de 24 à 48 heures avant un événement de chaleur)	**Diffusés avec l'alerte de chaleur** (pendant l'événement de chaleur)	**Diffusés avec une prolongation d'avertissement de 24 à 48 heures** (à la fin de l'alerte)
**Original**	-	-	-
**Révisé** (Examen des éléments de preuve)	-	-	-
**Révisé** (Consultation d'experts 1ère ronde)	-	-	-
**Révisé** (Consultation d'experts 2ième ribde)	Prévoyez de vérifier la cote air santé dans votre région et de réduire votre exposition à la fumée des feux de forêt.	Lors d'un événement de chaleur extrême qui s'accompagne de fumée de feux de forêt, la priorité est de rester au frais.	Si la qualité de l'air s'est améliorée, ouvrez les fenêtres et les portes pour faire entrer l'air frais pendant la nuit, si cela ne pose aucun danger.
**Finale**	Lors d'un événement de chaleur extrême qui s'accompagne de fumée de feux de forêt, la priorité est de rester au frais.	Lors d'un événement de chaleur extrême qui s'accompagne de fumée de feux de forêt, la priorité est de rester au frais.	Si la qualité de l'air s'est améliorée, ouvrez les fenêtres et les portes pour faire entrer l'air frais pendant la nuit, si cela ne pose aucun danger.

Message 15: Nighttime

	Diffusés avec les alertes précoces	Diffusés avec l'alerte de chaleur	Diffusés lorsque l'événement prend fin
**Original**	-	-	-
**Révisé** (Examen des éléments de preuve)	-	-	-
**Révisé** (Consultation d'experts 1ère ronde)	-	-	-
**Révisé** (Consultation d'experts 2ième ribde)	Prévoyez un espace frais pour dormir.	Dormez dans la partie la plus fraîche de votre espace de vie et ouvrez les fenêtres s'il n'y a aucun danger à le faire. Prenez une douche fraîche avant de vous coucher et portez des vêtements légers et amples.	-
**Finale**	Supprimé	Supprimé	Supprimé
