# Supplementary material for: Updating Health Canada’s Heat-Health Messages for the Environment and Climate Change Canada Heat Warning System: A Collaboration with Canadian Experts
Source: Int J Environ Res Public Health. 2025 Aug 13;22(8):1266. doi: 10.3390/ijerph22081266 (PMC12386431; doi:10.3390/ijerph22081266)
Supplement: Supplementary file 1 [file ijerph-22-01266-s001.zip › IJERPH_Supplementary Material File S5_Qualtrics Round 1.pdf]

English

Intro

Introduction

The Climate Change and Health Office (CCHO) at Health Canada is currently embarking on a multi-phased project to revise the heat-health messages included in Health Canada's communication materials and disseminated through the Environment and Climate Change Canada heat warning program. Informed by a comprehensive evidence review conducted by researchers at the University of Ottawa, CCHO has developed a revised list of messages which includes modifications to existing statements, the addition of new messages, as well as proposed groupings based on the timing of release (pre-heat event, during heat event and post-event). We are now seeking external feedback from leading public health experts and researchers across Canada to ensure the statements are comprehensive, action-oriented, evidence-based and reflect considerations such as readability, equity, and regional applicability.

Please complete your review and assessment by 11:59PM EST August 20, 2024. If you have any questions please email Melissa Gorman (Principal Investigator) at melissa.gorman@hc-sc.gc.ca or Emily Tetzlaff (Co-Principal Investigator) at emily.tetzlaff@hc-sc.gc.ca.

Note: You must click the "Submit" button at the end of the questionnaire for your responses to be recorded.

Terms and Conditions

Your professional input and ideas are being collected in order to seek your feedback on Health Canada's heat-health messages. We request some demographic information in order to ensure we are representing the views of various types of subject matter experts, including experts in public health, heat stress, environmental health, climate change, health equity or other related disciplines to allow for meaningful analysis of this consultation. Health Canada will be collecting your information via the Qualtrics tool and, as such, is subject to Qualtrics' privacy statement. Comments or quotes featured in the consultation summary and any resulting peer-reviewed publications and/or webinars will not be attributed to any specific individual or organization.

Please ensure that any written comments you provide are sufficiently general that you cannot be identified as the author and that individual identities are not disclosed.

Instructions for Completing the Consultation

- Answering each question is optional. You may skip any question for any reason.
- If you would like to go back to a previous question, you may do so by clicking on the "previous page" button.
- We anticipate that the consultation review will take you approximately 1-2 hours. You do not have to complete the consultation in one session, you may return to continue where you left off (before

the closing date) by using the same computer or device and browser you began the consultation with.

- You must click the “Submit” button at the end of the questionnaire for your responses to be recorded.

DemoQs

Demographic Questions

1. What group/organization do you work for?

[ ]

2. Which of the following best identifies your organization? (Select all that apply.)

- ☐ Academia/Research
- ☐ Regional Health Authority
- ☐ Provincial/Territorial Health Authority
- ☐ Federal Health Authority
- ☐ [ ] Other, please specify:

3. How long have you been employed in this field?

[ ]

4. What is your highest level of educational attainment?

- ○ Elementary School
- ○ Secondary School Diploma (or equivalent, e.g., GED)
- ○ College Certificate or Diploma
- ○ Bachelor’s Degree
- ○ Master’s Degree
- ○ Doctorate
- ○ Prefer Not To Disclose
- ○ [ ] Other, please specify:

5. Please provide 3 to 5 keywords which best describe your area of expertise (e.g., Heat Physiology, Climate Change, Environmental Health, Health Promotion, Equity).

Demographic Questions (Continued)

6. Which province/territory do you work in? (Select all that apply.)

- ☐ British Columbia
- ☐ Alberta
- ☐ Saskatchewan
- ☐ Manitoba
- ☐ Ontario
- ☐ Quebec
- ☐ New Brunswick
- ☐ Newfoundland and Labrador
- ☐ Nova Scotia
- ☐ Prince Edward Island
- ☐ Yukon
- ☐ Northwest Territories
- ☐ Nunavut
- ☐ Canada (Federal)
- ☐ ________ Other, please specify:

ConsultQs

Background Information

Weather alerts are signals used to heighten awareness and/or initiate preparation for action. In Canada, weather alerts are issued by Environment and Climate Change Canada (ECCC) for weather or environmental hazard events, including heat, that are either occurring, imminent, or forecast to develop.

The linked document provides the list of proposed messages Health Canada is proposing to use within the ECCC warning system which will be disseminated to the public during active heat warnings. As such, these messages should include the most important health-protective messages.

The proposed messages are organized to include a version that would be released when a heat event is anticipated, during a heat event and after the heat event is declared over:

- **Pre-heat event:** For release when a heat event is anticipated (e.g. 48-hours in advance of a heat event). The intent of these messages is to help the public **prepare in advance of a heat event**.

- **During heat event:** For release when a heat event is declared/occurring. The intent of these messages is to help the public **respond appropriately during a heat event**.

- **Post-heat event:** For release when a heat event is declared over or finished. The intent of these messages is to help the public **remain vigilant in the days following a heat event**, as temperatures remain elevated indoors/outdoors and latent health effects can persist.

For ease of your review and referencing, please open the list of proposed messages in a separate window or split screen mode. For each set of the proposed messages, please then consider the consultation questions posed in this form and indicate a response/rating for each. Where applicable, please provide any additional comments to support your response.

Consultation Questions - Importance

	Yes	Some	No
7. Are the proposed messages important for the ECCC weather warning system?	○	○	○
8. Are the proposed messages important at the time points indicated (pre-heat event, during, post-event)?	○	○	○

Additional comments: If you indicated ‘Some’ or ‘No’ to any of the questions above please explain.

There is a limit of 5,000 characters including spaces.

Consultation Questions - Action-Oriented

	Yes	Some	No
9. Are the proposed messages sufficiently action-oriented (e.g., would motivate behaviour change)?	○	○	○
10. Are the proposed messages appropriate to action at the time points indicated?	○	○	○

Additional comments: If you indicated ‘Some’ or ‘No’ to any of the questions above please explain.

There is a limit of 5,000 characters including spaces.

Consultation Questions - Evidence-Based

	Yes	Some	No
11. Are the proposed messages evidence-based?	○	○	○
12. Where applicable, do the proposed messages include the necessary conditional disclaimers needed (e.g., limitations for specific at-risk populations)?	○	○	○

Additional comments: If you indicated ‘Some’ or ‘No’ to any of the questions above please explain.

There is a limit of 5,000 characters including spaces.

Consultation Questions - Readable

	Yes	Some	No
13. Are the proposed messages written at a reading grade level appropriate for the general public (i.e., grade 6)?	○	○	○
14. Are the proposed messages free of jargon or complex terms?	○	○	○

Additional comments: If you indicated ‘Some’ or ‘No’ to any of the questions above please explain.

There is a limit of 5,000 characters including spaces.

Consultation Questions - Equitable

	Yes	Some	No
15. Are the proposed messages equitable?	○	○	○
16. Do the proposed messages provide heat-protective measures that are feasible for individuals of various socio-economic backgrounds?	○	○	○

Additional comments: If you indicated ‘Some’ or ‘No’ to any of the questions above please explain.

There is a limit of 5,000 characters including spaces.

Consultation Questions - Equitable (continued)

17. Which of the following groups should be listed as being at greater risk in the ECCC heat warnings?

	Yes	Unsure	No
Infants/babies and children	○	○	○
Older adults (over the age of 65 years)	○	○	○
People who regularly use drugs and alcohol	○	○	○
People who are overweight or obese	○	○	○
People who are pregnant or breastfeeding	○	○	○
People using certain prescription medications and nutritional supplements	○	○	○
People with developmental, behavioural, cognitive or mental health disorders, including dementia, depression, schizophrenia, Alzheimer’s disease	○	○	○
	Yes	Unsure	No
Those who have mobility impairments	○	○	○

	Yes	Unsure	No
People with chronic medical conditions, including heart disease, hypertension, kidney disease, metabolic conditions, neurological disease (Parkinson's disease), respiratory disease (breathing difficulties, chronic obstructive pulmonary disease, asthma), cancer	○	○	○
People who are acutely ill, including diarrhea, sunburn, fever or infection, dehydration	○	○	○
People who have a history of heat illness or previous heat stroke	○	○	○
Individuals who are experiencing homelessness or lack secure shelter	○	○	○
People living in crowded and/or understaffed, high-density housing with no indoor cooling such as congregate settings like shelters, long-term care, or other institutional housing	○	○	○
Indigenous peoples, including First Nations, Inuit, and Métis People participating in culturally important, land-based activities and ceremonies	○	○	○
	Yes	Unsure	No
People that lack access to transportation	○	○	○
People living on higher floors of multi-story buildings	○	○	○
Tourists and transient populations	○	○	○
Newcomers to Canada and those with language barriers	○	○	○
Racialized people	○	○	○

	Yes	Unsure	No
People experiencing a power outage due to other weather-related events	○	○	○
People living in rural or remote communities with reduced access to health and social supports	○	○	○

	Yes	Unsure	No
People living in large urban centres without/or with less access to green space	○	○	○
People experiencing material deprivation (low socioeconomic status)	○	○	○
People who are not regularly exposed to hot environments and lack proper acclimatization	○	○	○
People who live in buildings without air conditioning or with poor temperature control	○	○	○
People who are socially isolated or live alone	○	○	○
People who exercise strenuously outdoors in hot weather or indoors in areas that are poorly ventilated or do not have air-conditioning	○	○	○
People working outdoors or in environments where the industrial activity produces heat	○	○	○

	Yes	Unsure	No
People attending large outdoor gatherings with long exposure to heat	○	○	○
People who work outdoors or in confined spaces without air conditioning	○	○	○
People wearing personal protective equipment (PPE) in places not temperature controlled	○	○	○

	Yes	Unsure	No
Other (please specify in the comment box below)	◯	◯	◯

Consultation Questions - Applicable

	Yes	Some	No
18. Are the proposed messages applicable to your geographic region?	◯	◯	◯
19. Are the proposed messages appropriately reflective of various climate conditions in Canada (e.g., indoor/outdoor environments, extended events, more severe events, compounding events)?	◯	◯	◯

Additional comments: If you indicated ‘Some’ or ‘No’ to any of the questions above please explain.

There is a limit of 5,000 characters including spaces.

Additional Information (Optional)

Please provide any additional comments you feel should be considered for the update of Health Canada’s heat-health messages.

There is a limit of 5,000 characters including spaces.

Submit Message

Submit

If there are any changes you would like to make to your responses, please make them now before you click the "Submit" button below.

In order for your feedback to be considered, you must click “Submit".

Powered by Qualtrics

Français (Canada)

Intro

Introduction

Le Bureau des changements climatiques et de la santé (BCCS) de Santé Canada s’engage actuellement dans un projet décliné en plusieurs phases visant à réviser les messages sur la chaleur et la santé inclus dans les documents de communication de Santé Canada et diffusés au moyen du Système d’avertissement et d’intervention en cas de chaleur (SAIC) d’Environnement et Changement climatique Canada (ECCC). Sur la base d’un examen approfondi des données probantes réalisé par des chercheurs de l’Université d’Ottawa, le BCCS a élaboré une liste révisée de messages qui comprend des modifications aux énoncés existants, l’ajout de nouveaux messages, ainsi que des regroupements proposés sur la base du moment de la diffusion (avant l’événement de chaleur, pendant l’événement de chaleur et après l’événement de chaleur). Nous cherchons désormais à obtenir une rétroaction externe de la part d’experts et de chercheurs en santé publique de premier plan dans tout le Canada afin de nous assurer que les énoncés sont complets, orientés vers l’action, fondés sur des données probantes et reflètent des considérations comme la lisibilité, l’équité et l’applicabilité régionale.

Veuillez réaliser votre examen et votre évaluation avant 23 h 59 (HNE) le 20 août 2024. Si vous avez des questions, veuillez faire parvenir un courriel à Melissa Gorman (chercheure principale) à : melissa.gorman@hc-sc.gc.ca ou à Emily Tetzlaff (co-chercheure principale) à : emily.tetzlaff@hc-sc.gc.ca.

Remarque : Vous devez cliquer sur le bouton « Soumettre » à la fin du questionnaire pour que vos réponses soient enregistrées.

Modalités

Nous recueillons vos commentaires professionnels et vos idées afin d’obtenir votre avis au sujet des messages sur la chaleur et la santé de Santé Canada. Nous demandons certaines informations démographiques afin de nous assurer que nous représentons les opinions de divers types d’experts en la matière, y compris des experts en santé publique, en stress thermique, en santé environnementale, en changements climatiques, en équité en matière de santé ou dans d’autres disciplines connexes, et ce, afin de permettre une analyse pertinente relative à cette consultation. Santé Canada recueillera vos renseignements personnels par l’intermédiaire de l’outil Qualtrics et, à ce titre, est assujetti à la déclaration de confidentialité de Qualtrics. Les commentaires ou citations figurant dans le résumé de la consultation et dans les publications et/ou webinaires évalués par les pairs qui en résulteront ne seront pas attribués à une personne ou à une organisation en particulier.

Veuillez vous assurer que tous les commentaires écrits que vous fournissez sont de nature suffisamment générale pour que l'on ne puisse pas vous désigner comme étant leur auteur et qu'aucun nom ne soit divulgué.

Instructions pour réaliser la consultation

- Chaque question est facultative. Vous pouvez sauter n'importe quelle question, et ce, pour n'importe quelle raison.
- Si vous souhaitez revenir à une question précédente, vous pouvez le faire en cliquant sur le bouton vers la « page précédente ».
- Nous prévoyons que la consultation prendra environ d'une à deux heures. Vous ne devez pas nécessairement terminer la consultation en une seule session, vous pouvez reprendre la consultation là où vous l'avez laissée (avant la date de clôture) en utilisant le même ordinateur ou appareil et le même navigateur que ceux avec lesquels vous avez commencé la consultation.
- Vous devez cliquer sur le bouton « Soumettre » à la fin du questionnaire pour que vos réponses soient enregistrées.

DemoQs

Questions d'ordre démographique

1. Pour quel groupe/organisation travaillez-vous?

[ ]

2. Lequel des énoncés suivants décrit le mieux votre organisation? (Sélectionnez toutes les réponses qui s'appliquent.)

- ☐ Milieu universitaire/Recherche
- ☐ Autorité de santé publique régionale
- ☐ Autorité de santé publique provinciale ou territoriale
- ☐ Autorité de santé publique fédérale
- ☐ [ ] Autre, veuillez préciser :

3. Depuis combien de temps travaillez-vous dans ce domaine?

[ ]

4. Quel est votre niveau d'études le plus élevé?

- École primaire
- Diplôme d'enseignement secondaire (ou équivalent, par exemple formation générale)
- Certificat ou diplôme de l'enseignement supérieur
- Baccalauréat
- Maîtrise
- Doctorat
- Préfère ne pas divulguer
- [ ] Autre, veuillez préciser :

5. Veuillez indiquer de trois à cinq mots-clés qui décrivent le mieux votre domaine d'expertise (p. ex., physiologie de la chaleur, changements climatiques, santé environnementale, promotion de la santé, équité).

Questions d'ordre démographique (suite)

6. Dans quelle province ou dans quel territoire travaillez-vous? (Sélectionnez toutes les réponses qui s'appliquent.)

- [ ] Colombie-Britannique
- [ ] Alberta
- [ ] Saskatchewan
- [ ] Manitoba
- [ ] Ontario
- [ ] Québec
- [ ] Nouveau-Brunswick
- [ ] Terre-Neuve-et-Labrador
- [ ] Nouvelle-Écosse
- [ ] Île-du-Prince-Édouard
- [ ] Yukon
- [ ] Territoires du Nord-Ouest

- [ ] Nunavut
- [ ] Canada (fédéral)
- [ ] ______________ Autre, veuillez préciser :

ConsultQs

Renseignements généraux

Les alertes météorologiques sont des signaux utilisés pour sensibiliser les gens et/ou les inciter à se préparer à prendre des mesures. Au Canada, les alertes météorologiques sont diffusées par Environnement et Changement Climatique Canada (ECCC) pour les dangers météorologiques ou environnementaux, y compris la chaleur, qui sont soit en cours, soit imminents, soit prévus.

Le document accessible au moyen de ce lien fournit la liste des messages que Santé Canada propose d'utiliser dans le cadre du Système d'avertissement et d'intervention en cas de chaleur (SAIC) d'ECCC et qui seront diffusés au public pendant les alertes de chaleur en cours. En tant que tels, ces messages devraient comprendre les messages de protection de la santé les plus importants.

Les messages proposés sont classés en fonction de leur moment de diffusion, que ce soit avant, pendant ou après l'événement de chaleur :

- **Avant l'événement de chaleur** : Pour diffusion lorsqu'un événement de chaleur est anticipé (p. ex., 48 heures avant un événement de chaleur). L'objectif de ces messages est d'aider le public **à se préparer à un événement de chaleur**.
- **Pendant l'événement de chaleur** : Pour diffusion lorsqu'un événement de chaleur est déclaré ou se produit. L'objectif de ces messages est d'aider le public **à réagir de manière appropriée lors d'un événement de chaleur**.
- **Après l'événement de chaleur** : Pour diffusion lorsqu'un événement de chaleur est déclaré terminé. Ces messages ont pour but d'aider le public **à rester vigilant dans les jours qui suivent un événement de chaleur**, car les températures demeurent élevées à l'intérieur comme à l'extérieur et les effets latents sur la santé peuvent persister.

Pour faciliter votre examen et votre référencement, veuillez ouvrir la liste des messages proposés dans une fenêtre séparée ou encore en mode « écran partagé ». Pour chaque série de messages proposés, veuillez ensuite examiner les questions de consultation posées dans le présent formulaire et indiquer

une réponse ou votre évaluation pour chacune d'entre elles. Le cas échéant, veuillez fournir tout commentaire supplémentaire à l'appui de votre réponse.

Questions de la consultation - Importance

	Oui	Parfois	Non
7. Les messages proposés sont-ils importants pour le système d'alerte météorologique d'ECCC?	○	○	○
8. Les messages proposés sont-ils importants aux moments indiqués (avant, pendant et après l'événement)?	○	○	○

Commentaires supplémentaires : Si vous avez répondu « parfois » ou « non » à l'une des questions ci-dessus, veuillez expliquer.

Le nombre de caractères est limité à 5 000, espaces comprises.

Questions de la consultation - Pragmatisme

	Oui	Parfois	Non
9. Les messages proposés sont-ils suffisamment orientés vers l'action (p. ex., pour motiver un changement de comportement)?	○	○	○
10. Les messages proposés sont-ils appropriés pour agir aux moments indiqués?	○	○	○

Commentaires supplémentaires : Si vous avez répondu « parfois » ou « non » à l'une des questions ci-dessus, veuillez expliquer.

Le nombre de caractères est limité à 5 000, espaces comprises.

Questions de la consultation - Fondement sur des données probantes

	Oui	Parfois	Non
11. Les messages proposés sont-ils fondés sur des données probantes?	○	○	○
12. Le cas échéant, les messages proposés comprennent-ils les avertissements conditionnels nécessaires (p. ex., les limites pour des populations à risque particulières)?	○	○	○

Commentaires supplémentaires : Si vous avez répondu « parfois » ou « non » à l'une des questions ci-dessus, veuillez expliquer.

Le nombre de caractères est limité à 5 000, espaces comprises.

Questions de la consultation - Lisible

	Oui	Parfois	Non
13. Les messages proposés sont-ils rédigés à un niveau de lecture approprié pour le grand public (c'est-à-dire, la 6e année du primaire)?	○	○	○
14. Les messages proposés sont-ils exempts de jargon ou de termes complexes?	○	○	○

Commentaires supplémentaires : Si vous avez répondu « parfois » ou « non » à l'une des questions ci-dessus, veuillez expliquer.

Le nombre de caractères est limité à 5 000, espaces comprises.

Questions de la consultation - Équitable

	Oui	Parfois	Non
15. Les messages proposés sont-ils soucieux de l'équité?	○	○	○
16. Les messages proposés prévoient-ils des mesures de protection contre la chaleur réalisables, et ce, par des personnes de différents milieux socio-économiques?	○	○	○

Commentaires supplémentaires : Si vous avez répondu « parfois » ou « non » à l’une des questions ci-dessus, veuillez expliquer.

Le nombre de caractères est limité à 5 000, espaces comprises.

Questions de la consultation - Équitable (suite)

17. Parmi les groupes suivants, lequel devrait figurer sur la liste des groupes les plus exposés dans les alertes en cas de chaleur d’ECCC?

	Oui	Indécis	Non
Les nourrissons, bébés et enfants	○	○	○
Les personnes âgées (plus de 65 ans)	○	○	○
Les personnes qui consomment couramment des drogues et de l’alcool	○	○	○
Les personnes en surpoids ou obèses	○	○	○
Les personnes qui sont enceintes ou qui allaitent	○	○	○
Les personnes qui utilisent certains médicaments sur ordonnance et des compléments alimentaires	○	○	○
Les personnes souffrant de troubles du développement, du comportement, de la cognition ou de la santé mentale, y compris la démence, la dépression, la schizophrénie et la maladie d’Alzheimer	○	○	○
	Oui	Indécis	Non
Les personnes à mobilité réduite	○	○	○

	Oui	Indécis	Non
Les personnes souffrant de maladies chroniques, notamment de maladies cardiaques, d’hypertension, de maladies rénales, de maladies métaboliques, de maladies neurologiques (maladie de Parkinson), de maladies respiratoires (difficultés respiratoires, broncho-pneumopathie chronique obstructive, asthme), de cancer	○	○	○
Les personnes souffrant d’une maladie aiguë, notamment de diarrhée, de coups de soleil, de fièvre ou d’infection, de déshydratation	○	○	○
Les personnes qui ont des antécédents de maladie attribuable à la chaleur ou qui ont déjà subi un coup de chaleur	○	○	○
Les personnes sans domicile fixe ou qui ne disposent pas d’un abri sûr	○	○	○
Les personnes vivant dans des logements surpeuplés et/ou en sous-effectif, à forte densité et sans climatisation intérieure, comme les centres d’hébergement, les centres de soins de longue durée ou d’autres logements institutionnels	○	○	○
Les peuples autochtones, y compris les Premières Nations, les Inuits et les Métis, qui participent à des activités et à des cérémonies importantes sur le plan culturel et basées sur la terre	○	○	○

	Oui	Indécis	Non
Les personnes qui n’ont pas accès aux transports	○	○	○

	Oui	Indécis	Non
Les personnes vivant dans les étages supérieurs de bâtiments à plusieurs étages	○	○	○
Les touristes et les populations de passage	○	○	○
Les nouveaux arrivants au Canada et les personnes confrontées à des obstacles linguistiques	○	○	○
Les peuples racisés	○	○	○
Les personnes ayant subi une panne d'électricité en raison d'autres événements météorologiques	○	○	○
Les personnes vivant dans des communautés rurales ou isolées ayant un accès réduit aux services de santé ou aux services sociaux	○	○	○
	Oui	Indécis	Non
Les personnes vivant dans de grands centres urbains sans et/ou avec un accès réduit aux espaces verts	○	○	○
Les personnes en situation de privation matérielle (faible statut socio-économique)	○	○	○
Les personnes qui ne sont pas couramment exposées à des environnements chauds et qui manquent d'acclimatation	○	○	○
Les personnes qui vivent dans des bâtiments sans climatisation ou encore avec un mauvais système de contrôle de la température	○	○	○
Les personnes socialement isolées ou vivant seules	○	○	○
Les personnes qui font de l'exercice physique intense à l'extérieur par temps chaud ou à l'intérieur dans des endroits mal ventilés ou encore dépourvus de climatisation	○	○	○

	Oui	Indécis	Non
Les personnes travaillant à l'extérieur ou dans des environnements où l'activité industrielle produit de la chaleur	○	○	○

	Oui	Indécis	Non
Les personnes participant à de grands rassemblements en plein air avec une longue exposition à la chaleur	○	○	○
Les personnes qui travaillent à l'extérieur ou dans des espaces confinés sans climatisation	○	○	○
Les personnes portant des équipements de protection individuelle (EPI) dans des lieux où la température n'est pas contrôlée	○	○	○
Autre (veuillez préciser dans la boîte de commentaires ci-dessous) [ ]	○	○	○

Questions de la consultation - Applicable

	Oui	Parfois	Non
18. Les messages proposés sont-ils applicables à votre région géographique?	○	○	○
19. Les messages proposés reflètent-ils de manière appropriée les diverses conditions climatiques au Canada (p. ex., environnements intérieurs/extérieurs, événements prolongés, événements plus graves, événements aggravés)?	○	○	○

Commentaires supplémentaires : Si vous avez répondu « parfois » ou « non » à l'une des questions ci-dessus, veuillez expliquer.

Le nombre de caractères est limité à 5 000, espaces comprises.

Renseignements supplémentaires (section facultative)

Veuillez nous faire part de tout commentaire supplémentaire qui, selon vous, devrait être pris en compte pour la mise à jour des messages sur la chaleur et la santé de Santé Canada.

Le nombre de caractères est limité à 5 000, espaces comprises.

Submit Message

Si vous souhaitez modifier vos réponses, veuillez le faire avant de cliquer sur le bouton « Soumettre » ci-dessous.

Pour que vos commentaires soient pris en compte, vous devez cliquer sur le bouton « Soumettre ».

Powered by Qualtrics
