# Supplementary material for: Updating Health Canada’s Heat-Health Messages for the Environment and Climate Change Canada Heat Warning System: A Collaboration with Canadian Experts
Source: Int J Environ Res Public Health. 2025 Aug 13;22(8):1266. doi: 10.3390/ijerph22081266 (PMC12386431; doi:10.3390/ijerph22081266)
Supplement: Supplementary file 1 [file ijerph-22-01266-s001.zip › IJERPH_Supplementary Material File S6_Qualtrics Round 2.pdf]

English

Intro

Introduction

The Climate Change and Health Office (CCHO) at Health Canada is currently embarking on a multi-phased project to revise the heat-health messages included in Health Canada's communication materials and disseminated through the Environment and Climate Change Canada heat warning program. Informed by a comprehensive evidence review conducted by researchers at the University of Ottawa, CCHO has developed a revised list of messages which includes modifications to existing statements, the addition of new messages, as well as proposed groupings based on the timing of release (pre-heat event, during heat event and post-event). We are now seeking external feedback from leading public health experts and researchers across Canada to ensure the statements are comprehensive, action-oriented, evidence-based and reflect considerations such as readability, equity, and regional applicability.

Please complete your review and assessment by 11:59PM EST October 21st, 2024. If you have any questions please email Melissa Gorman (Principal Investigator) at melissa.gorman@hc-sc.gc.ca or Emily Tetzlaff (Co-Principal Investigator) at emily.tetzlaff@hc-sc.gc.ca.

Note: You must click the "Submit" button at the end of the questionnaire for your responses to be recorded.

Terms and Conditions

Your professional input and ideas are being collected in order to seek your feedback on Health Canada's heat-health messages. We request some demographic information in order to ensure we are representing the views of various types of subject matter experts, including experts in public health, heat stress, environmental health, climate change, health equity or other related disciplines to allow for meaningful analysis of this consultation. Health Canada will be collecting your information via the Qualtrics tool and, as such, is subject to Qualtrics' privacy statement. Comments or quotes featured in the consultation summary and any resulting peer-reviewed publications and/or webinars will not be attributed to any specific individual or organization.

Please ensure that any written comments you provide are sufficiently general that you cannot be identified as the author and that individual identities are not disclosed.

Instructions for Completing the Consultation

- Answering each question is optional. You may skip any question for any reason.
- If you would like to go back to a previous question, you may do so by clicking on the "Previous Page" button.
- We anticipate that the consultation review will take you approximately 1-2 hours. You do not have to complete the consultation in one session, you may return to continue where you left off (before

the closing date) by using the same computer or device and browser you began the consultation with.

- You must click the "Submit" button at the end of the questionnaire for your responses to be recorded.

DemoQs

Demographic Questions

1. Did you participate in the first round of consultation?

- ○ Yes
- ○ No

2. What group/organization do you work for?

[ ]

3. Which of the following best identifies your organization? (Select all that apply.)

- ☐ Academia/Research
- ☐ Regional Health Authority
- ☐ Provincial/Territorial Health Authority
- ☐ Federal Health Authority
- ☐ [ ] Other, please specify:

4. How long have you been employed in this field?

[ ]

5. What is your highest level of educational attainment?

- ○ Elementary School
- ○ Secondary School Diploma (or equivalent, e.g., GED)
- ○ College Certificate or Diploma
- ○ Bachelor's Degree
- ○ Master's Degree
- ○ Doctorate
- ○ Prefer Not To Disclose

- ○ [ ] Other, please specify:

6. Please provide 3 to 5 keywords which best describe your area of expertise (e.g., Heat Physiology, Climate Change, Environmental Health, Health Promotion, Equity).

[ ]

Demographic Questions (Continued)

7. Which province/territory do you work in? (Select all that apply.)

- ☐ British Columbia
- ☐ Alberta
- ☐ Saskatchewan
- ☐ Manitoba
- ☐ Ontario
- ☐ Quebec
- ☐ New Brunswick
- ☐ Newfoundland and Labrador
- ☐ Nova Scotia
- ☐ Prince Edward Island
- ☐ Yukon
- ☐ Northwest Territories
- ☐ Nunavut
- ☐ Canada (Federal)
- ☐ [ ] Other, please specify:

ConsultQs

Background Information

Weather alerts are signals to heighten awareness and initiate preparation for action. In Canada, Environment and Climate Change Canada (ECCC) issues weather alerts for weather or environmental hazard events, such as heat, that are either occurring, imminent, or forecast to develop. These weather alerts include health protective actions the public can take.

Health Canada is revising the heat-health messages shared through the ECCC warning system. These messages are released when active extreme heat events (heat waves) occur and, as such, should

include the most critical health-protective messages. These messages will be disseminated to the public at three-time points during an extreme heat event:

- **Pre-heat event:** For release when a heat event is anticipated (e.g. 24 to 48-hours in advance of a heat event). The intent of these messages is to help the public **prepare for the heat event**.
- **During heat event:** For release when a heat event is declared/occurring. The intent of these messages is to help the public **respond appropriately during a heat event**.
- **Post-heat event:** For release with extended heat warnings when a heat event is declared over or finished. The intent of these messages is to help the public **remain vigilant immediately following a heat event**, as temperatures remain elevated indoors/outdoors and latent health effects can persist.

The following image provides an example of how the heat-health statements are included within a Heat Warning. Click here for additional examples.

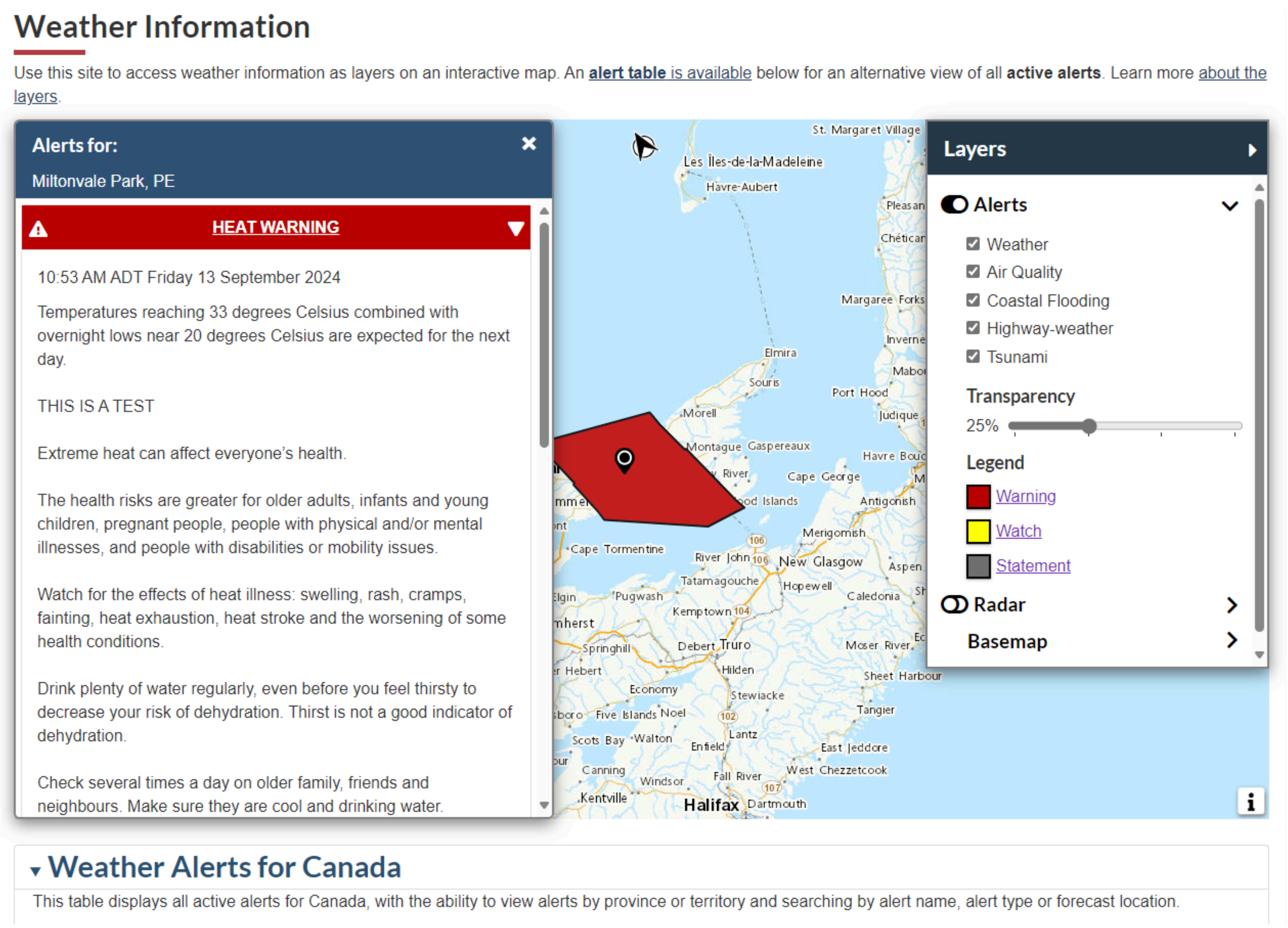

Following the first round of consultation, we have applied changes to the proposed messages. For each of the revised messages, please consider the modifications made and respond to the

consultation questions posed in this form. Where applicable, please provide any additional comments to support your response.

ECCC Heat Warning System Statements: Heat Impacts

	**Released with Early Warnings** (24-48 hours in advance of a heat event)	**Released with Heat Alert** (during heat event)	**Released when Event Ends** (when alert ends)
**Original** (Round 1)	Extreme heat can affect everyone's health. Prepare to take action to reduce your risk. Heat-related risks are greater for specific groups.	Extreme heat can affect everyone's health. Take action to reduce your risk. Heat-related risks are greater for specific groups.	Extreme heat can affect everyone's health. Continue to take precautions to reduce your risk. Heat-related risks are greater for specific groups.
**Revised** (Round 2)	Prepare to reduce your risk - extreme heat can affect everyone's health. Determine if you or your family are at greater risk of heat illness.	Take action to protect yourself and others - extreme heat can affect everyone's health. Determine if you or your family are at greater risk of heat illness.	Continue to take precautions to reduce your risk. Heat illness may develop after the heat event is over so continue to monitor yourself and others.

Consultation Questions - Heat Impact

	Yes	No	I don't know
8. Do you agree with the modifications proposed for these statements?	○	○	○
9. Should these statements be included in the ECCC weather warning system?	○	○	○

Additional comments: Please explain if you indicated 'No' or 'I don't know' to any of the questions above and provide any additional comments you feel necessary.

There is a limit of 5,000 characters including spaces.

ECCC Heat Warning System Statements: Heat-Related Illness and First Aid

	**Released with Early Warnings**	**Released with Heat Alert**	**Released when Event Ends**

	(24-48 hours in advance of a heat event)	(during heat event)	(when alert ends)
**Original** (Round 1)	Heat can cause dehydration and heat-related illness, including swelling, rash, cramps, fainting, heat exhaustion, and the worsening of pre-existing health conditions. Watch for the early signs of heat illness as these can evolve into life-threatening emergencies.	Watch for the early signs of heat exhaustion - headache, nausea, dizziness, thirst, dark urine. Stop your activity, and rest. Move to a cool place. If alone, notify a family member, neighbour or friend. Remove all unnecessary clothing. Apply cool compresses or damp cloths on your skin. Drink water to replace fluids. If symptoms do not subside, call 911.	The signs and symptoms of heat can continue to develop even after an extreme heat event is declared over. Continue to monitor yourself and others.
**Revised** (Round 2)	Watch for the early signs of heat illness in yourself and others as these can evolve into life-threatening emergencies. Heat can cause dehydration and heat illness, including swelling, rash, cramps, fainting, heat exhaustion, and worsening of pre-existing health conditions.	Watch for the early signs of heat illness in yourself and others which may include headache, nausea, dizziness, thirst, dark urine and intense fatigue. Stop your activity, and rest. Move to a cool place. If alone, call a family member, neighbour or friend. Remove all extra clothing. Apply cool compresses or damp cloths on your skin. Drink water to replace fluids. If symptoms do not go away, call 911.	Continue to monitor yourself and others for signs of feeling unwell or heat illness. Even though a heat warning has ended, heat illness can happen after the event.

Consultation Questions - Heat-Related Illness and First Aid

	Yes	No	I don't know
10. Do you agree with the modifications proposed for these statements?	○	○	○
11. Should these statements be included in the ECCC weather warning system?	○	○	○

Additional comments: Please explain if you indicated ‘No’ or ‘I don’t know’ to any of the questions above and provide any additional comments you feel necessary.

There is a limit of 5,000 characters including spaces.

ECCC Heat Warning System Statements: Heat Emergency

	**Released with Early Warnings** (24-48 hours in advance of a heat event)	**Released with Heat Alert** (during heat event)	**Released when Event Ends** (when alert ends)
**Original** (Round 1)	Heat stroke is a medical emergency - Call 911 (or your local emergency number) immediately if heat stroke is suspected.	If you, or someone around you, has stopped sweating, has red, hot, dry skin, dizziness, confusion, nausea, extreme thirst - seek immediate medical attention. Try and cool the person right away by moving them to a cool place, removing excess clothing, applying cold water or ice packs around the body, and fanning.	Pay close attention to how you, and those around you, feel. The effects of heat can continue to be experienced even after an extreme heat event is declared over. Continue to watch for the effects of heat illness.
**Revised** (Round 2)	Call 911 immediately if heat stroke is suspected. Heat stroke is a medical emergency.	Call 911 if you, or someone around you, is feeling unwell or showing signs of heat stroke which can include red, hot skin, dizziness, diarrhea, confusion, change in consciousness, nausea, extreme thirst, change in sweating. While you wait for medical attention, try to cool the person by moving them to a cool place, removing extra clothing, applying cold water or ice packs around the body, and fanning.	Pay close attention to how you, and those around you feel. The effects of heat can continue to be experienced even after an extreme heat event is declared over. Continue to watch for the effects of heat illness.

Consultation Questions - Heat Emergency

	Yes	No	I don't know
12. Do you agree with the modifications proposed for these statements?	○	○	○
13. Should these statements be included in the ECCC weather warning system?	○	○	○

Additional comments: Please explain if you indicated ‘No’ or ‘I don’t know’ to any of the questions above and provide any additional comments you feel necessary.

There is a limit of 5,000 characters including spaces.

ECCC Heat Warning System Statements: Check-Ins

	**Released with Early Warnings** (24-48 hours in advance of a heat event)	**Released with Heat Alert** (during heat event)	**Released when Event Ends** (when alert ends)
**Original** (Round 1)	Create a plan to arrange for regular visits and support your family, friends and community during high heat days.	Check-in regularly with older, and otherwise vulnerable people in-person and/or on the phone multiple times a day, especially in the evening when indoor temperatures are high.	Continue checking in on older, and otherwise vulnerable people, for a few days as indoor temperatures can remain high.
**Revised** (Round 2)	Talk to family, friends and neighbours to see how they are preparing for the heat. Create a plan for regular visits and ways to support each other, especially those at greater risk.	Check-on older adults, and otherwise at-risk people (children, pregnant people, those with mobility challenges) in-person and/or on the phone multiple times a day.	Continue checking in on older, and otherwise at-risk people, for a few days as temperatures can remain high indoors.

Consultation Questions - Check-Ins

	Yes	No	I don't know
14. Do you agree with the modifications proposed for these statements?	○	○	○
15. Should these statements be included in the ECCC weather warning system?	○	○	○

Additional comments: Please explain if you indicated ‘No’ or ‘I don’t know’ to any of the questions above and provide any additional comments you feel necessary.

There is a limit of 5,000 characters including spaces.

ECCC Heat Warning System Statements: Hydration

	**Released with Early Warnings** (24-48 hours in advance of a heat event)	**Released with Heat Alert** (during heat event)	**Released when Event Ends** (when alert ends)
**Original** (Round 1)	As the temperature begins to rise, ensure you are drinking water frequently to avoid becoming dehydrated, which can lead to a heat-related illness.	Exposure to heat, especially when you are physically active will cause your body to lose fluids through sweat. Drink water even before you feel thirsty to replace those fluids.	If you experienced dehydration or a heat-related illness during the heat event, consult with your health care provider to prepare for future events.
**Revised** (Round 2)	Drink water often to avoid becoming dehydrated, which can lead to a heat illness.	Exposure to heat will cause your body to lose fluids through sweat. Drink water often and before you feel thirsty to replace fluids.	N/A

Consultation Questions - Hydration

	Yes	No	I don't know
16. Do you agree with the modifications proposed for these statements?	○	○	○
17. Should these statements be included in the ECCC weather warning system?	○	○	○

Additional comments: Please explain if you indicated ‘No’ or ‘I don’t know’ to any of the questions above and provide any additional comments you feel necessary.

There is a limit of 5,000 characters including spaces.

ECCC Heat Warning System Statements: Reducing Risk in the Home

	**Released with Early Warnings**<br>(24-48 hours in advance of a heat event)	**Released with Heat Alert**<br>(during heat event)	**Released when Event Ends**<br>(when alert ends)
**Original** (Round 1)	Before the temperatures rise, check-in on your home cooling strategies, including air-conditioning, and ensuring windows are operable.<br><br>Before the temperatures rise, check-in on your home cooling strategies, including fans.	Turn on your air conditioning if available or move to a cooler area of the home (e.g., basement), close curtains, blinds, or shades and/or open windows to create a cross-breeze.<br><br>Use a fan to help you stay cool and aim the air flow in your direction. Note: Fans are ineffective at very high temperatures, especially for older adults.	Interior temperatures can remain high even after a heat event is over. Continue to monitor your homes temperature and apply cooling strategies as needed.<br><br>As the temperature lowers, use a fan to circulate cool air into your home.
**Revised** (Round 2)	Find ways to keep your living space cool and make sure air-conditioning, fans, and windows are working.	Turn on air conditioning or move to a cooler area of your living space. Close curtains, blinds, or shades, open windows to create a cross-breeze. Use a fan to help you stay cool. Note: Fans are ineffective at very high temperatures (35° C) – always use in combination with other ways to stay cool.	Keep checking the temperature of your living space and stay cool by opening windows and using a fan to move cool air into your living space.

Consultation Questions - Reducing Risk in the Home

	Yes	No	I don't know
18. Do you agree with the modifications proposed for these statements?	○	○	○
19. Should these statements be included in the ECCC weather warning system?	○	○	○

Additional comments: Please explain if you indicated ‘No’ or ‘I don’t know’ to any of the questions above and provide any additional comments you feel necessary.

There is a limit of 5,000 characters including spaces.

ECCC Heat Warning System Statements: Reducing Risk Outdoors

	**Released with Early Warnings** (24-48 hours in advance of a heat event)	**Released with Heat Alert** (during heat event)	**Released when Event Ends** (when alert ends)
**Original** (Round 1)	Plan ahead and schedule outdoor activities during the coolest parts of the day or reschedule until the heat event has passed.	Limit direct exposure to the sun and heat. Wear lightweight, light-coloured, loose-fitting clothing, a wide brimmed hat, or umbrella to reduce your risk of sunburn and overheating.	Continue being cautious when heading outdoors as temperatures remain elevated.
**Revised** (Round 2)	Plan and schedule outdoor activities during the coolest parts of the day or reschedule until the heat event has passed. If outdoors, seek shaded areas.	Limit direct exposure to the sun and heat. Wear lightweight, light-coloured, loose-fitting clothing, a wide brimmed hat and apply sunblock to reduce your risk of sunburn and overheating.	Continue being careful outdoors as temperatures remain high.

Consultation Questions - Reducing Risk Outdoors

	Yes	No	I don't know
20. Do you agree with the modifications proposed for these statements?	○	○	○
21. Should these statements be included in the ECCC weather warning system?	○	○	○

Additional comments: Please explain if you indicated 'No' or 'I don't know' to any of the questions above and provide any additional comments you feel necessary.

There is a limit of 5,000 characters including spaces.

ECCC Heat Warning System Statements: Nighttime

	**Released with Early Warnings** (24-48 hours in advance of a heat event)	**Released with Heat Alert** (during heat event)	**Released when Event Ends** (when alert ends)
**Original** (Round 1)	N/A	N/A	N/A
**Revised** (Round 2)	Plan to ensure you have a cool space to sleep.	Sleep in the coolest part of your living space and open windows, if safe. Take a cool shower before bed and wear light and loose-fitting clothes.	N/A

Consultation Questions - Nighttime

	Yes	No	I don't know
22. Do you agree with the modifications proposed for these statements?	○	○	○
23. Should these statements be included in the ECCC weather warning system?	○	○	○

Additional comments: Please explain if you indicated ‘No’ or ‘I don’t know’ to any of the questions above and provide any additional comments you feel necessary.

There is a limit of 5,000 characters including spaces.

ECCC Heat Warning System Statements: Cooling Spaces

	**Released with Early Warnings** (24-48 hours in advance of a heat event)	**Released with Heat Alert** (during heat event)	**Released when Event Ends** (when alert ends)
**Original** (Round	If you do not have home cooling strategies	If you find yourself in an overheated home,	Interior temperatures can remain high even

1)	available, identify air-conditioned spots close by in your community where you can cool off (e.g., community center, library, mall).	relocate to a cool public space in your community.	after a heat event is over. Continue to monitor your homes temperature and apply cooling strategies as needed.
**Revised** (Round 2)	Identify air-conditioned or cool spots in your community where you can go (e.g., community center, library, stores, shaded parks). Plan for transport assistance if needed.	Check your thermostat or thermometer. Move to a cool public space such as a community centre, swimming pool, splash park or lakes if your living space is hot.	Continue to monitor the temperature of your living space and keep cool as needed. Temperatures can remain high even after a heat event is over.

Consultation Questions - Cooling Spaces

	Yes	No	I don't know
24. Do you agree with the modifications proposed for these statements?	○	○	○
25. Should these statements be included in the ECCC weather warning system?	○	○	○

Additional comments: Please explain if you indicated 'No' or 'I don't know' to any of the questions above and provide any additional comments you feel necessary.

There is a limit of 5,000 characters including spaces.

ECCC Heat Warning System Statements: Car Safety

	**Released with Early Warnings** (24-48 hours in advance of a heat event)	**Released with Heat Alert** (during heat event)	**Released when Event Ends** (when alert ends)
**Original** (Round 1)	Never leave people or pets inside a parked vehicle when it is hot outdoors. Look twice before locking and leaving.	Never leave people or pets inside a parked vehicle when it is hot outdoors. Look twice before locking and leaving.	Never leave people or pets inside a parked vehicle when it is hot outdoors. Look twice before locking and leaving.
**Revised** (Round 2)	Never leave people or pets inside a parked vehicle. Check the vehicle before locking to	Never leave people or pets inside a parked vehicle. Check the vehicle before locking to	Never leave people or pets inside a parked vehicle. Check the vehicle before locking to

	make sure no one is left behind.	make sure no one is left behind.	make sure no one is left behind.

Consultation Questions - Car Safety

	Yes	No	I don't know
26. Do you agree with the modifications proposed for these statements?	○	○	○
27. Should these statements be included in the ECCC weather warning system?	○	○	○

Additional comments: Please explain if you indicated 'No' or 'I don't know' to any of the questions above and provide any additional comments you feel necessary.

There is a limit of 5,000 characters including spaces.

ECCC Heat Warning System Statements: Information and Resources

	**Released with Early Warnings** (24-48 hours in advance of a heat event)	**Released with Heat Alert** (during heat event)	**Released when Event Ends** (when alert ends)
**Original** (Round 1)	Be aware of local community resources, health and emergency authorities and prepare a list of contact numbers or web links where you can get help.	Watch for heat warnings and follow the recommendations of Environment Canada or your region's public health authority.	If your home is still hot, contact your municipality to locate cooling centers available near you and for tips and services available in the community to beat the heat.
**Revised** (Round 2)	Be aware of local community resources, and health and emergency authorities that can help you stay cool and safe from the heat.	Monitor current heat alerts via the Public Weather Alerts website, or the WeatherCAN app. Follow the recommendations of your region's public health authority.	Keep helpful contacts and heat-health web links to be prepared for the next heat event.

Consultation Questions - Information and Resources

	Yes	No	I don't know
28. Do you agree with the modifications proposed for these statements?	○	○	○
29. Should these statements be included in the ECCC weather warning system?	○	○	○

Additional comments: Please explain if you indicated 'No' or 'I don't know' to any of the questions above and provide any additional comments you feel necessary.

There is a limit of 5,000 characters including spaces.

ECCC Heat Warning System Statements: Medical Consultation

	**Released with Early Warnings** (24-48 hours in advance of a heat event)	**Released with Heat Alert** (during heat event)	**Released when Event Ends** (when alert ends)
**Original** (Round 1)	Before the onset of warmer weather, ask a health professional (i.e., Pharmacist) how medications or health conditions can affect your risk in the sun and heat.	Continue taking your medication as prescribed. You should not modify how you take your medication unless you have first consulted with your doctor or pharmacist.	If you experienced a heat-related illness or felt unwell due to the heat, consult with your health care provider to prepare for future events.
**Revised** (Round 2)	Check if any of your medication or health conditions put you at greater risk in the heat. Talk to a healthcare provider for further guidance.	Continue taking your medication as prescribed, unless your healthcare provider tells you otherwise.	Ask a healthcare provider how you can reduce your risk for the next heat event.

Consultation Questions - Medical Consultation

	Yes	No	I don't know
30. Do you agree with the modifications proposed for these statements?	○	○	○
31. Should these statements be included in the ECCC weather warning system?	○	○	○

Additional comments: Please explain if you indicated 'No' or 'I don't know' to any of the questions above and provide any additional comments you feel necessary.

There is a limit of 5,000 characters including spaces.

ECCC Heat Warning System Statements: Workers at Risk

	**Released with Early Warnings** (24-48 hours in advance of a heat event)	**Released with Heat Alert** (during heat event)	**Released when Event Ends** (when alert ends)
**Original** (Round 1)	Individuals exposed to heat at work (outdoors and indoors) should consult with their workplace to prepare in advance of the coming heat season.	Outdoor and indoor workers should take scheduled breaks in a shaded or cooler space with good ventilation (air flow) while seated or reclined. Remove excess protective gear and continue to hydrate.	Outdoor and indoor workers should continue to apply caution and apply measures to reduce their risk of heat stress as temperatures remain elevated.
**Revised** (Round 2)	Talk to your workplace to prepare before the extreme heat.	Take planned breaks in a shaded or cooler space with good air flow. Remove extra protective gear (if safe) and keep drinking water. When working outdoors, limit direct exposure to the sun and heat. Wear light-weight, light-coloured clothing.	Continue to be careful and take actions to reduce your risk of heat illnesses as temperatures may remain high at your workplace.

Consultation Questions - Workers at Risk

	Yes	No	I don't know
32. Do you agree with the modifications proposed for these statements?	○	○	○
33. Should these statements be included in the ECCC weather warning system?	○	○	○

Additional comments: Please explain if you indicated 'No' or 'I don't know' to any of the questions above and provide any additional comments you feel necessary.

There is a limit of 5,000 characters including spaces.

ECCC Heat Warning System Statements: Cooking

	**Released with Early Warnings** (24-48 hours in advance of a heat event)	**Released with Heat Alert** (during heat event)	**Released when Event Ends** (when alert ends)
**Original** (Round 1)	Plan ahead to ensure your meals don't require the use of the oven or other heat-generating appliances to limit additional indoor heat.	Avoid meals that require the use of the oven and other heat-generating appliances to limit additional indoor heat.	Continue to prepare meals that don't require the use of the oven or other heat-generating appliances to limit additional indoor heat.
**Revised** (Round 2)	Plan to ensure your meals don't require the use of the oven or stove to limit additional heat in your living space.	Plan meals that don't require the oven or stove, which make more heat. This helps keep your living space cooler.	Continue to prepare meals that don't require the use of the oven or stove to keep your living space cooler.

Consultation Questions - Cooking

	Yes	No	I don't know
34. Do you agree with the modifications proposed for these statements?	○	○	○
35. Should these statements be included in the ECCC weather warning system?	○	○	○

Additional comments: Please explain if you indicated 'No' or 'I don't know' to any of the questions above and provide any additional comments you feel necessary.

There is a limit of 5,000 characters including spaces.

ECCC Heat Warning System Statements: Extreme Heat and Special Air Quality Statements or Air Quality Advisories

	**Released with Early Warnings** (24-48 hours in advance of a heat event)	**Released with Heat Alert** (during heat event)	**Released when Event Ends** (when alert ends)
**Original** (Round 1)	N/A	N/A	N/A
**Revised** (Round 2)	Plan to check the Air Quality Health Index value in your region and reduce your exposure to wildfire smoke.	When there is an extreme heat event occurring with wildfire smoke, prioritize keeping cool.	If air quality has improved, open windows and doors to move cool air into the space at night, if safe.

Consultation Questions - Extreme Heat and Special Air Quality Statements or Air Quality Advisories

	Yes	No	I don't know
36. Do you agree with the modifications proposed for these statements?	○	○	○
37. Should these statements be included in the ECCC weather warning system?	○	○	○

Additional comments: Please explain if you indicated ‘No’ or ‘I don’t know’ to any of the questions above and provide any additional comments you feel necessary.

There is a limit of 5,000 characters including spaces.

Consultation Questions

This consultation process aims to ensure the statements are comprehensive, action-oriented, evidence-based, and consider readability, equity, and regional applicability. Considering all statements together, please respond to the questions posed below:

	Yes	Some	No
38. Are the proposed messages important for the ECCC weather warning system?	○	○	○
39. Are the proposed messages important at the time points indicated (pre-heat event, during, post-event)?	○	○	○
40. Are the proposed messages sufficiently action-oriented (e.g., would motivate behaviour change)?	○	○	○

	Yes	Some	No
41. Are the proposed messages appropriate to action at the time points indicated?	○	○	○

	Yes	Some	No
42. Are the proposed messages evidence-based?	○	○	○
43. Where applicable, do the proposed messages include the necessary conditional disclaimers needed (e.g., limitations for specific at-risk populations)?	○	○	○
44. Are the proposed messages written at a reading grade level appropriate for the general public (i.e., grade 6)?	○	○	○
45. Are the proposed messages free of jargon or complex terms?	○	○	○

	Yes	Some	No
46. Are the proposed messages equitable?	○	○	○
47. Do the proposed messages provide heat-protective measures that are feasible for individuals of various socio-economic backgrounds	○	○	○
48. Are the proposed messages applicable to your geographic region?	○	○	○
49. Are the proposed messages appropriately reflective of various climate conditions in Canada (e.g., indoor/outdoor environments, extended events, more severe events, compounding events)?	○	○	○

Ranking

The leading meteorologist may select only a few messages during a given heat event. Please rank the statements from 1 to 15, with 1 representing the highest priority for inclusion within a heat warning, and 15 representing the least important.

[ ] Take action to protect yourself and others - extreme heat can affect everyone's health. Determine if you or your family are at greater risk of heat illness.

[ ] Watch for the early signs of heat illness in yourself and others which may include headache, nausea, dizziness, thirst, dark urine and intense fatigue. Stop your activity, and rest. Move to a cool place. If alone, call a family member, neighbour or friend. Remove all extra clothing. Apply cool compresses or damp cloths on your skin. Drink water to replace fluids. If symptoms do not go away, call 911.

[ ] Call 911 if you, or someone around you, is feeling unwell or showing signs of heat stroke which can include red, hot skin, dizziness, diarrhea, confusion, change in consciousness, nausea, extreme thirst, change in sweating. While you wait for medical attention, try to cool the person by moving them to a cool place, removing extra clothing, applying cold water or ice packs around the body, and fanning.

[ ] Check on older adults and other at-risk people (children, pregnant people, those with mobility challenges) in-person and/or on the phone multiple times a day.

[ ] Exposure to heat will cause your body to lose fluids through sweat. Drink water often and before you feel thirsty to replace fluids.

[ ] Turn on air conditioning or move to a cooler area of your living space. Close curtains, blinds, or shades, open windows to create a cross-breeze. Use a fan to help you stay cool. Note: Fans are ineffective at cooling the body at very high temperatures (35°C) – always use in combination with other ways to stay cool.

[ ] Limit direct exposure to the sun and heat. Wear lightweight, light-coloured, loose-fitting clothing, a wide brimmed hat and apply sunblock to reduce your risk of sunburn and overheating.

[ ] Sleep in the coolest part of your living space and open windows, if safe. Take a cool shower before bed and wear light and loose-fitting clothes.

[ ] Check your thermostat or thermometer. Move to a cool public space such as a community centre, swimming pool, splash park or lakes if your living space is hot.

[ ] Never leave people or pets inside a parked vehicle. Check the vehicle before locking to make sure no one is left behind.

[ ] Monitor current heat alerts via the Public Weather Alerts website or the WeatherCAN app. Follow the recommendations of your region's public health authority.

[ ] Continue taking your medication as prescribed, unless your healthcare provider tells you otherwise.

[ ] Take planned breaks in a shaded or cooler space with good air flow. Remove extra protective gear (if safe) and keep drinking water. When working outdoors, limit direct exposure to the sun and heat. Wear light-weight, light-coloured clothing.

[ ] Plan meals that don't require the oven or stove, which make more heat. This helps keep your living space cooler.

[ ] When there is an extreme heat event occurring with wildfire smoke, prioritize keeping cool.

Additional Information (Optional)

Please provide any additional comments you feel should be considered for the update of Health Canada's heat-health messages.

There is a limit of 5,000 characters including spaces.

Submit Message

Submit

If there are any changes you would like to make to your responses, please make them now before you click the "Submit" button below.

In order for your feedback to be considered, you must click “Submit".

Powered by Qualtrics

Français (Canada)

Intro

Introduction

Le Bureau des changements climatiques et de la santé (BCCS) de Santé Canada s'engage actuellement dans un projet à phases multiples visant à réviser les messages sur la chaleur et la santé qui sont compris dans le matériel de communication de Santé Canada et qui sont diffusés dans le cadre du programme d'avertissement de chaleur d'Environnement et Changement climatique Canada. Sur la base d'un examen approfondi des données probantes réalisé par des chercheurs de l'Université d'Ottawa, le BCCS a élaboré une liste révisée de messages qui comprend des modifications aux énoncés existants, l'ajout de nouveaux messages, ainsi que des regroupements proposés sur la base du moment de la diffusion (avant l'événement de chaleur, pendant l'événement de chaleur et après l'événement de chaleur). Nous souhaitons maintenant obtenir une rétroaction externe de la part d'experts et de chercheurs en santé publique de premier plan dans tout le Canada afin de nous assurer que les énoncés sont complets, qu'ils sont orientés vers l'action et fondés sur des données probantes et qu'ils reflètent des considérations telles que la lisibilité, l'équité et l'applicabilité régionale.

Veuillez terminer votre révision et votre évaluation avant **23 h 59 (HNE), le 21 octobre 2024**.
Si vous avez des questions, veuillez faire parvenir un courriel à Melissa Gorman (chercheure principale) à : melissa.gorman@hc-sc.gc.ca ou à Emily Tetzlaff (co-chercheure principale) à : emily.tetzlaff@hc-sc.gc.ca.

Remarque : Vous devez cliquer sur le bouton « Soumettre » à la fin du questionnaire pour que vos réponses soient enregistrées.

Modalités

Nous recueillons vos commentaires professionnels et vos idées afin d'obtenir votre avis au sujet des messages sur la chaleur et la santé de Santé Canada. Nous demandons certains renseignements démographiques afin de nous assurer que nous représentons les opinions de divers types d'experts en la matière, y compris des experts en santé publique, en stress thermique, en santé environnementale, en changements climatiques, en équité en matière de santé ou d'autres disciplines connexes, et ce, afin de permettre une analyse pertinente de cette consultation. Santé Canada recueillera vos renseignements à l'aide de l'outil de Qualtrics et, à ce titre, est assujetti à la déclaration de confidentialité de Qualtrics (*Privacy Statement* – en anglais seulement). Les commentaires ou citations figurant dans le résumé de la consultation ainsi que dans les publications et/ou webinaires évalués par les pairs qui en résulteront ne seront pas attribués à une personne ou à une organisation en particulier.

Veuillez vous assurer que tous les commentaires écrits que vous fournissez sont de nature suffisamment générale pour que l'on ne puisse pas vous désigner comme étant leur auteur et qu'aucun nom n'est divulgué.

Instructions pour réaliser la consultation

- Chaque question est facultative. Vous pouvez sauter n'importe quelle question, et ce, pour n'importe quelle raison.
- Si vous souhaitez revenir à une question précédente, vous pouvez le faire en cliquant sur le bouton « page précédente ».
- Nous prévoyons que la consultation prendra environ d'une à deux heures. Il n'est pas nécessaire que vous terminiez la consultation en une seule session; vous pouvez reprendre la consultation là où vous l'avez laissée (avant la date de clôture) en utilisant le même ordinateur ou appareil et le même navigateur que ceux avec lesquels vous avez commencé la consultation.
- Vous devez cliquer sur le bouton « Soumettre » à la fin du questionnaire pour que vos réponses soient enregistrées.

DemoQs

Questions d'ordre démographique

1. Avez-vous participé à la première ronde de consultations?

○ Oui

○ Non

2. Pour quel groupe/quelle organisation travaillez-vous?

[ ]

3. Lequel des énoncés suivants décrit le mieux votre organisation? (Sélectionnez toutes les réponses qui s'appliquent.)

☐ Milieu universitaire/Recherche

☐ Autorité de santé publique régionale

☐ Autorité de santé publique provinciale ou territoriale

☐ Autorité de santé publique fédérale

☐ [ ] Autre, veuillez préciser :

4. Depuis combien de temps travaillez-vous dans ce domaine?

5. Quel est votre niveau d'études le plus élevé?

- École primaire
- Diplôme d'enseignement secondaire (ou équivalent, par exemple formation générale)
- Certificat ou diplôme de l'enseignement supérieur
- Baccalauréat
- Maîtrise
- Doctorat
- Préfère ne pas divulguer
- Autre, veuillez préciser :

6. Veuillez indiquer de trois à cinq mots-clés qui décrivent le mieux votre domaine d'expertise (p. ex., physiologie de la chaleur, changements climatiques, santé environnementale, promotion de la santé, équité).

Questions d'ordre démographique (suite)

7. Dans quelle province ou dans quel territoire travaillez-vous? (Sélectionnez toutes les réponses qui s'appliquent.)

- Colombie-Britannique
- Alberta
- Saskatchewan
- Manitoba
- Ontario
- Québec
- Nouveau-Brunswick
- Terre-Neuve-et-Labrador
- Nouvelle-Écosse

☐ Île-du-Prince-Édouard

☐ Yukon

☐ Territoires du Nord-Ouest

☐ Nunavut

☐ Canada (fédéral)

☐ [ ] Autre, veuillez préciser :

ConsultQs

Renseignements généraux

Les alertes météorologiques sont des signaux utilisés pour sensibiliser les gens et les inciter à se préparer à prendre des mesures. Au Canada, les alertes météorologiques sont diffusées par Environnement et Changement Climatique Canada (ECCC) lors de dangers d'ordre météorologique ou environnemental, comme la chaleur, qui sont en cours ou qui sont imminents ou prévus. Ces alertes météorologiques font notamment mention de mesures de protection de la santé que le public peut prendre.

Santé Canada procède actuellement à une révision des messages sur la chaleur et la santé qui sont diffusés par l'intermédiaire du système d'avertissement d'ECCC. Ces messages sont diffusés lors d'événements de chaleur extrême (canicules) et, à ce titre, ils devraient comprendre les messages les plus importants en matière de protection de la santé. Ces messages seront communiqués au public à trois différents stades durant un événement de chaleur extrême :

- **Alerte précoce (avant l'événement de chaleur)** : message à diffuser lorsqu'un événement de chaleur est prévu (p. ex. de 24 à 48 heures avant un événement de chaleur). Ces messages ont pour but d'aider le public **à se préparer à un événement de chaleur**.
- **Alerte de chaleur (pendant l'événement de chaleur)** : message à diffuser lorsqu'un événement de chaleur est déclaré ou se produit. Ces messages ont pour but d'aider le public **à réagir de manière appropriée lors d'un événement de chaleur**.
- **Après l'événement de chaleur** : message à diffuser lors d'avertissements de chaleur prolongés, lorsque l'événement de chaleur est déclaré terminé. Ces messages ont pour but d'aider le public **à rester vigilant immédiatement après un événement de chaleur**, car les températures demeurent élevées à l'intérieur comme à l'extérieur et que les effets latents sur la santé peuvent persister.

L'image qui suit présente un exemple de messages sur la chaleur et la santé qui sont diffusés lors d'un avertissement de chaleur. Cliquez ici pour d'autres exemples.

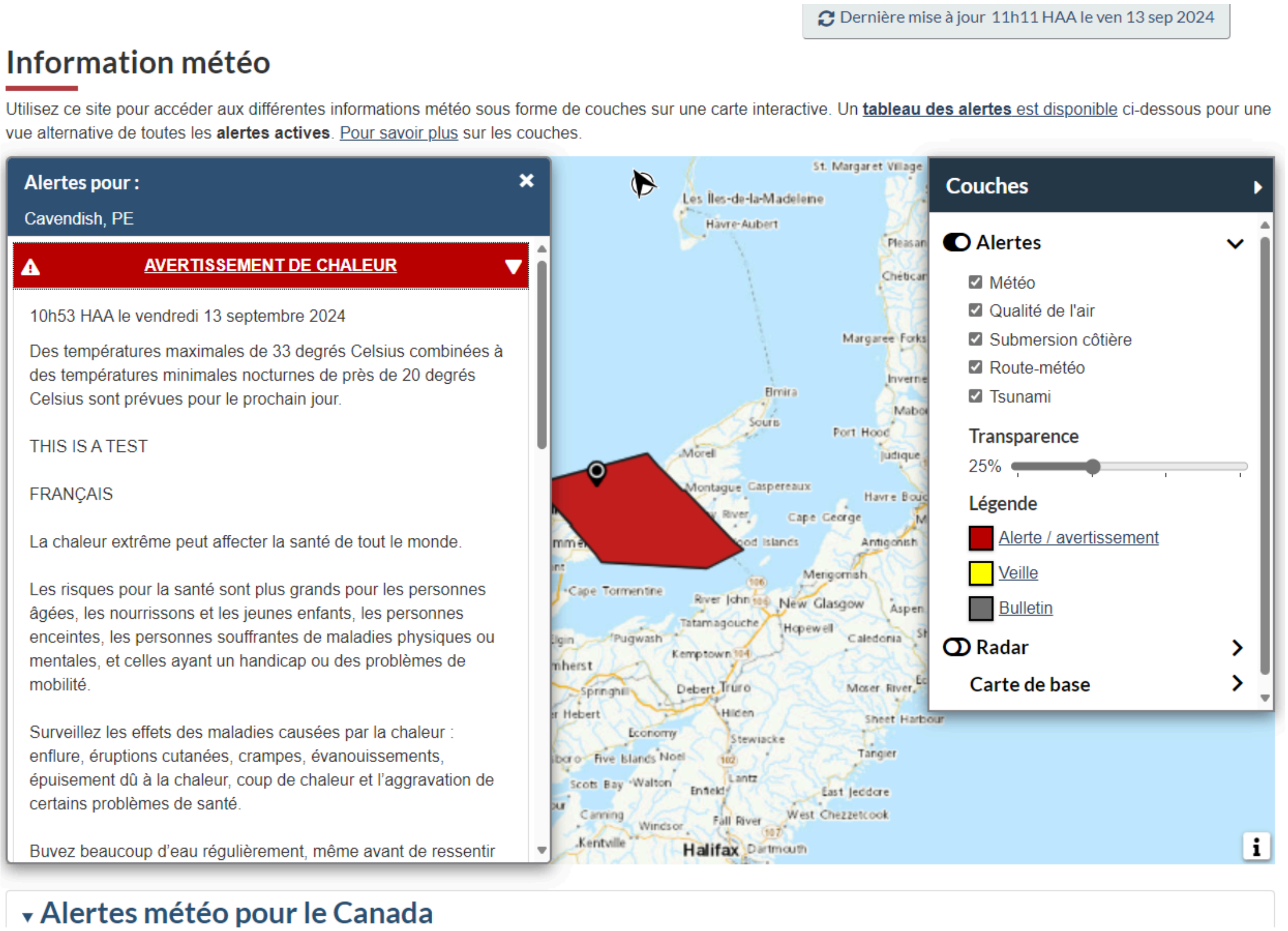

À la suite de la première ronde de consultations, nous avons apporté des modifications aux messages proposés. Pour chacun des messages révisés, veuillez examiner les modifications qui ont été apportées et répondre aux questions de consultation qui sont posées dans ce formulaire. Le cas échéant, veuillez fournir tout commentaire supplémentaire à l'appui de votre réponse.

Messages du système d'avertissement de chaleur d'ECCC : effets de la chaleur

	**Diffusés avec les alertes précoces** (de 24 à 48 heures avant un événement de chaleur)	**Diffusés avec l'alerte de chaleur** (pendant l'événement de chaleur)	**Diffusés lorsque l'événement prend fin** (à la fin de l'alerte)
**Version originale** (Ronde 1)	La chaleur extrême peut avoir une incidence sur la santé de tous. Préparez-vous à prendre des mesures afin de réduire vos risques. Les risques relatifs à la	La chaleur extrême peut avoir une incidence sur la santé de tous. Prenez des mesures afin de réduire vos risques. Les risques relatifs à la chaleur sont plus	La chaleur extrême peut avoir une incidence sur la santé de tous. Continuez à prendre des précautions afin de réduire vos risques. Les risques relatifs à la chaleur sont plus

	chaleur sont plus importants pour certains groupes.	importants pour certains groupes.	importants pour certains groupes.
**Version révisée** (Ronde 2)	Prenez des mesures pour réduire les risques pour vous – la chaleur extrême peut avoir des effets sur la santé de tous. Déterminez si vous ou votre famille êtes plus à risque de maladies associées à la chaleur.	Prenez des mesures pour vous protéger et protéger les autres – la chaleur extrême peut avoir des effets sur la santé de tous. Déterminez si vous ou votre famille êtes plus à risque de maladies associées à la chaleur.	Continuez à prendre des précautions afin de réduire vos risques. Les maladies associées à la chaleur peuvent se manifester après la fin de l'événement de chaleur; vous devez donc continuer de vous surveiller et de surveiller les autres.

Questions de la consultation - effets de la chaleur

	Oui	Non	Je ne sais pas
8. Êtes-vous d'accord avec les modifications proposées pour ces messages?	○	○	○
9. Ces messages devraient-ils être inclus dans le système d'avertissement météorologique d'ECCC?	○	○	○

Commentaires supplémentaires : si vous avez répondu « Non » ou « Je ne sais pas » à l'une des questions précitées, veuillez fournir une explication et tout autre commentaire que vous jugez nécessaire.

Le nombre de caractères est limité à 5 000, espaces comprises

Messages du système d'avertissement de chaleur d'ECCC : maladies liées à la chaleur et premiers soins

	**Diffusés avec les alertes précoces** (de 24 à 48 heures avant un événement de chaleur)	**Diffusés avec l'alerte de chaleur** (pendant l'événement de chaleur)	**Diffusés lorsque l'événement prend fin** (à la fin de l'alerte)
**Version originale** (Ronde 1)	La chaleur peut provoquer une déshydratation et des maladies associées à	Surveillez les premiers signes d'épuisement en raison de la chaleur : maux de tête, nausées,	Les signes et symptômes de la chaleur peuvent continuer à se

	la chaleur, notamment des gonflements, des éruptions cutanées, des crampes, des évanouissements, un épuisement attribuable à la chaleur et l'aggravation de problèmes de santé préexistants. Surveillez les premiers signes d'une maladie attribuable à la chaleur, car ils peuvent évoluer vers des situations d'urgence potentiellement mortelles.	vertiges, soif, urines foncées. Cessez la pratique de votre activité et reposez-vous. Déplacez-vous dans un endroit frais. Si vous êtes seul, prévenez un membre de votre famille, un voisin ou encore un ami. Retirez tous les vêtements inutiles. Appliquez des compresses froides ou encore des linges humides sur votre peau. Buvez de l'eau afin de remplacer les liquides. Si les symptômes ne disparaissent pas, composez le 911.	développer même après la fin d'un événement de chaleur extrême. Continuez à vous surveiller et à surveiller les autres.
**Version révisée** (Ronde 2)	Surveillez les premiers signes d'une maladie attribuable à la chaleur chez vous et chez les autres, car ils peuvent évoluer vers des situations d'urgence potentiellement mortelles. La chaleur peut provoquer une déshydratation et des maladies associées à la chaleur, notamment des gonflements, des éruptions cutanées, des crampes, des évanouissements, un épuisement attribuable à la chaleur et l'aggravation de problèmes de santé préexistants.	Surveillez les premiers signes d'une maladie attribuable à la chaleur chez vous et chez les autres, lesquels signes peuvent comprendre les suivants : maux de tête, nausées, vertiges, soif, urines foncées et intense fatigue. Cessez la pratique de votre activité et reposez-vous. Déplacez-vous dans un endroit frais. Si vous êtes seul, prévenez un membre de votre famille, un voisin ou encore un ami. Retirez tous les vêtements superflus. Appliquez des compresses froides ou encore des linges humides sur votre peau. Buvez de l'eau afin de remplacer les liquides. Si vos symptômes persistent, composez le 911.	Continuez de vous surveiller et de surveiller les autres pour déceler tout signe de malaise ou de maladie attribuable à la chaleur. Des maladies attribuables à la chaleur peuvent se manifester même après la fin de l'avertissement de chaleur.

Questions de la consultation - Maladies liées à la chaleur et premiers soins

	Oui	Non	Je ne sais pas
10. Êtes-vous d'accord avec les modifications proposées pour ces messages?	○	○	○
11. Ces messages devraient-ils être inclus dans le système d'avertissement météorologique d'ECCC?	○	○	○

Commentaires supplémentaires : si vous avez répondu « Non » ou « Je ne sais pas » à l'une des questions précitées, veuillez fournir une explication et tout autre commentaire que vous jugez nécessaire.

Le nombre de caractères est limité à 5 000, espaces comprises.

Messages du système d'avertissement de chaleur d'ECCC : urgences liées à la chaleur

	**Diffusés avec les alertes précoces** (de 24 à 48 heures avant un événement de chaleur)	**Diffusés avec l'alerte de chaleur** (pendant l'événement de chaleur)	**Diffusés lorsque l'événement prend fin** (à la fin de l'alerte)
**Version originale** (Ronde 1)	Un coup de chaleur est une urgence médicale – Composez immédiatement le 911 ou votre numéro d'urgence local si vous soupçonnez souffrir d'un coup de chaleur.	Si vous, ou quelqu'un de votre entourage, avez cessé de transpirer, avez la peau rouge, chaude et sèche, des vertiges, de la confusion, des nausées, une soif extrême, veillez à consulter immédiatement un médecin. Essayez de rafraîchir la personne immédiatement en la déplaçant dans un endroit frais, en enlevant les vêtements superflus, en appliquant de l'eau froide ou des poches de glace autour du corps et en l'éventant.	Prêtez attention à ce que vous ressentez et à ce que ressentent les personnes qui vous entourent. Les effets de la chaleur peuvent continuer à se faire sentir même après la fin d'un événement de chaleur extrême. Continuez à surveiller les effets des maladies attribuables à la chaleur.

**Version révisée** (Ronde 2)	Composez immédiatement le 911 si vous croyez présenter un coup de chaleur. Un coup de chaleur est une urgence médicale.	Composez le 911 si vous, ou quelqu'un de votre entourage, ressentez un malaise ou présentez des signes de coup de chaleur, par exemple une peau rouge et chaude, des étourdissements, de la diarrhée, de la confusion, un niveau de conscience altéré, des nausées, une soif extrême ou des changements au niveau de la transpiration. En attendant l'arrivée des secours médicaux, essayez de rafraîchir la personne en la déplaçant dans un endroit frais, en retirant ses vêtements superflus, en appliquant de l'eau froide ou des blocs réfrigérants autour de son corps et en l'éventant.	Prêtez attention à ce que vous ressentez et à ce que ressentent les personnes qui vous entourent. Les effets de la chaleur peuvent continuer à se faire sentir même après la fin d'un événement de chaleur extrême. Continuez à surveiller les effets des maladies attribuables à la chaleur.

Questions de la consultation - Urgences liées à la chaleur

	Oui	Non	Je ne sais pas
12. Êtes-vous d'accord avec les modifications proposées pour ces messages?	○	○	○
13. Ces messages devraient-ils être inclus dans le système d'avertissement météorologique d'ECCC?	○	○	○

Commentaires supplémentaires : si vous avez répondu « Non » ou « Je ne sais pas » à l'une des questions précitées, veuillez fournir une explication et tout autre commentaire que vous jugez nécessaire.

Le nombre de caractères est limité à 5 000, espaces comprises.

Messages du système d'avertissement de chaleur d'ECCC : surveillance

	**Diffusés avec les alertes précoces** (de 24 à 48 heures avant un événement de chaleur)	**Diffusés avec l'alerte de chaleur** (pendant l'événement de chaleur)	**Diffusés lorsque l'événement prend fin** (à la fin de l'alerte)
**Version originale** (Ronde 1)	Préparez un plan pour organiser des visites périodiques et soutenir votre famille, vos amis et votre communauté pendant les jours de forte chaleur.	Prendre couramment des nouvelles des personnes âgées ou vulnérables, en personne ou par téléphone, plusieurs fois par jour, en particulier le soir lorsque la température intérieure est élevée.	Continuez à surveiller les personnes âgées et les autres personnes vulnérables pendant quelques jours, car les températures intérieures peuvent demeurer élevées.
**Version révisée** (Ronde 2)	Parlez avec votre famille, vos amis et vos voisins pour savoir comment ils se préparent à la chaleur. Établissez un plan prévoyant des visites régulières et des moyens de vous soutenir mutuellement, en particulier les personnes les plus à risque.	Prenez des nouvelles des personnes âgées et des autres personnes à risque (enfants, femmes enceintes, personnes à mobilité réduite), en personne ou par téléphone, plusieurs fois par jour.	Continuez à surveiller les personnes âgées et les personnes à risque pendant quelques jours, car les températures peuvent demeurer élevées à l'intérieur.

Questions de la consultation - Surveillance

	Oui	Non	Je ne sais pas
14. Êtes-vous d'accord avec les modifications proposées pour ces messages?	○	○	○
15. Ces messages devraient-ils être inclus dans le système d'avertissement météorologique d'ECCC?	○	○	○

Commentaires supplémentaires : si vous avez répondu « Non » ou « Je ne sais pas » à l'une des questions précitées, veuillez fournir une explication et tout autre commentaire que vous jugez nécessaire.

Le nombre de caractères est limité à 5 000, espaces comprises.

Messages du système d'avertissement de chaleur d'ECCC : hydratation

	**Diffusés avec les alertes précoces** (de 24 à 48 heures avant un événement de chaleur)	**Diffusés avec l'alerte de chaleur** (pendant l'événement de chaleur)	**Diffusés lorsque l'événement prend fin** (à la fin de l'alerte)
**Version originale** (Ronde 1)	Lorsque la température commence à augmenter, veillez à boire fréquemment de l'eau, en vue d'éviter la déshydratation, qui peut entraîner une maladie associée à la chaleur.	L'exposition à la chaleur, en particulier lorsque vous êtes physiquement actif, entraîne une perte de liquides au moyen de la transpiration. Buvez de l'eau avant même d'avoir soif afin de remplacer ces liquides.	Si vous avez souffert de déshydratation ou d'une maladie associée à la chaleur pendant l'événement de chaleur, consultez votre professionnel de la santé pour vous préparer à de futurs événements.
**Version révisée** (Ronde 2)	Buvez souvent de l'eau pour éviter la déshydratation, laquelle peut provoquer une maladie associée à la chaleur.	L'exposition à la chaleur cause une perte de liquides par la transpiration. Buvez de l'eau souvent et avant même d'avoir soif, afin de remplacer ces liquides.	S. O.

Questions de la consultation - Hydration

	Oui	Non	Je ne sais pas
16. Êtes-vous d'accord avec les modifications proposées pour ces messages?	○	○	○
17. Ces messages devraient-ils être inclus dans le système d'avertissement météorologique d'ECCC?	○	○	○

Commentaires supplémentaires : si vous avez répondu « Non » ou « Je ne sais pas » à l'une des questions précitées, veuillez fournir une explication et tout autre commentaire que vous jugez nécessaire.

Le nombre de caractères est limité à 5 000, espaces comprises.

Messages du système d'avertissement de chaleur d'ECCC : comment réduire les risques dans votre espace de vie

	**Diffusés avec les alertes précoces** (de 24 à 48 heures avant un événement de chaleur)	**Diffusés avec l'alerte de chaleur** (pendant l'événement de chaleur)	**Diffusés lorsque l'événement prend fin** (à la fin de l'alerte)
**Version originale** (Ronde 1)	Avant que les températures n'augmentent, informez-vous au sujet des stratégies pour refroidir votre domicile, y compris l'entretien des systèmes de climatisation, les ventilateurs et l'ouverture des fenêtres.<br><br>Avant que les températures n'augmentent, vérifiez les stratégies de rafraîchissement de votre domicile, y compris la présence de ventilateurs.	Faites fonctionner la climatisation de l'air, si vous en disposez d'un tel système, ou encore déplacez-vous dans un endroit plus frais du domicile (p. ex. le sous-sol), fermez les rideaux, les stores ou les toiles et/ou ouvrez les fenêtres pour créer un courant d'air transversal.<br><br>Utilisez un ventilateur pour vous aider à rester au frais et orientez le flux d'air dans votre direction. Remarque : les ventilateurs sont inefficaces à des températures très élevées, en particulier pour les personnes âgées.	Les températures intérieures peuvent demeurer élevées même après la fin d'un événement de chaleur. Continuez à surveiller la température de votre domicile et appliquez des stratégies de rafraîchissement si nécessaire.<br><br>Lorsque la température baisse, utilisez un ventilateur pour faire circuler l'air frais dans votre domicile.
**Version révisée** (Ronde 2)	Trouvez des moyens de garder votre espace de vie au frais et veillez à ce que la climatisation et les ventilateurs fonctionnent et à ce que les fenêtres s'ouvrent.	Mettez la climatisation en marche ou déplacez-vous dans un endroit plus frais de votre espace de vie. Fermez les rideaux, les stores ou les toiles et ouvrez les fenêtres pour créer un courant d'air transversal.	Vérifiez la température dans votre espace de vie et restez au frais en ouvrant les fenêtres et en utilisant un ventilateur pour faire entrer de l'air frais à l'intérieur.

		Utilisez un ventilateur pour vous aider à rester au frais. Remarque : à des températures très élevées (35 °C), les ventilateurs sont inefficaces pour refroidir le corps – utilisez-les toujours avec d'autres moyens de rester au frais.	

Questions de la consultation - Comment réduire les risques dans votre espace de vie

	Oui	Non	Je ne sais pas
18. Êtes-vous d'accord avec les modifications proposées pour ces messages?	○	○	○
19. Ces messages devraient-ils être inclus dans le système d'avertissement météorologique d'ECCC?	○	○	○

Commentaires supplémentaires : si vous avez répondu « Non » ou « Je ne sais pas » à l'une des questions précitées, veuillez fournir une explication et tout autre commentaire que vous jugez nécessaire.

Le nombre de caractères est limité à 5 000, espaces comprises.

Messages du système d'avertissement de chaleur d'ECCC : comment réduire les risques à l'extérieur

	**Diffusés avec les alertes précoces** (de 24 à 48 heures avant un événement de chaleur)	**Diffusés avec l'alerte de chaleur** (pendant l'événement de chaleur)	**Diffusés lorsque l'événement prend fin** (à la fin de l'alerte)
**Version originale** (Ronde 1)	Planifiez vos activités en plein air pendant les heures les plus fraîches de la journée ou reportez-les jusqu'à ce que l'événement de chaleur soit passé.	Limitez l'exposition directe au soleil et à la chaleur. Portez des vêtements légers, clairs et amples, un chapeau à large bord ou encore utilisez un parapluie	Restez prudent lorsque vous sortez à l'extérieur de votre domicile, car les températures demeurent élevées.

		pour réduire les risques de coups de soleil et de surchauffe.	
**Version révisée** (Ronde 2)	Planifiez vos activités en plein air pendant les heures les plus fraîches de la journée ou reportez-les après la fin de l'événement de chaleur. À l'extérieur, recherchez les endroits ombragés.	Limitez l'exposition directe au soleil et à la chaleur. Portez des vêtements légers, pâles et amples et un chapeau à large bord et appliquez un écran solaire pour réduire vos risques de coups de soleil et de surchauffe.	Restez prudent à l'extérieur, car les températures demeurent élevées.

Questions de la consultation - Comment réduire les risques à l'extérieur

	Oui	Non	Je ne sais pas
20. Êtes-vous d'accord avec les modifications proposées pour ces messages?	○	○	○
21. Ces messages devraient-ils être inclus dans le système d'avertissement météorologique d'ECCC?	○	○	○

Commentaires supplémentaires : si vous avez répondu « Non » ou « Je ne sais pas » à l'une des questions précitées, veuillez fournir une explication et tout autre commentaire que vous jugez nécessaire.

Le nombre de caractères est limité à 5 000, espaces comprises.

Messages du système d'avertissement de chaleur d'ECCC : la nuit

	**Diffusés avec les alertes précoces** (de 24 à 48 heures avant un événement de chaleur)	**Diffusés avec l'alerte de chaleur** (pendant l'événement de chaleur)	**Diffusés lorsque l'événement prend fin** (à la fin de l'alerte)
**Version originale** (Ronde 1)	S. O.	S. O.	S. O.
**Version révisée** (Ronde 2)	Prévoyez un espace frais pour dormir.	Dormez dans la partie la plus fraîche de votre	S. O.

		espace de vie et ouvrez les fenêtres s'il n'y a aucun danger à le faire. Prenez une douche fraîche avant de vous coucher et portez des vêtements légers et amples.	

Questions de la consultation - La nuit

	Oui	Non	Je ne sais pas
22. Êtes-vous d'accord avec les modifications proposées pour ces messages?	○	○	○
23. Ces messages devraient-ils être inclus dans le système d'avertissement météorologique d'ECCC?	○	○	○

Commentaires supplémentaires : si vous avez répondu « Non » ou « Je ne sais pas » à l'une des questions précitées, veuillez fournir une explication et tout autre commentaire que vous jugez nécessaire.

Le nombre de caractères est limité à 5 000, espaces comprises.

Messages du système d'avertissement de chaleur d'ECCC : espaces publics pour se rafraîchir

	**Diffusés avec les alertes précoces** (de 24 à 48 heures avant un événement de chaleur)	**Diffusés avec l'alerte de chaleur** (pendant l'événement de chaleur)	**Diffusés lorsque l'événement prend fin** (à la fin de l'alerte)
**Version originale** (Ronde 1)	Si vous ne disposez pas de stratégies de rafraîchissement à domicile, déterminez les endroits climatisés près de votre communauté où vous	Si vous vous trouvez dans un domicile surchauffé, rendez-vous dans un espace public frais de votre communauté.	Les températures intérieures peuvent demeurer élevées même après la fin d'un événement de chaleur. Continuez à surveiller la température de votre

	pouvez aller vous rafraîchir (p. ex. centre communautaire, bibliothèque, centre commercial).		domicile et appliquez des stratégies de rafraîchissement si nécessaire.
**Version révisée** (Ronde 2)	Déterminez les endroits climatisés ou frais dans votre communauté où vous pouvez aller (p. ex. centre communautaire, bibliothèque, magasins, parcs ombragés). Prévoyez une aide au transport, s'il y a lieu.	Vérifiez votre thermostat ou thermomètre. S'il fait chaud dans votre espace de vie, allez dans un lieu public frais comme un centre communautaire, une piscine, un parc avec jeux d'eau ou un lac.	Continuez à surveiller la température à l'intérieur de votre espace de vie et à le garder au frais, s'il y a lieu. Les températures peuvent demeurer élevées même une fois l'événement de chaleur terminé.

Questions de la consultation - Espaces publics pour se rafraîchir

	Oui	Non	Je ne sais pas
24. Êtes-vous d'accord avec les modifications proposées pour ces messages?	○	○	○
25. Ces messages devraient-ils être inclus dans le système d'avertissement météorologique d'ECCC?	○	○	○

Commentaires supplémentaires : si vous avez répondu « Non » ou « Je ne sais pas » à l'une des questions précitées, veuillez fournir une explication et tout autre commentaire que vous jugez nécessaire.

Le nombre de caractères est limité à 5 000, espaces comprises.

Messages du système d'avertissement de chaleur d'ECCC : la sécurité en voiture

	**Diffusés avec les alertes précoces** (de 24 à 48 heures avant un événement de chaleur)	**Diffusés avec l'alerte de chaleur** (pendant l'événement de chaleur)	**Diffusés lorsque l'événement prend fin** (à la fin de l'alerte)
**Version originale** (Ronde 1)	Ne laissez jamais des personnes ou encore des animaux à l'intérieur d'un véhicule	Ne laissez jamais des personnes ou encore des animaux à l'intérieur d'un véhicule	Ne laissez jamais des personnes ou encore des animaux à l'intérieur d'un véhicule

	garé lorsqu'il fait chaud à l'extérieur. Regardez à deux reprises avant de fermer à clé et de partir.	garé lorsqu'il fait chaud à l'extérieur. Regardez à deux reprises avant de fermer à clé et de partir.	garé lorsqu'il fait chaud à l'extérieur. Regardez à deux reprises avant de fermer à clé et de partir.
**Version révisée** (Ronde 2)	Ne laissez jamais des personnes ou des animaux à l'intérieur d'un véhicule garé. Vérifiez le véhicule avant de le verrouiller afin de vous assurer qu'il ne reste personne à l'intérieur.	Ne laissez jamais des personnes ou des animaux à l'intérieur d'un véhicule garé. Vérifiez le véhicule avant de le verrouiller afin de vous assurer qu'il ne reste personne à l'intérieur.	Ne laissez jamais des personnes ou des animaux à l'intérieur d'un véhicule garé. Vérifiez le véhicule avant de le verrouiller afin de vous assurer qu'il ne reste personne à l'intérieur.

Questions de la consultation - La sécurité en voiture

	Oui	Non	Je ne sais pas
26. Êtes-vous d'accord avec les modifications proposées pour ces messages?	○	○	○
27. Ces messages devraient-ils être inclus dans le système d'avertissement météorologique d'ECCC?	○	○	○

Commentaires supplémentaires : si vous avez répondu « Non » ou « Je ne sais pas » à l'une des questions précitées, veuillez fournir une explication et tout autre commentaire que vous jugez nécessaire.

Le nombre de caractères est limité à 5 000, espaces comprises.

Messages du système d'avertissement de chaleur d'ECCC : information et ressources

	**Diffusés avec les alertes précoces** (de 24 à 48 heures avant un événement de chaleur)	**Diffusés avec l'alerte de chaleur** (pendant l'événement de chaleur)	**Diffusés lorsque l'événement prend fin** (à la fin de l'alerte)
**Version originale** (Ronde 1)	Renseignez-vous au sujet des ressources communautaires locales, des autorités de santé publique et	Surveillez les avertissements de chaleur et suivez les recommandations d'Environnement	Si votre domicile est encore chaud, communiquez avec votre municipalité pour connaître les centres

	des services d'urgence, et préparez une liste de numéros de personnes-ressources ou de liens Internet où vous pouvez obtenir de l'aide.	Canada ou encore de l'autorité de santé publique de votre région.	de rafraîchissement disponibles près de chez vous et pour obtenir des conseils et des services disponibles dans la communauté pour lutter contre la chaleur.
**Version révisée** (Ronde 2)	Renseignez-vous sur les ressources communautaires, les autorités sanitaires et les services d'urgence de votre région qui peuvent vous aider à rester au frais et à l'abri de la chaleur.	Surveillez les alertes de chaleur en cours en consultant le site Web sur les alertes météo publiques ou l'application MétéoCAN. Suivez les recommandations des autorités de santé publique de votre région.	Conservez sous la main des contacts utiles et des liens vers des sites Web traitant de la chaleur et de la santé, afin d'être prêt lors du prochain événement de chaleur.

Questions de la consultation - Information et ressources

	Oui	Non	Je ne sais pas
28. Êtes-vous d'accord avec les modifications proposées pour ces messages?	○	○	○
29. Ces messages devraient-ils être inclus dans le système d'avertissement météorologique d'ECCC?	○	○	○

Commentaires supplémentaires : si vous avez répondu « Non » ou « Je ne sais pas » à l'une des questions précitées, veuillez fournir une explication et tout autre commentaire que vous jugez nécessaire.

Le nombre de caractères est limité à 5 000, espaces comprises.

Messages du système d'avertissement de chaleur d'ECCC : consultation médicale

	Diffusés avec les alertes précoces	Diffusés avec l'alerte de chaleur	Diffusés lorsque l'événement prend fin

	(de 24 à 48 heures avant un événement de chaleur)	(pendant l'événement de chaleur)	(à la fin de l'alerte)
**Version originale** (Ronde 1)	Avant l'arrivée des beaux jours, demandez à un professionnel de la santé (p. ex. un pharmacien) comment les médicaments ou l'état de santé peuvent avoir une incidence sur les risques relatifs au soleil et à la chaleur.	Continuez à prendre vos médicaments, comme ils vous ont été prescrits. Vous ne devez pas modifier la façon dont vous prenez vos médicaments, et ce, sans avoir consulté au préalable votre médecin ou votre pharmacien.	Si vous avez souffert d'une maladie associée à la chaleur ou si vous vous êtes senti mal à cause de la chaleur, consultez votre professionnel de la santé afin de vous préparer à d'autres événements de chaleur.
**Version révisée** (Ronde 2)	Vérifiez si vos médicaments ou votre état de santé vous exposent à un risque accru en cas d'événement de chaleur. Consultez un professionnel de la santé pour obtenir plus de conseils à ce sujet.	Continuez à prendre vos médicaments tels qu'ils vous ont été prescrits, à moins d'avis contraire de la part de votre professionnel de la santé.	Demandez à un professionnel de la santé de vous indiquer comment réduire vos risques lors du prochain événement de chaleur.

Questions de la consultation - Consultation médicale

	Oui	Non	Je ne sais pas
30. Êtes-vous d'accord avec les modifications proposées pour ces messages?	○	○	○
31. Ces messages devraient-ils être inclus dans le système d'avertissement météorologique d'ECCC?	○	○	○

Commentaires supplémentaires : Si vous avez répondu « parfois » ou « non » à l'une des questions ci-dessus, veuillez expliquer.

Le nombre de caractères est limité à 5 000, espaces comprises.

Messages du système d'avertissement de chaleur d'ECCC : travailleurs à risque

	**Diffusés avec les alertes précoces** (de 24 à 48 heures avant un événement de chaleur)	**Diffusés avec l'alerte de chaleur** (pendant l'événement de chaleur)	**Diffusés lorsque l'événement prend fin** (à la fin de l'alerte)
**Version originale** (Ronde 1)	Les personnes exposées à la chaleur sur leur lieu de travail (à l'extérieur et à l'intérieur) devraient consulter leur employeur afin de se préparer à la saison chaude qui s'annonce.	Les travailleurs à l'extérieur et à l'intérieur devraient prendre des pauses programmées dans un espace ombragé ou plus frais avec une bonne ventilation (flux d'air), idéalement en position assise ou allongée. Retirez l'équipement de protection excédentaire et continuez à vous hydrater.	Les travailleurs à l'extérieur et à l'intérieur doivent continuer à faire preuve de prudence et à appliquer des mesures visant à réduire le risque de stress attribuable à la chaleur, car les températures restent élevées.
**Version révisée** (Ronde 2)	Parlez à votre employeur afin de vous préparer avant une chaleur extrême.	Prévoyez des pauses dans un lieu plus frais ou ombragé où il y a une bonne circulation d'air. Retirez l'équipement de protection excédentaire (s'il est sécuritaire de le faire) et continuez à boire de l'eau. Lorsque vous travaillez à l'extérieur, limitez votre exposition directe au soleil et à la chaleur. Portez des vêtements légers et de couleur pâle.	Restez prudent et prenez des mesures pour réduire votre risque de maladies liées à la chaleur, car les températures peuvent demeurer élevées sur votre lieu de travail.

Questions de la consultation - Travailleurs à risque

	Oui	Non	Je ne sais pas
32. Êtes-vous d'accord avec les modifications proposées pour ces messages?	○	○	○
33. Ces messages devraient-ils être inclus dans le système d'avertissement météorologique d'ECCC?	○	○	○

Commentaires supplémentaires : si vous avez répondu « Non » ou « Je ne sais pas » à l'une des questions précitées, veuillez fournir une explication et tout autre commentaire que vous jugez nécessaire.

Le nombre de caractères est limité à 5 000, espaces comprises.

Messages du système d'avertissement de chaleur d'ECCC : cuisson des repas

	**Diffusés avec les alertes précoces** (de 24 à 48 heures avant un événement de chaleur)	**Diffusés avec l'alerte de chaleur** (pendant l'événement de chaleur)	**Diffusés lorsque l'événement prend fin** (à la fin de l'alerte)
**Version originale** (Ronde 1)	Planifiez vos repas pour qu'ils ne nécessitent pas l'utilisation du four ou d'autres appareils générateurs de chaleur, en vue de limiter la chaleur intérieure supplémentaire.	Évitez les repas qui nécessitent l'utilisation du four et d'autres appareils générateurs de chaleur en vue de limiter la chaleur intérieure supplémentaire.	Continuez à préparer des repas qui ne nécessitent pas l'utilisation du four ou d'autres appareils générateurs de chaleur en vue de limiter la chaleur intérieure supplémentaire.
**Version révisée** (Ronde 2)	Planifiez vos repas de manière à ce qu'ils ne nécessitent pas l'utilisation du four ou de la cuisinière afin de limiter l'apport supplémentaire de chaleur dans votre espace de vie.	Planifiez des repas qui ne requièrent pas l'utilisation du four ou de la cuisinière, lesquels génèrent plus de chaleur. Cela vous aidera à garder votre espace de vie plus frais.	Continuez à préparer des repas qui ne requièrent pas l'utilisation du four ou de la cuisinière afin de garder votre espace de vie plus frais.

Questions de la consultation - Cuisson des repas

	Oui	Non	Je ne sais pas
34. Êtes-vous d'accord avec les modifications proposées pour ces messages?	○	○	○
35. Ces messages devraient-ils être inclus dans le système d'avertissement météorologique d'ECCC?	○	○	○

Commentaires supplémentaires : si vous avez répondu « Non » ou « Je ne sais pas » à l'une des questions précitées, veuillez fournir une explication et tout autre commentaire que vous jugez nécessaire.

Le nombre de caractères est limité à 5 000, espaces comprises.

Messages du système d'avertissement de chaleur d'ECCC : la chaleur extrême et les messages spéciaux ou avis sur la qualité de l'air

	**Diffusés avec les alertes précoces** (de 24 à 48 heures avant un événement de chaleur)	**Diffusés avec l'alerte de chaleur** (pendant l'événement de chaleur)	**Diffusés lorsque l'événement prend fin** (à la fin de l'alerte)
**Version originale** (Ronde 1)	S. O.	S. O.	S. O.
**Version révisée** (Ronde 2)	Prévoyez de vérifier la cote air santé dans votre région et de réduire votre exposition à la fumée des feux de forêt.	Lors d'un événement de chaleur extrême qui s'accompagne de fumée de feux de forêt, la priorité est de rester au frais.	Si la qualité de l'air s'est améliorée, ouvrez les fenêtres et les portes pour faire entrer l'air frais pendant la nuit, si cela ne pose aucun danger.

Questions de la consultation - La chaleur extrême et les messages spéciaux ou avis sur la qualité de l'air

	Oui	Non	Je ne sais pas
36. Êtes-vous d'accord avec les modifications proposées pour ces messages?	○	○	○

	Oui	Non	Je ne sais pas
37. Ces messages devraient-ils être inclus dans le système d'avertissement météorologique d'ECCC?	○	○	○

Commentaires supplémentaires : si vous avez répondu « Non » ou « Je ne sais pas » à l'une des questions précitées, veuillez fournir une explication et tout autre commentaire que vous jugez nécessaire.

Le nombre de caractères est limité à 5 000, espaces comprises.

Questions de la consultation

Ce processus de consultation vise à garantir que les messages diffusés sont complets, qu'ils sont axés sur l'action et fondés sur des données probantes et qu'ils tiennent compte des critères de lisibilité, d'équité et d'applicabilité régionale. En tenant compte de l'ensemble des messages, veuillez répondre aux questions qui suivent :

	Oui	Parfois	Non
38. Les messages proposés sont-ils importants pour le système d'alerte météorologique d'ECCC?	○	○	○
39. Les messages proposés sont-ils importants aux moments indiqués (avant, pendant et après l'événement)?	○	○	○
40. Les messages proposés sont-ils suffisamment orientés vers l'action (p. ex. favoriseraient-ils un changement de comportement)?	○	○	○
41. Les messages proposés incitent-ils à prendre les mesures requises aux moments indiqués?	○	○	○

	Oui	Parfois	Non
42. Les messages proposés sont-ils fondés sur des données probantes?	○	○	○
43. Le cas échéant, les messages proposés comprennent-ils les avertissements conditionnels nécessaires (p. ex. les limites pour des populations à risque particulières)?	○	○	○
44. Les messages proposés sont-ils rédigés à un niveau de lecture qui convient au grand public (c'est-à-dire, la 6e année du primaire)?	○	○	○
45. Les messages proposés sont-ils exempts de jargon ou de termes complexes?	○	○	○

Oui Parfois Non

	Oui	Parfois	Non
46. Les messages proposés sont-ils soucieux de l'équité?	○	○	○
47. Les messages proposés prévoient-ils des mesures de protection contre la chaleur réalisables pour des personnes de différents milieux socioéconomiques?	○	○	○
48. Les messages proposés sont-ils applicables à votre région géographique?	○	○	○
49. Les messages proposés reflètent-ils de manière appropriée les diverses conditions climatiques au Canada (p. ex. environnements intérieurs/extérieurs, événements prolongés, événements plus graves, événements aggravés)?	○	○	○

Hiérarchie

Le météorologiste en chef peut choisir de ne diffuser que quelques messages durant un événement de chaleur donné. Veuillez classer les messages de 1 à 15, 1 représentant le message le plus important à diffuser lors d'un événement de chaleur et 15, le moins important.

☐ Prenez des mesures pour vous protéger et protéger les autres – la chaleur extrême peut avoir des effets sur la santé de tous. Déterminez si vous ou votre famille êtes plus à risque de maladies associées à la chaleur.

☐ Surveillez les premiers signes d'une maladie attribuable à la chaleur chez vous et chez les autres, lesquels signes peuvent comprendre les suivants : maux de tête, nausées, vertiges, soif, urines foncées et intense fatigue. Cessez la pratique de votre activité et reposez-vous. Déplacez-vous dans un endroit frais. Si vous êtes seul, prévenez un membre de votre famille, un voisin ou encore un ami. Retirez tous les vêtements superflus. Appliquez des compresses froides ou encore des linges humides sur votre peau. Buvez de l'eau afin de remplacer les liquides. Si vos symptômes persistent, composez le 911.

☐ Composez le 911 si vous, ou quelqu'un de votre entourage, ressentez un malaise ou présentez des signes de coup de chaleur, par exemple une peau rouge et chaude, des étourdissements, de la diarrhée, de la confusion, un niveau de conscience altéré, des nausées, une soif extrême ou des changements au niveau de la transpiration. En attendant l'arrivée des secours médicaux, essayez de rafraîchir la personne en la déplaçant dans un endroit frais, en retirant ses vêtements superflus, en appliquant de l'eau froide ou des blocs réfrigérants autour de son corps et en l'éventant.

☐ Prenez des nouvelles des personnes âgées et des autres personnes à risque (enfants, femmes enceintes, personnes à mobilité réduite), en personne ou par téléphone, plusieurs fois par jour.

☐ L'exposition à la chaleur cause une perte de liquides par la transpiration. Buvez de l'eau souvent et avant même d'avoir soif, afin de remplacer ces liquides.

☐ Mettez la climatisation en marche ou déplacez-vous dans un endroit plus frais de votre espace de vie. Fermez les rideaux, les stores ou les toiles et ouvrez les fenêtres pour créer un courant d'air transversal. Utilisez un ventilateur pour vous aider à rester au frais. Remarque : à des températures

très élevées (35 °C), les ventilateurs sont inefficaces pour refroidir le corps – utilisez-les toujours avec d’autres moyens de rester au frais.

[ ] Limitez l’exposition directe au soleil et à la chaleur. Portez des vêtements légers, pâles et amples et un chapeau à large bord et appliquez un écran solaire pour réduire vos risques de coups de soleil et de surchauffe.

[ ] Dormez dans la partie la plus fraîche de votre espace de vie et ouvrez les fenêtres s’il n’y a aucun danger à le faire. Prenez une douche fraîche avant de vous coucher et portez des vêtements légers et amples.

[ ] Vérifiez votre thermostat ou thermomètre. S’il fait chaud dans votre espace de vie, allez dans un lieu public frais comme un centre communautaire, une piscine, un parc avec jeux d’eau ou un lac.

[ ] Ne laissez jamais des personnes ou des animaux à l’intérieur d’un véhicule garé. Vérifiez le véhicule avant de le verrouiller afin de vous assurer qu’il ne reste personne à l’intérieur.

[ ] Surveillez les alertes de chaleur en cours en consultant le site Web sur les alertes météo publiques ou l’application MétéoCAN. Suivez les recommandations des autorités de santé publique de votre région.

[ ] Continuez à prendre vos médicaments tels qu’ils vous ont été prescrits, à moins d’avis contraire de la part de votre professionnel de la santé.

[ ] Prévoyez des pauses dans un lieu plus frais ou ombragé où il y a une bonne circulation d’air. Retirez l’équipement de protection excédentaire (s’il est sécuritaire de le faire) et continuez à boire de l’eau. Lorsque vous travaillez à l’extérieur, limitez votre exposition directe au soleil et à la chaleur. Portez des vêtements légers et de couleur pâle.

[ ] Planifiez des repas qui ne requièrent pas l’utilisation du four ou de la cuisinière, lesquels génèrent plus de chaleur. Cela vous aidera à garder votre espace de vie plus frais.

[ ] Lors d’un événement de chaleur extrême qui s’accompagne de fumée de feux de forêt, la priorité est de rester au frais.

Renseignements supplémentaires (section facultative)

Veuillez nous faire part de tout commentaire supplémentaire qui, selon vous, devrait être pris en compte pour la mise à jour des messages sur la chaleur et la santé de Santé Canada.

Le nombre de caractères est limité à 5 000, espaces comprises.

[ ]

Submit Message

Soumettre

Si vous souhaitez modifier vos réponses, veuillez le faire avant de cliquer sur le bouton « Soumettre » ci-dessous.

Pour que vos commentaires soient pris en compte, vous devez cliquer sur le bouton « Soumettre ».

Powered by Qualtrics
