# Supplementary material for: Updating Health Canada’s Heat-Health Messages for the Environment and Climate Change Canada Heat Warning System: A Collaboration with Canadian Experts
Source: Int J Environ Res Public Health. 2025 Aug 13;22(8):1266. doi: 10.3390/ijerph22081266 (PMC12386431; doi:10.3390/ijerph22081266)
Supplement: Supplementary file 1 [file ijerph-22-01266-s001.zip › IJERPH_Supplementary Material File S7_Descriptive Results.pdf]

Updating Health Canada's Heat-Health Messages for the Environment and Climate Change Canada Heat Warning System: A Consultation Process with Canadian Experts

Supplemental File G

Table of Contents

Demographics 3

ECCC Heat Warning System Messages 4

Message 1: Heat Impact 4

Readability 4

Additional Comments 4

Message 2: Heat Illness 8

Readability 8

Additional Comments 8

Message 3: Emergency 10

Readability 10

Additional Comments 10

Message 4: Check-Ins 12

Readability 12

Additional Comments 12

Message 5: Hydration 14

Readability 14

Additional Comments 14

Message 6: Risk in the Home 16

Readability 16

Additional Comments 16

Message 7: Risk Outdoors 19

Readability 19

Additional Comments 19

Message 8: Cooking 21

Readability 21
Additional Comments 21
Message 9: Cooling Spaces 23
Readability 23
Additional Comments 23
Message 10: Car Safety 26
Readability 26
Additional Comments 26
Message 11: Workers 27
Readability 27
Additional Comments 27
Message 12: Medical Consultation 29
Readability 29
Additional Comments 29
Message 13: Information and Resources 31
Readability 31
Additional Comments 31
Message 14: Air Quality 33
Readability 33
Additional Comments 33
Message 15: Nighttime 35
Readability 35
Additional Comments 35
General Comments 37

Demographics

Table S1. Participant descriptors.

Question	Count *n* (%)
**Did you participate in the first round of consultation?**	
Round 1 Only	11 (26%)
Round 2 Only	15 (36%)
Both	16 (38%)
**Which of the following best identifies your organization?**	
Academia/Research	9 (21%)
Regional Authority	0 (0%)
Provincial/Territorial Authority	19 (19%)
Federal Health Authority	3 (3%)
Federal Meteorological Services	11 (11%)
**How long have you been employed in this field?**	
<5	6 (15%)
6-10	12 (29%)
11-15	7 (17%)
16-20	7 (17%)
21-25	6 (15%)
26-30	0 (0%)
>31	3 (7%)
**What is your highest level of educational attainment?**	
Elementary School	0 (0%)
Secondary School Diploma (or equivalent, e.g., GED)	0 (0%)
College Certificate or Diploma	1 (2%)
Bachelor's Degree	13 (31%)
Master's Degree	12 (29%)
Doctorate (PhD and MD)	15 (26%)
Prefer Not to Disclose	1 (2%)
**Which province/territory do you work in?**	
British Columbia	5 (12%)
Alberta	1 (2%)
Saskatchewan	1 (2%)
Manitoba	2 (5%)
Ontario	12 (29%)
Quebec	8 (19%)
New Brunswick	2 (5%)
Newfoundland and Labrador	5 (12%)
Nova Scotia	3 (7%)
Prince Edward Island	1 (2%)
Yukon	2 (5%)
Northwest Territories	2 (5%)
Nunavut	2 (5%)
Canada (Federal)	3 (7%)
**Please provide 3 to 5 keywords which best describe your area of expertise.**	
Meteorology, Weather Impacts, and Forecasting	14 (33%)

Climate Change and Climate Adaptation	8 (19%)
Communications and Risk Communication	7 (17%)
Environmental Public Health	6 (14%)
Heat	4 (10%)
Health and Health Promotion	4 (10%)
Emergency Preparedness, Management and Disaster Mitigation	4 (10%)
Physiology, Heat Physiology and Thermoregulation	3 (7%)
Public Awareness, Engagement and Outreach	3 (7%)
Equity and Social Inequities	2 (5%)
Epidemiology	2 (5%)
Cardiovascular System	1 (2%)
Impacts	1 (2%)
Air	1 (2%)
Behavioural Science	1 (2%)
Social Psychology	1 (2%)
Climate Justice	1 (2%)
Community Resilience	1 (2%)
Risk Analysis	1 (2%)
Housing	1 (2%)
Alerting	1 (2%)

ECCC Heat Warning System Messages

Message 1: Heat Impact

Readability

	Flesch Kincaid Reading Ease			Flesch Kincaid Grade Level			Gunning Fog Score			Smog Index			Automated Readability Index			Words			Complex Words		
**Original**	-	42	-	-	11	-	-	10	-	-	7	-	-	12	-	-	25	-	-	3	-
**Revised 1**	50	28	68	8	15	7	14	19	8	7	13	6	11	17	7	10	71	27	3	16	2
**Revised 2**	73	73	65	5	5	6	8	9	12	6	6	8	6	6	8	22	20	22	3	3	5
**Revised 3**	77	69	55	5	6	9	8	7	11	6	6	9	4	5	8	25	27	25	3	3	5
**Final**	70	50	57	6	11	9	8	12	11	6	8	9	6	11	8	20	39	26	2	4	5

Additional Comments

Respondent Comments
“There are many messages in this consultation. I would remove this one if we have to remove the most general ones. Proposed revisions: during the alert, include examples of measures. To avoid duplication, after the alert: "You must therefore continue to monitor yourself and others." monitor instead of the 1st monitor.”
“First two mention family, 3rd one says "others." Consistent?”
“Here is my proposal for the message broadcast when the event ends: Continue to take precautions to reduce your risks. Heat-related illnesses can occur after the heat event has ended. You must continue to monitor your health and that of others.”

“The wording is exclusive – ‘you or your family.’ Suggest ‘you or those around you.’ Post events reword suggestion: Heat illness may develop 2-5 days after the event has ended. Continue to monitor indoor air temperatures and for signs of heat illness in yourself and others. Column 2 suggestion: Extreme heat can affect everyone's health. Protect yourself and others, especially if you or your family are at greater risk of heat illness. For all the Event Ends messaging- has consideration been given to adding the ‘2-5 days’ timeframe to help focus the audience on the duration of potential effects? For columns 1-2, leading with the messaging ‘Extreme Heat can affect everyone's health,’ provides a focus that everyone can be impacted. Suggest leading with this as in Round 1. Still unclear how/what resources will be available for the audience to ‘determine if you are at risk.’”
“In the Revised (Round 2) - Released with Heat Alert: "determine if you or your family..." shouldn't that have already been done (and is already in the early warning table)? The statement asks people to take action to protect themselves - perhaps we could provide the top action they could take? I like the revised levels moving through the actions: Prepare - Take Action – Continue.”
“I think it would be relevant to guide people to resources to determine if they are at risk.”
“Will precautions be defined? There may be confusion unless specific directions are given.”
“I like how the action is at the beginning of the message in the revised version. It tells me right away what to do.”
“I have no issues with the heat-health messages being included in the current warning system during the event as we do now, but I have some concerns with statements included with early warnings and alert ends: 1. Currently, we have no early warnings. Warnings are only being issued 1 day before the event. 2. By default, when alerts are to be ended, there are no call-to-action items selected but a pre-defined sentence, “Conditions no longer existed for your areas” or something like that. 3. When the warning alert is ended, the city page will be flagged with end banner. I'm not sure how many people will still click to check the message, so releasing those post-event heat-health messages with alert ends is not an efficient way. 4. If we want to include them in the warning by stages, the warning system or the forecasters need to decide the stages with a warning update every time. The workload is a big concern if the forecaster needs to check different groups of messages with different stages manually.”
“I agree with the change, except rather than 'family,' perhaps 'loved ones and neighbours' may be more suitable and inclusive?”
“The revised statements are better, but the watch (pre) and warning (during) wording does not state what actions to take. Also, for the alert stage, the "determine if you or your family are at greater risk of heat illness" seems misplaced as the user is already being told to take action.”
“The messages don't indicate what actions to take. Presumably, the reader would need to do their research?”
"‘Determine if you or your family are at greater risk...’ should have a link attached to it, to allow individuals to see risk factors and local interventions.”
“The term vulnerable person does not seem equivalent to a person at risk. For example, older people are vulnerable to heat due to their senescent physiology. In contrast, a construction worker is at risk of heat exposure but not necessarily vulnerable. We also intend to maintain this distinction in our written productions. We do not see a definition or explanation of what constitutes an extreme heat event. Revised version, first and second columns: Determine if you or any family members are at higher risk. We would consider it complex to ask the population to define whether their loved ones are more at risk than others. Details about risk factors in certain subgroups of the population could be provided. The third column could specify: “You should, therefore, continue to monitor your symptoms and those of others. Bulk comments: (1) Although the reports are true theoretically, extreme heat can impact everyone's health. In practice, this confuses the message and may miss its target. (2) some subgroups are much more at risk or vulnerable than others and should be named. (3) There is a lot of talk about reducing risks, but we do not define them. In healthy people, only heat stroke is relevant. So, reducing risks simply means protecting yourself from the heat. For vulnerable people, it’s the same thing.”
“Suggest more direct language and an accessible link to risk factor info. Heat illness versus health risk from heat (exacerbation of pre-existing conditions). It says "take action" - but the action is a risk assessment, which might not seem like "action" by a layperson. There is not much of a lag effect with heat; maybe the message after the event should be more generic (Check in on your loved ones and yourself after the heat event). Early: Extreme

heat can affect everyone's health, some more than others. Determine if you or your loved ones are at greater health risk. During: Take action to protect yourself and others, especially if you are at greater risk."
"I agree that the rewording provides refocused intent on the most important parts of the behavioural change asked of the public."
"See commentary about sub-population groups that are especially susceptible to heat illness above and hopefully include them in the warning call to action statements. Great job otherwise."
"I feel that specific messaging related to the equitable groups listed on the previous page would be very beneficial. I think the more messaging, the better it can be used appropriately. Generic messaging works for most; however, … there is more flexibility in attaching specific messaging to specific events, considering the different equitable groups. For example - if forecasters know that the event is in an area with higher high-rise buildings and low-income populations, messaging can target the need to seek cooler environments, the language can be to seek AC spaces as this would be quite common in areas with these types of structures."
"If we accept that poverty is one of the biggest risk factors for heat-related injury and death, the messages could be more focused on the most at-risk population."
"Vulnerable populations pre-during and post are the same and do not help because vulnerable isn't defined, nor does it recommend action. Workers were identified as a group."
"I would suggest identifying groups and explain how."
"Yes, generally, but they could be more specific in some cases, such as susceptible populations (who are most at risk)."
"I think other specific-at-risk populations could be included as per my previous comments. My expertise does not allow for me to respond to question #11 appropriately."
"Re: question 12, this might be my ignorance, but is there a list of vulnerable groups on any of the websites used in the alert or from HC? or is the list too long to consider inclusion? Do all vulnerable groups know they're vulnerable? Do all people with those who are at-risk in their lives know that those folks are at-risk? Is it implied enough in the other messages? I'm not sure."
"It may be helpful to list the specific groups that have higher heat-related risks."
"I think we also need to mention physical impairments, those who can't drive themselves to a cooling centre, pregnancy, drug use."
"Vulnerable Populations: Heat-related risks are greater for specific groups. - vague throughout the pre-during and post-heat event messaging."
"It would be helpful if "otherwise vulnerable people" could be specified."
"Pre-heat event/Vulnerable Populations: I'm not sure this statement motivates any action. Perhaps restate or add along these lines: "Determine if you, your family and friends are among specific groups at greater risk…" Is the intent to identify or link to some of these groups as listed later on in the survey instrument? Make sense."
"Define who "specific groups" are."
"Vulnerable populations - no ID and no action for all time points."
"The vulnerable population's messages are not action-oriented in isolation. Consider identifying whether an individual is vulnerable to heat. The action is not clear in the car safety section."
"I think the messaging can be even more action-oriented. For example, during a heat event, messaging could target specific groups of the population that should take this advice directly, such as who is most impacted and what actions they should each take. This is considered in some cases, but in others, it could be more direct, for example, "The at-risk population should..., the general public should not... I also feel the messaging could be more targeted to specific groups, such as New Canadians and tourists not used to humidity, heat, or both, and Indigenous populations and how they would be culturally represented in the messaging. Can immigrant workers, the unhoused population or those with poor living conditions (limited heat mitigation or insulation) be addressed specifically?"
"We should be more specific and discuss elderly people with chronic illnesses or psychotic disorders."
"This document speaks of "heat," "high heat" or even "heat event." It seems better to use a generic term such as heat wave, which is often used in publicized forecasts."
"Specify what a "heat event" is."

“I think some recommendations could be more concrete: who are the "vulnerable" groups?”
“Add risk aggravated by alcohol consumption (even occasional).”
“The issues specific to certain populations vulnerable to heat are not always well understood.”
“Vulnerable populations: identify these populations so that those affected can identify themselves or their loved ones?”
“Repercussions of heat: Related messages can limit concerns about the heat issue and potential credibility. Many individuals feel little concerned or at risk from the heat (because of their age or state of health, for example). Furthermore, the health issues mentioned in connection with heat often affect mortality in the elderly and young children. Thus, a message stating that "severe heat can have an impact on EVERYONE'S health" may cause some people to reject the message or not seek further information, feeling safe or perceiving the message as exaggerated or alarmist.”
“Some messages (mainly heat impacts and vulnerable populations) offer little information as they currently stand.”
“Effects of heat: Before the event: Clarify the term “prepare yourself” by giving key actions such as making sure you have water available in the coming days, keeping your indoor environment cool, making sure you have stable communication with your loved ones who are vulnerable (e.g.). After the event: Why is the message different than before the event? Add that the effects of heat can persist over time to explain the relevance of continuing to take precautions.”
“Which risk groups: child; over 65 years old; sick; workers exposed to heat.”
“Certain wordings need to be reviewed: not sunny days, nor hot days but heat waves.”
“A definition or clarification of vulnerable populations should be made.”
“Proposal for vulnerable people: people aged over 65, people who commonly use drugs and alcohol, people who are overweight or obese, people who are pregnant or breastfeeding, people with developmental or behavioural disorders, cognition or mental health, including dementia, depression, schizophrenia and Alzheimer's disease, people with reduced mobility, People with chronic illnesses, including heart disease, hypertension, kidney disease, metabolic diseases, neurological diseases, respiratory diseases and cancer, people who are homeless or do not have safe shelter, people living in overcrowded and understaffed, high-density housing and without indoor air conditioning such as CHSLDs, people who do not have access to transportation, new arrivals to Canada and people facing language barriers, people who have suffered a power outage due to other weather events, people who are socially isolated or live alone, people working outside.”
“Undecided response: infants, babies and children (exclude children), people who use certain prescription medications and dietary supplements (exclude people taking dietary supplements), people who have a history of heat illness or who have ever suffered from heatstroke, Indigenous peoples, people living on the upper floors of multi-story buildings (specify the height of the building and the presence or absence of air conditioning), racialized peoples, people living in rural communities or isolated with reduced access to social services, people living in large urban centers with reduced access to green spaces, people with low socio-economic status, people lacking acclimatization, people living in buildings without air conditioning, people doing intense physical exercise.”
“Do not include tourists, passing populations, or people wearing PPE. I would separate people and situations at risk: doing intense physical exercise, working outside, participating in a large gathering outdoors or in a confined space without air conditioning, etc.”
“Specify what a “heat event” is.”

Message 2: Heat Illness

Readability

	Flesch Kincaid Reading Ease			Flesch Kincaid Grade Level			Gunning Fog Score			Smog Index			Automated Readability Index			Words			Complex Words		
**Original**	62	8	-	10	17	-	14	16	-	10	12	-	15	21	-	22	21	-	3	4	-
**Revised 1**	44	31	45	12	13	13	18	17	15	13	12	10	15	14	15	41	36	24	10	9	3
**Revised 2**	42	71	55	12	5	9	18	8	11	13	6	8	15	5	7	39	58	25	10	7	4
**Revised 3**	56	77	63	9	5	8	13	7	7	10	5	6	11	4	8	42	70	28	8	6	2
**Final**	-	-	-	-	-	-	-	-	-	-	-	-	-	-	-	-	-	-	-	-	-

Additional Comments

**Respondent Comments**
“The messages are still too long. I am not sure that they will be able to be read by people with little literacy. Suggestion: To allow the eye to understand that the two messages (before and during) are different, I suggest "Stay tuned" for the first message and "Watch" for the second. To avoid duplication, after the alert: "continue to monitor yourself and others." monitor instead of the 1st monitor. During the alert: 1st sentence too long and cut in 2: "Watch for the first signs of heat illness in yourself and others. These signs may include the following..."
“For the message broadcast with the heat alert, in the end, I would replace the sentence: "Drink water to replace liquids" with "Drink water to rehydrate." For the message that comes out when the event ends, I would replace the sentence, "Continue to monitor yourself and others for signs of heat-related illness or illness" with "Continue to monitor your health, as well as the health of others, for any signs of heat-related illness or illness."
“Consider presenting 911 as 9-1-1 throughout. For column #1, "Know the signs and symptoms of heat illness and what to do," vs the watch for early signs- as this is pre-event. Consider a link to Heat Illness resources. For column 3: Even though a heat warning may have ended, heat illness may not peak until a few days after the onset of extreme heat. Continue to monitor yourself and others for signs of heat-related illness.”
“Continuing with the prepare - take action - continue thread: The early warnings could start with "Prepare: Familiarize yourself with the early signs of heat illness.”
“The following sentence seems a bit complex to me: "Watch for the first signs of heat illness in yourself and others, which may include the following...". Maybe simplify it: "Watch for the first signs of heat illness in yourself and others, such as: ..."
“During 'pre-event' (first column), it makes sense to 'watch for...' but during the event (second column), the second sentence assumes that one (or others) is experiencing these symptoms. Suggest starting the first sentence with: "If you experience or see others experiencing symptoms like..., stop your activity and rest."
"‘Stop your activity, and rest.’ Not clear. Is this for the active exercising outdoors or does this mean everyone that is outside should stop what they are doing?”
“Again, I like how the messages were rearranged in the revised version to put the action right up front.”
“In the "Released with Early Warnings" guidance, heat stroke should be added to the consequences of heat. "evolve into" could probably be simplified to "lead to" - In the "Released with Heat Alert" guidance, something like "if you experience so and so" should be added before "Stop your activity, and rest.”
“I respond "I don't know" to a lot of the (following) questions regarding including it in ECCC messaging; unless otherwise stated, what these "I don't know" responses mean is that they seem like valuable messaging that could be beneficial to be part of the heat warnings, but I have concerns (which we've talked about as a group previously) about the length of those messages and how including all these phrases and their length and how

forecasters would pick and choose. Some of the details seem perhaps like they'd be better as communication points issued during alerts."
"I like the inclusion of what actions to take. However, the alert stage wording is rather long, so I would want to edit it to include only the necessary wording. The post-event message sounds strange - could remove "feeling unwell or"."
"911 doesn't exist in all of Canada, so I would ask that it say 'call the emergency health care provider or go directly to your nearest health centre.'"
"First aid pre-event (early warning) should include advice to stock up on drinking water/ice, fans, and food that does not require stoves. Post-event - how long after the event should you keep monitoring? "Most acute health events associated with extreme heat will manifest within 3 days," but there are chronic illnesses..."
"Certain wordings need to be reviewed: not illness but discomfort (or discomfort and illness)."
"We are confused by the fact that swelling, rashes, cramps, and fainting are associated with heat-related illnesses. Unless we are mistaken, these are symptoms, but not diseases. We suggest rewording: Heat can cause dehydration and heat-related illnesses. The first signs of these diseases include swelling, skin rashes, etc.<br>This distinction seems important because multiple factors can cause swelling, rashes, cramps, and fainting. It could be more simply stated: Make sure that if you have a chronic illness, it is well controlled, and be on the lookout for any symptoms of heat exhaustion (call 811 if necessary). The beginning of confusion constitutes a medical emergency. We suggest replacing the elements in the second column with: If you have new symptoms, do not hesitate to call 811 (and not 911). We also suggest replacing the items in the third column with: Continue to monitor your symptoms and those of others to detect…"
"Watch for early signs of illness from heat. These may include worsening of pre-existing conditions, headaches, nausea,..."
"Yes, these statements are what folks are looking to find before, during and on the tail end of the event, and likely, this is one of the only places they will get them if they are not consuming a lot of intel from the emergency management organization and health sector per se."
"Consider for the end of an event this wording: Even though a heat warning may have ended, heat illness may not peak until a few days after the onset of extreme heat. Continue to monitor yourself and others for signs of heat-related illness."
"The general public may not know what heat stroke or heat exhaustion is or looks like. It may be helpful to include symptoms beside the term so they know what to look for in these heat emergencies."
"Post-Heat event messaging may be ignored once the warning is lifted or confusing. Education will be needed on the new messaging."
"When we suggest that the effects of heat can be felt even after the delays, we should be more precise."
"Not illness but discomfort."
"Heat-associated illnesses - after giving back the signs to watch out for?"
"Heat-related illnesses and first aid: Before the event: Here we discuss heat-related illnesses in the pre-heat phase. This should be reported more in the "during" section of the event. Furthermore, don't the examples given refer more to heat-related symptoms than illnesses? It is much more important to ensure that illnesses are well controlled, whether for mental health, cardiac or respiratory health. We propose adding the symptom "intense fatigue" as a symptom during the event. "If the symptoms do not disappear after a period of rest." To stop practicing your activity: We mix two types of heat stroke. One is due to sport and work, which rarely kills and which affects a healthy clientele, and the other is much more dangerous, which affects the elderly and those who suffer from chronic illnesses. If you are alone, yes – but still – at any age and in all conditions? Once again, it is better to target the at-risk audience. After: Give a time horizon."

Message 3: Emergency

Readability

	Flesch Kincaid Reading Ease			Flesch Kincaid Grade Level			Gunning Fog Score			Smog Index			Automated Readability Index			Words			Complex Words		
Original	-	51	-	-	12	-	-	14	-	-	10	-	-	12	-	-	75	-	-	8	-
Revised 1	64	51	55	7	11	9	11	13	11	8	9	8	6	11	7	96	100	25	16	13	4
Revised 2	32	48	74	14	13	6	18	16	8	13	11	6	11	14	6	19	51	38	5	7	3
Revised 3	55	58	79	7	11	5	13	14	7	7	10	5	2	12	3	15	68	38	4	8	3
Final	79	55	75	4	12	6	8	16	8	6	11	6	0	12	5	17	73	37	2	11	3

Additional Comments

Respondent Comments
“I don't know about the weather service recommending you call 911, although I do like having the reference to it rather than not. I found it OK when it was after the "signs of" but now that "call 911" is first, it seems drastic and might seem alarmist.”
“For the early warning message, I would replace the phrase, "Call 911 immediately if you think you have heat stroke." with "Call 911 immediately if you think you have (signs/symptoms) of heat stroke.”
“Suggest wording change in column 3: ‘Continue to watch for the signs of heat illness.’"
“911 isn't available in all jurisdictions … - I prefer the original Call 911 or your local emergency line. I also don't think this should be in the Early Notification column. This should be a preparedness message. Something like ‘Prepare: familiarize yourself with the symptoms of heat illness and be on the lookout during this upcoming heat event.’"
“Although the following sentence is correct, it is not natural to read or understand: "If you, or someone around you, feels unwell" (because we say "you feel," but it can sound strange with "someone around you"). Maybe adjust: "Call 911 as soon as you feel unwell or signs of heat stroke or someone you know."
“Signs of heat stroke should likely be included when heat warnings are forecast. - During the event, it should also be specified to be ready to start CPR if the person goes unconscious or shows no signs of circulation.”
“Also, is the heat emergency needed with early warnings if the early warning is 24-48 hours in advance of a heat event?”
“I think it may be a bit too early to say, "Call 911 immediately if heat stroke is suspected. Heat stroke is a medical emergency" leading up to a heat warning. Maybe something like this would be better. Pay close attention to how you and those around you feel. Call 911 immediately if a heat stroke is suspected. Heat stroke is a medical emergency.”
“This is similar to the ‘Heat-related illness and first aid’ statement. Forecasters would have to not issue these two simultaneously for redundancy purposes. Should the Heat Emergency statement be prioritized over the Heat-related illness statement if choosing between them?”
“Revised wording is better, but for the warning stage, I would not include "feeling unwell" in the first sentence - rather, go straight to "signs of heat stroke." Again, I would want to edit to remove some of the superfluous wording, or suggestions that may be better in FAQs or infographics. In warning messaging, less is more sometimes.”
“Early warning - include ‘Ensure you have a phone nearby to call 911 should you or your loved ones experience a heat stoke because it is a medical emergency.’"

"We suggest replacing the first message with: Call 811 immediately if you or someone close to you has heat stroke. This proposal is based on the fact that one of the early symptoms of heatstroke is confusion. So, in extreme heat, any confusion can be a sign of heatstroke. It is proposed to limit the list of symptoms in the second column and keep only red and hot skin, dizziness, nausea, confusion, and altered levels of consciousness. Indeed, the emergency occurs with confusion and alteration of the state of consciousness (here, serious emergency)."
"With early warning, provide a link for more info on heat stroke."
"Two things: heat emergency is also used in BC to designate an extraordinary heat event (an Extreme Heat Emergency), a 2nd tier to the heat warning by ECCC. Language is important, so although heat stroke is an emergency, it might be good to consider those words carefully in any literature on the subject. Also, in the early alert (before the heat), wouldn't it help those preparing to have the list of symptoms of what to look for?"
"This also assumes calling 911 is acceptable by everyone."
"Some words could be substituted to be easier for the general public to understand: e.g. while seated or reclined > while sitting or laying down."
"Questions are mostly fine. One I would change is the one about heat stroke. "If you, or someone around you, has stopped sweating, has red, hot, dry skin, dizziness, confusion, nausea, extreme thirst - seek immediate medical attention." Exertional heat stroke does not typically present with hot try skin. It should be clear that "Heat stroke can present with X, Y Z. However, know that each case is different and all symptoms don't appear all the time". For the fans part, it should likely describe what a very high temperature is."
"Yes, generally, but they could be more specific in some cases, such as signs of heat stroke (altered level of consciousness being a key one)."
"During the event/Last two rows: Some inconsistency in recommending people "call 911" for the heat-related illness and not the heat emergency (seek immediate medical attention)."
"For athletes, you can get heat stroke and sweat a lot. We are still targeting poorly."
"We suggest calling 911 in the event of heat stroke in the "before the event" section, but this does not appear in the "during" the event section. This should appear in the "during" phase."
"Harmonize the messages in each box. Heat stroke is a medical emergency – Call 911 or your local emergency number immediately if you suspect you are suffering from heat stroke. If you or someone you know has stopped sweating, has red, hot, dry skin, dizziness, confusion, nausea, or extreme thirst, seek medical attention immediately. Try to cool the person immediately by moving them to a cool, shaded area, removing excess clothing, applying cold water or ice packs around the body, and fanning them."
"Heat emergency: During the event: add a message to call 911."
"Emergency - during and after: the "before" version mentions calling 911, but the other two versions do not. Also, consider repeating the symptoms to watch out for next?"
"Diarrhea is also a symptom of heat stroke."
"Certain wordings need to be reviewed: not exhaustion attributable to heat but Heat stroke."
"Throughout the text, we mix the classic heat stroke of heat stroke due to physical activity. The target audiences and vulnerabilities are different. For populations vulnerable to classic heatstroke, I would say it is those who have chronic illnesses that are not controlled and the very elderly."

Message 4: Check-Ins

Readability

	Flesch Kincaid Reading Ease			Flesch Kincaid Grade Level			Gunning Fog Score			Smog Index			Automated Readability Index			Words			Complex Words		
**Original**	-	81	-	-	4	-	-	6	-	-	4	-	-	5	-	-	15	-	-	1	-
**Revised 1**	58	51	-	9	10	-	11	13	-	8	10	-	10	10	-	32	48	-	4	8	-
**Revised 2**	54	24	36	11	17	13	14	21	14	10	15	12	11	17	12	19	28	19	3	7	4
**Revised 3**	75	62	67	6	8	7	9	8	6	7	6	6	5	8	4	33	27	21	4	2	2
**Final**	75	74	63	6	6	8	9	6	8	7	4	6	5	4	7	33	23	31	4	1	2

Additional Comments

Respondent Comments
“Suggestions for wording in French: Before the alert, replace ‘Talk with your family, friends and neighbours’ with ‘Keep in touch with those around you.’ In the end, eliminate ‘especially those most at risk.’ During the alert. Add ‘several times a day’ before ‘in person or by phone.’"
“Persons may not see themselves as at-risk. It should also include those living alone or socially isolated in column 2. Wording for column 2 suggestion: Check in on friends or family living alone or socially isolated. These individuals may not be aware of the impact of heat. Use the Health Checklist to check in on those you care for at least twice per day. (hyperlink in https://ncceh.ca/resources/evidence-reviews/health-checks-during-extreme-heat-events)”
“Maybe ‘especially FOR those most at risk.’ For the message during alerts, perhaps add a statement like ‘The health of these people can deteriorate quickly’ to explain why it is necessary to check in several times on the same day. For the message after, maybe avoid a verb like ‘watch.’ Maybe ‘keep checking in’.”
“Talk to family, friends and neighbours to see how they are preparing for the heat" could use more inclusive language to consider those who do not have social networks or are unhoused. Perhaps including seeking community resources.”
"For a few days after the event is declared over. The idea that temperatures can remain high indoors can also be brought up with previous warnings.”
“For 2) Released with Heat Alert (during heat event) and 3) Released when Event Ends (when alert ends), it would be helpful to have specific actions rather than 'check-on,' like 'call' or 'visit.'”
“When warning messages get too long, there is a tendency not to read the entire message. The recommendations listed here can be incorporated into a link where additional information/resources are provided.”
“I do see why the examples of otherwise at-risk people were included, but I fear it reads a bit exhaustive and doesn't include all at-risk groups. Also, I am aware of the inability to be exhaustive, but including those with mobility challenges but not those with cognitive/neurodevelopmental challenges is mostly where I am thinking.”
“The revised ‘watch’ stage message is unnecessarily long. Wording can be revised to be shorter for the alert stage as well.”
“We must demonstrate that children (except infants) constitute a group vulnerable to heat. Pending conclusive data on this subject, we propose removing the parentheses from the second column. We also suggest adding: Check in regularly or daily with elderly people and people with chronic illnesses (especially if they are not controlled).”
“Suggest - create a plan for regular check-ins (rather than visits, harder to do); during alert suggest adding at the end to check in ‘especially if they live alone.’"

"'At risk' people doesn't (but should) explicitly list those who are both older adults AND live alone, those with pre-existing health conditions such as diabetes, Heart disease or respiratory disease, mental illness such as schizophrenia, depression and anxiety, those with substance use disorders, marginally housed..."
"Check-ins also uses examples of higher risk individuals. should mention children, pregnant individuals, and those who use substances."
"Older adults are largely 'acted upon' when, in reality, they are most likely to be the ones you engage with. I would suggest not just making them folks to be checked in on. Some targeted messaging is critical given their frailty."
"Check-in messaging does not consider those without a support system cooling strategies, which could include using ice and a fan in the case of not having an air conditioning unit or central system with air conditioning."
"I think that some terminology can be adjusted to simplified terminology. For example: ex. frequently – often, ex. vulnerable - at-risk (this could be nitpicky)."
"I'm most concerned with the messaging for Vulnerable Populations. The messages refer to "specific groups," but it is unclear who is meant here and why "health-related risks" would be higher for them. Can we be more specific here for those who might not understand and not realize they are at higher risk? There are also no actions associated with this - e.g. check-ins or trying to ensure you are not alone."
"Pre-heat event/Check-ins: Instead of 'prepare a plan' it might be more effective to recommend that people 'Touch base with' or 'Talk to (Discuss with…) family, friends and neighbours to see how they are preparing for or concerned about the expected heat event.' [Making this initial contact makes it easier to follow up as recommended during and after the event]."
"Pre-heat event/heat Impacts: I would treat all preparation forms as 'actions,' thus suggest adjusting wording to "Take action to prepare for…OR Make preparations to…"
"Check in with others multiple times a day, especially if you or someone you know works outside, has a pre-existing health condition, and is a child or older adult. These individuals are at a significantly increased risk of severe injury or death."
"Combine more 'for you or a loved one at risk' for three main reasons: a) children, elderly people and the sick are less likely to do it and plan it themselves, b) a healthy, caring adult benefits from doing so remind you to think about it for a loved one; c) in general, people assess themselves as less at risk than they are and the message carries more weight if we think it is valid for someone else."
"Checks: Raise awareness of people at risk in your surroundings. E.g., "Assess who is vulnerable to heat in your family or community and make a plan to support them during the heat." During and after: among the first messages which give concrete advice. More posts should be like this. (e.g., repercussions of heat, vulnerable populations, workers at risk (before): messages in these categories provide little concrete and useful information)."
"Check-Ins: After the event: Complete with "and health risks can occur several days after the heat event." The target audience is far too broad. Visits must be made to people at risk. There are almost no deaths among young adults, and not many among healthy older adults, but many among the very old. During the event: If we had to prioritize one moment, it would be after several days of oppressive heat, regardless of the time of day. After the event, a few deaths were seen once the heat subsided. If the heatwave has passed, I recommend opening the windows instead."

Message 5: Hydration

Readability

	Flesch Kincaid Reading Ease			Flesch Kincaid Grade Level			Gunning Fog Score			Smog Index			Automated Readability Index			Words			Complex Words		
**Original**	-	49	-	-	10	-	-	11	-	-	8	-	-	9	-	-	25	-	-	4	-
**Revised 1**	54	55	-	10	11	-	13	13	-	10	10	-	10	12	-	51	42	-	8	6	-
**Revised 2**	34	67	45	14	8	13	18	9	15	13	7	10	14	9	15	24	29	24	5	3	3
**Revised 3**	54	89	-	9	3	-	11	3	-	8	4	-	8	4	-	14	24	-	2	1	-
**Final**	75	85	40	4	4	10	5	6	8	4	4	8	0	6	9	14	23	8	1	1	2

Additional Comments

**Respondent Comments**
"Suggestions for wording in English: Before the alert: keep sentences short. Cut the sentence into 2. Drink water often to avoid dehydration. It can cause heat-related illness."
"For the message broadcast with the heat alert, I would replace the sentence: "Exposure to heat causes a loss of fluids through sweating. Drink water often, even before you feel thirsty, to replace these liquids." "Exposure to heat causes a loss of fluids through sweating. Drink water often, even before you feel thirsty, to replace these liquids."
"Consider expanding options beyond water: Drinking plenty of water and other non-alcoholic, non-caffeinated beverages to stay hydrated. Dehydration can lead to heat illness. Column 2: Drink fluids often and before you feel thirsty."
"Again, the early notification should be a preparedness message. IE. Ensure you will have drinking water available. People aren't going to be concerned about heat illness at the time an EN is sent - preparedness is the key. The statement should be flipped during the heat event: "Drink water often...Exposure to heat...". This puts the most important action item first and the explanation second. This should be the case for all statements."
"Not having a message for the end of the alert does not seem to me to be consistent with the other sections that mention continuing to monitor for signs and symptoms. Wouldn't it be wise to send a message that recommends continuing to monitor hydration levels? Especially if the temperatures remain warm?"
"Having "N/A" for the post-event period is inconsistent with earlier statements that people continue to experience effects after the alert has expired because of the lag in health effects materializing and the lag in indoor temperature declines depending on the dwelling characteristics. Suggest something like: "Continue to drink water and stay hydrated...""
"Hyponatremia goes up during heat waves, so it might be a good idea here to encourage people to maintain normal meals (which should be enough to offset a salt loss in most people)."
"Similar statement to drink water is included in the "heat-related illness statement." Care would have to be taken by the issuing forecaster not to include both statements because of redundancy."
"Early Message: Drink water often to avoid dehydration, which can "promote" heat-related illness."
"Dehydration can exacerbate symptoms associated with certain illnesses but can also cause its conditions (orthostatic hypotension). Review the wording "which can cause/cause a heat-associated illness." In this regard, the best formulation to recommend in the second column seems to us to be the following: "Don't hesitate to hydrate yourself adequately, especially if you are thirsty." Furthermore, the invitation to consult a health professional does not seem clear to us; Specify the circumstances in which this recommendation could be useful without overloading the network."

"Exposure to heat will cause your body to lose fluids. Drink water often and..."
"Could probably simplify more. There's more formality to the language and more words than needed…For example, drink water frequently to avoid dehydration as the temperature rises. Could be: As it gets hot, drink water often to avoid getting dehydrated..."
"Some words could be substituted to be easier for the general public to understand: e.g. continue to hydrate > drink water to stay hydrated."
"Under hydration during the event, " including "especially when you are physically active," might cause those with mobility restrictions not to realize they are at risk, too. Exposure to heat will cause your body to lose fluids through sweat, whether you are physically active or not, but especially when you are physically active."
"A bit paradoxical to talk about extreme thirst without suggesting drinking."
"I think some recommendations could be more concrete: How much water should we drink?"
"How often and how much water? Number of ml (ounce) every 20 minutes (+ also the risk of over-hydration)."
"Not make sure you drink, but make sure you drink."
"Useful if specific: drink every 20 minutes."
"The proposed actions are sometimes vague. For example, 'drink water frequently.' How often should you drink water? More concrete recommendations would facilitate actions."
"Hydration: Before: Here, we discuss measures to take in the event of heat during the pre-heat phase. Those with medical conditions are at risk if they drink too much water. The message is entirely premature. We also mention here an illness associated with heat without defining it. During: Physically active people are not at risk. For athletes, this recommendation (drinking before feeling thirsty) is controversial, and no good evidence supports this recommendation. After: unnecessary recommendation: Too non-specific and not necessary for the vast majority of people."

Message 6: Risk in the Home

Readability

	Flesch Kincaid Reading Ease			Flesch Kincaid Grade Level			Gunning Fog Score			Smog Index			Automated Readability Index			Words			Complex Words		
**Original**	-	103	-	-	1	-	-	3	-	-	2	-	-	1	-	-	13	-	-	0	-
**Revised 1**	20	71	33	16	8	12	22	11	13	15	7	10	17	9	10	21	59	24	7	4	6
**Revised 2**	35	71	45	12	6	10	18	10	12	12	7	10	14	5	8	32	60	38	9	7	8
**Revised 3**	86	76	74	4	6	6	6	7	8	4	5	6	4	4	5	19	58	27	1	4	2
**Final**	86	70	73	4	7	6	6	9	8	4	6	6	4	7	3	19	42	33	1	3	3

Additional Comments

Respondent Comments
“Since there are a lot of messages, if we have to remove what is less essential, this page could be cut off. Suggestions for wording in French: "espace de vie" is not a common term in French, so use more familiar terms such as "your home," "your home," etc. Before the alert, it can be replaced with "Find ways to keep your [living space] cool: air conditioning and ventilation are two good examples." During the alert: There is a lot of information here. You have to prioritize what you want to communicate. After the alert: This is not like a message after the alert. It lacks fluidity. Suggestion: "Stay vigilant. Be sure to continue to refresh your [living space], if necessary.”
“I think it's good information but maybe more educational than in a warning. Especially the ‘Note’ about fans - that's a lot of detail to put in a warning message.”
“For the message that comes out with the early warnings, I would replace the phrase, "Find ways to keep your living space cool and make sure the air conditioning and fans are running and the windows are opening." with the following: "Find ways to keep your living space cool. Make sure that the air conditioning, as well as the fans, are working and that the windows can be opened." For the message broadcast with the heat alert, I would add a "+" or say it as a word in the following sentence: "Note: at very high temperatures (>35°C)... or "Note: at very high temperatures (35°C and above)..."
“For column 2: Elevated indoor temperatures, especially at night, can be extremely dangerous to health. Check your thermostat or indoor thermometer. If it’s hotter than 31°C during the day and 26°C at night, consider moving to a cooler location or take action to reduce indoor temperatures. For column 3, add on "living space..., when safe to do so."
“Prefer the original (again, preparedness) HA: Turn on air conditioning if available. It covers those that do not have air conditioning. EE: I prefer the original; I just flipped - action first, explanation second.”
“For the forward post, perhaps add a reference to a resource that advises on how to prepare for or cope with heat waves.”
“It's wrong to mention that fans are ineffective above 35 degrees Celsius. Evidence of their usefulness is up to 38C, even for older adults. Emitting this message minimizes the effectiveness of the fan and spreads false information. If that threshold is included, I am of the view that this message should not be broadcast. I would be pleased to discuss this with you.”
"Find ways to keep your living space cool and ensure air-conditioning, fans, and windows are working." It needs inclusive language for those who do not have housing. Seek community resources, visit air-conditioned facilities, etc.”
“The revised statements are a lot better. They are now clear and easy to understand.”

“For both the cross breeze and post heatwave advice, it might be good to specify "if outdoor temperatures are cooler than indoors."
“Not necessarily appropriate for those in all socio-economic conditions.”
“This information can be provided on a resource page.”
“Maybe some variation of these messages are valuable for the alerts, but again, I think this may be something where additional communication and education outside the alerts would be more valuable. I can share very personal anecdotes as someone who moved from a cooler part of Canada to a warmer part of Canada and how I didn't know what I could or could not do to keep my home cool in ways that may be obvious to people who have the experience. I almost feel that air conditioning could be part of the list of examples of how to cool one's home, but it is not prominently featured. I also understand, from living in a humid, warm place during the summer, that opening windows is not a good idea as the air outside is likely to be warm unless it is overnight when things have cooled off (and even then, if it is warmer than about 20 deg C outside, which is indicative of humidity typically, I have been advised to avoid opening the window overnight at this time, too).”
“Edits can be made to shorten length. ‘Close curtains, blinds, or shades, open windows to create a cross-breeze’ is slightly contradictory advice since creating across-breeze will be more difficult with window coverings.”
“Maybe discuss ways to monitor indoor temperature? Is feeling hot a good enough of an indicator? Buy a thermometer? Should we mention the link with wildfire smoke and how HEPA filters would be recommended? If we prioritize heat or smoke, we should prioritize staying cool.”
“We are doubtful about opening the windows. We recommend this avenue only if the outdoor environment is cooler than indoors (e.g. at night) and if this option does not cause discomfort (e.g. hearing) and security issues. We propose to change the order of the options presented, starting with the most accessible and ending with air conditioning, which is not within everyone's reach. We also question the relevance of the clarification in the third column: Check the temperature in your living space. For what purpose? Is a limit/benchmark temperature proposed?”
“Would ECCC scroll through this and other messages? If so, I think it could be helpful, but there might need to be some message about whether one can't stay cool or is having symptoms despite strategies and may need to find a different place to cool down.”
“Maybe the cross-breeze window recommendation needs to be said about when the outdoor (late evening) temperatures drop below indoor air temperature (which leads to having a thermometer handy in the living space)?”
“As previously stated, more focus on indoor temperatures is needed, especially in our region where AC is not prevalent.”
“Indoor cooling: I would add (1) install blinds in the pre-heat event message, (2) use a fan if there is no AC (although I now see there is a separate section for a fan), and (3) is there room here to remind folks to avoid fire smoke, but if must pick, prioritize heat mitigation.”
“Under the Indoor Cooling and Fan Use headings- what are the "cooling strategies" and where can they get more information on these options to develop a plan?”
“Under Fan Use and indoor cooling (AC): Consider wording for Pre-Heat Event: Ensure fans are in good working order.”
“These messages assume that people have a home (e.g., indoor cooling section).”
“Is there a resource or place where options for protective measures are available that could be linked?”
“I think that some terminology can be adjusted to simplified terminology. For example, ex. good ventilation (airflow) - just use ‘airflow.’”
“Some words could be substituted to be easier for the general public to understand: e.g. interior temperatures can remain high even after a heat event. Continue to monitor your home's temperature and apply cooling strategies as needed > Even after a heat event, your home can still be hot. Keep checking the temperature and use cooling methods if needed.”
“Some words could be substituted to be easier for the general public to understand: e.g. ensuring windows are operable > check that your windows can open.”

“I didn’t have time to review the literature to compare to the statements, but all of the recommended actions seem to be at least partially supported by available evidence. Certain statements could perhaps be clarified, such as at what indoor temperature/humidity level should regular fans be avoided? To me, the question comes back to the relative significance of the proposed actions in terms of intent, behavioural change, and improvements in risk outcomes. I’m not certain but doubt that this comparative information exists.”
“There is not enough focus on the risks of indoor temperatures, which cause most heat-related injuries and deaths. More prescriptive information on indoor temperatures is needed. Again, the post-event messages aren't of much value.”
“During the event/Indoor Cooling: I don’t believe telling people with air conditioning to turn it on is necessary. Instead, just say: “Move to a cooler area of the home (e.g., basement), close curtains, pull blinds or shades and open windows to create a cross-breeze if your home does not have air conditioning.”
“What is meant by "cooling strategies"? Where can specific actions be identified? Are there resources that can be linked in or messaging highlighting the potential actions to cool down be noted in the messaging options.”
“For the most part action-oriented, but the note for older adults about fans is vague - "Note: Fans are Ineffective at very high temperatures, especially for older adults". What are people supposed to do, when? If this is too difficult to nuance, is there a point at which fan use becomes harmful -- to help qualify the message.”
“Indoor cooling - missing the proactive blinds installation in pre-event.”
“Mention use of curtains and shadow areas in the Before column.”
“Sometimes lack of specificity: what temperature in a home is alarming?”
“Not indoor heat but the temperature in the home.”
“Some syntax inconsistencies: 'prepare yourself' at...YOUR risks (or otherwise all impersonal with verbs in the infinitive: prepare; take; avoid; withdraw; watch.”
“Add shade, close curtains during the day (sheets in windows).”
“Useful if specific: from what temperature? 30°C?”
“Some expressions used (e.g., a transverse air current) are not expressions commonly used by the general public.”
“Refreshing the interior: Before the event: We suggest simplifying it to: “Before temperatures rise, ensure strategies to cool your home are working, such as air conditioning, fans, or opening windows.” During the event, we recommend opening windows at night and closing them when it is sunny. “Turn on the air conditioning, if you have such a system,…” The term “transverse airflow” may not be adequately understood and should be explained.”
“Using a fan: Before the event: simplify the sentence to ‘Check the presence and operation of fans in your home.’ During the event: The message states that ‘fans are ineffective at very high temperatures.’ What is considered high temperature should be clarified.”
“Messages 'during' must all be communicated 'before' as well. To emit a behaviour, you must have known it, understood it, integrated it, planned it before.”
“Certain wordings need to be reviewed: not internal heat but heat from home (otherwise it can be understood as body heat).”

Message 7: Risk Outdoors

Readability

	Flesch Kincaid Reading Ease			Flesch Kincaid Grade Level			Gunning Fog Score			Smog Index			Automated Readability Index			Words			Complex Words		
**Original**	61	87	-	7	4	-	11	8	-	7	6	-	6	4	-	16	10	-	3	1	-
**Revised 1**	40	40	-	13	13	-	16	16	-	12	12	-	15	15	-	41	41	-	9	9	-
**Revised 2**	53	67	-6	11	8	17	12	10	16	8	7	12	12	10	16	21	29	10	2	3	4
**Revised 3**	68	74	33	6	6	10	7	7	6	5	5	6	5	7	8	26	31	9	2	2	2
**Final**	68	72	33	6	6	10	7	7	6	5	5	6	5	8	8	26	32	9	2	2	2

Additional Comments

**Respondent Comments**
"I think these "outdoor" risk messages are written well. They should be an option to include in alerts. Most of them are common sense, though. It is more important to reserve text within an alert to the higher risk statements to prevent the alert from being too wordy and too long."
"For the message with the heat alert, I would make some corrections to the following sentence: "Wear light, light, loose-fitting clothing and a wide-brimmed hat and apply sunscreen to reduce your chances of sunburn and overheating." to "Wear light, light, loose-fitting clothing, as well as a wide-brimmed hat. Also, apply sunscreen to reduce your chances of sunburn and overheating."
"Message during: I would like to remind you of the advice regarding physical activity and outdoor activities that are mentioned in the first message."
"If mentioning sunblock, note SPF 30 or higher and follow product instructions. Sunscreen will help protect from UV rays but not from the heat. Redness/pain from sunburn is temporary; the damage to the skin adds up throughout your life and can develop into serious long-term health effects such as skin cancer. For babies under 6mo old, consult a healthcare provider before applying sunscreen."
"Would be helpful to specify which hours of the day are the coolest (e.g., morning)."
"Again, there is not enough focus here on indoor heat, which is the real risk."
"This information can be included on a resource page."
"We propose to add the following message: Make sure you are close to a place to cool off (e.g. swimming pool) or shade during outdoor activities to take a break from the heat. We also propose slightly modifying the elements specified in the third column: Stay cautious outside if temperatures remain high."
"Umbrellas removed from items helpful during the event? Asian/African countries use them to great effect, why the omission?"
"Some words could be substituted to be easier for the general public to understand: e.g. continue being cautious when heading outdoors as temperatures remain elevated > Keep being careful when outside as outdoor temperatures stay high."
"During the event/Cooling Centres: Avoid prepositional phrasing in sentences. You could also make the sentence simpler (unless you have a specific indoor temperature threshold in mind) by saying something like, 'Find and move to a cool public space in your community or stay at a friend or relative's air-conditioned residence if you feel uncomfortably hot in your home.'"
"Pre-heat event/General: In the cooling categories, was consideration given to identifying community public swimming pools, splashparks, lakes/rivers, etc.? Also, if the recipient does have good air conditioning, it could be

an opportunity to suggest inviting other family, friends, and neighbours who do not have such cooling resources."
"Consider taking extra precautions to reduce your exposure to heat, such as reducing or rescheduling outdoor physical actions. This is particularly important for children, older adults, those with pre-existing health conditions and outdoor workers, as these individuals can experience earlier or more severe effects from heat."
"Not take steps but take actions."
"Add shade."
"Specifically add a grocery store, cinema."

Message 8: Cooking

Readability

	Flesch Kincaid Reading Ease			Flesch Kincaid Grade Level			Gunning Fog Score			Smog Index			Automated Readability Index			Words			Complex Words		
**Original**	-	115	-	-	-1	-	-	3	-	-	2	-	-	0	-	-	7	-	-	0	-
**Revised 1**	-	27	-	-	14	-	-	16	-	-	12	-	-	13	-	-	19	-	-	4	-
**Revised 2**	44	41	38	13	12	13	14	14	14	10	10	12	13	12	13	23	19	22	3	3	4
**Revised 3**	70	99	77	9	2	8	11	4	8	6	2	6	9	5	9	22	20	20	1	0	1
**Final**	-	-	-	-	-	-	-	-	-	-	-	-	-	-	-	-	-	-	-	-	-

Additional Comments

**Respondent Comments**
“If there are too many messages, we can remove them. Suggested wording: Before alert: change to shorter: "Plan meals that do not require the use of the oven or stove." I would act more during the alert: "Eat cold meals since the oven and stove generate heat."
“We're getting so specific here. I think to keep the alerts short, we shouldn't include specific statements like this.”
“Education (and common sense) not warning communication.”
“Column 1 wording revision suggestion: Plan meals that minimize the use of the oven or stove to limit additional heat in your indoor living space. Column 2/3- add "indoor" before living space.”
“Prepare - Take Action - Continue EN: Plan meals that don’t require the oven or stove, which makes more heat. This helps keep your living space cooler. HA: If possible, prepare meals that don’t require the oven or stove, which makes more heat. This helps keep your living space cooler.”
“Makes more sense to keep the message identical if possible and applicable (reinforcing effect). I prefer the 'during event' version.”
“Worry that too many items will be overwhelming. This one could be cut and slipped into the items about keeping homes cool. ‘Keep your home cool (e.g., use AC, limit cooking).’”
“The messages appear to be too long, and this piece may not be included.”
“The watch stage should have "plan meals..." wording, and then the warning stage should have "avoid meals..."
“It is proposed to add a recommendation regarding increased consumption of products with a higher water content (e.g. raw fruits and vegetables). Also, these messages could prove of little use in air-conditioned environments.”
“Not sure the last message is necessary; early warning can start similarly (Plan meals that don't require...); during heat event suggest "which add more heat" rather than ‘make more heat.’"
“During: "make meals..." instead of the plan?”
“Could probably simplify more. There's more formality to the language and more words than needed. For example, ‘Plan to ensure your meals don’t require the use of the oven or other heat-generating appliances to limit additional indoor heat’ could be ‘Plan meals that don't require the oven or other heat-generating appliances that add indoor heat.’”
“I think that some terminology can be adjusted to simplified terminology. For example heat-generating appliances - stoves and ovens that raise indoor temperatures.”

"Add Eat light, fresh foods rich in water (fruits, vegetables such as cucumber, salad)."
"Cook: Before: Really? Based on what evidence... We are still before the heating episode. After Opening windows is much more effective than changing eating habits."

Message 9: Cooling Spaces

Readability

	Flesch Kincaid Reading Ease			Flesch Kincaid Grade Level			Gunning Fog Score			Smog Index			Automated Readability Index			Words			Complex Words		
**Original**	-	70	-	-	9	-	-	11	-	-	6	-	-	10	-	-	23	-	-	1	-
**Revised 1**	31	-	-	12	-	-	18	-	-	12	-	-	9	-	-	26	-	-	8	-	-
**Revised 2**	54	55	33	8	10	12	14	14	13	9	10	10	6	8	10	29	17	24	7	3	6
**Revised 3**	65	77	61	6	5	7	8	8	8	6	6	7	3	4	4	28	29	26	5	3	4
**Final**	61	71	61	7	6	7	9	8	8	8	6	7	6	4	4	28	24	26	5	2	4

Additional Comments

**Respondent Comments**
“Wording suggestions: Before the alert: remove "or fresh" from the 1st sentence. We are targeting air-conditioned spaces especially. During the alert: Thermostat: This assumes that all homes have thermostats, which is not necessarily the case. Replace with "Check the temperature of your home"? Reduce the sentence to "... Go to a cool public place (e.g., community center, swimming pool, park, water games, lake)." After the alert, we can replace the 1st sentence with: ‘Continue to make sure to keep your [living space] cool.’”
“More public education from a community perspective would be setting up cooling centres and making people aware of public areas - maybe not appropriate for the federal government to try to squeeze this information into warnings.”
“Can the verbiage be shifted to cooling locations vs cooling spaces? The public may not see a mall or library as a "cooling space" as they aren't designed. For column #2- Check your thermostat or "indoor" thermometer. If it’s hotter than 31°C during the day and 26°C at night, consider moving to a cooler location or take action to reduce indoor temperatures. Want to give options- either move to a cooler location OR take action to reduce the temperatures in your location.”
“EW: Identify air-conditioned or cool spots where you can go if needed (e.g., community centers and other community buildings, stores, shaded areas). Plan for transport assistance if needed. HE: Check your thermostat or thermometer often. If you are in an overheated home, relocate to a cool public space in your community centre, swimming pool, splash park or lake - not available everywhere.”
“Message during: it says "if it's hot," but what is considered too hot? It might be important to mention this.”
“Consider saying ‘beach’ instead of ‘lakes.’”
“Might amend 'during event' message to emphasize shading even where there is access to the cooling effects of water "Move to a public cool, shaded public place..."
“Check your thermostat or thermometer. Move to a cool public space such as a community centre, swimming pool, splash park or lake if your living space is hot. Here is a suggestion: Check your thermostat or thermometer. If your living space is hot, move to a cool public space such as a community centre, swimming pool, splash park or lake.”
"Where you can go if your home becomes too hot.   The examples of cool spaces (e.g., community center, library, stores, shaded parks) should also be added to the during event guidance.”
“Watch (pre) stage wording is long - the first sentence should suffice. I find the original warning stage (during) wording better and more succinct than the revised, except flipping the order of the sentence to start with the action, i.e., "Relocate to a cool public space in your community if you find yourself in an overheated home."

"Include "Ocean" for some of our coastal communities. Mention that they still need to be covered/shaded from the sun while at those recreational water sites."
"Before the proposed messages, we question the consensual definition of a hot environment. The presentation of a reference temperature seems appropriate here to avoid ambiguity in interpreting the message. Continue to monitor the temperature inside your living space. For what purpose? Is a limit/benchmark temperature proposed?"
"Centre" in Canada, not "center" (see early warning column). Could this section explain what a "hot living space" is? 26 °C and 31°C are the increasing impact thresholds, I believe..."
"References to cooling centres or other community facilities may not apply to rural and remote communities."
"It would be beneficial to mention AC and pools where available and not where not available."
"Using the term "cooling space" or "cooling location" as opposed to cooling centers, as sometimes people don't see these as being the same as a cooling center- often denoting a special location which may not always be the case."
"Some words could be substituted to be easier for the general public to understand: e.g. plan ahead.. require the use of the oven or other heat-generating appliances to limit additional indoor heat > Plan your meals so you don't need to use the oven or other appliances that make heat. This helps keep your home cooler."
"I don't see the value in the post-event messages. Heat is generally a very acute hazard; continuing to check in on people, for example, is useless. Extending the warning for 24-48 would be better to let indoor environments cool down."
"Some messages are designed with a mindset focused on non-disabled and mobile persons with sound cognitive abilities. Some messages appear to be based on certain assumptions and, therefore, may not speak to someone living in a poorly maintained rental apartment. These folks may not have a basement they could go to and perhaps cannot relocate to a cool public space in their community without requiring assistance. There is a mention of older adults in some areas but no mention of people with physical and or mental challenges. Suggesting people to 'move' or 'relocate' as a risk reduction strategy does not holistically address the issue."
"There is a lack of post-heat event action items aimed at preparing for the NEXT event. Post-event is where we can nudge people to be better prepared for next time. For example, for air conditioning, we should refer people to HVAC specialists to help them ensure they have cooling and discuss strategies to reduce costs."
"Heat messaging should also offer tips (UBC Climate Hub has great resources on this)."
"There is a chance that no specific services or refreshment centers exist in the municipalities. It is not up to the federal government to say this because, once again, it may contradict local public health messages."
"The proposed protective measures all require resources, for example, electricity for the air conditioner/fan, travel to the cooling center, and social contact for follow-up by friends/relatives. More specific recommendations for marginalized/ low-resource populations may be missing."
"I think some recommendations could be more concrete: At what temperature should we avoid using a fan? Etc."
"Explain why fans can be ineffective or even harmful: increased risk of dehydration."
"Not shopping center (very urban), but store, grocery store."
"Cooling centers: During the event: From what temperature can a building be considered overheated and for how long? This should be clarified."
"The proposed actions are sometimes vague. For example, "fans are ineffective at very high temperatures." At what temperature are fans dangerous? More concrete recommendations would facilitate actions."
"Specify the cooling strategies."
"There is quite the controversy over AC vs Fan usage. Consider adding additional messaging to include key points from FANS: Fans are an effective cooling option when used appropriately. (1) Spin the ceiling fan blades counterclockwise on the highest setting during the summer. When your ceiling fan spins quickly in this direction, it pushes air down and creates a cool breeze. Although it does not cool the air in the room, the breeze will help

you feel cooler. (2) Fans should be used during the late evening and early morning hours to help move cooler air indoors. (3) Fans can be dehydrated, so keep drinking water to keep hydrated. (4) Fans should not be relied upon as a primary cooling method during extreme heat. When temperatures exceed 35°C, fans can make you feel hotter by blowing hot air over your skin and increasing your risk for heat illness. (5) Air conditioning removes heat from the air to cool off interior spaces and protects health in extreme heat. If feasible, install a window air conditioner in at least one room. More information on choosing a window air conditioning unit is available. (6) You can use fans to make a Do-It-Yourself (DIY) air conditioner by placing a shallow bowl of ice water in front of it. (7) If air conditioning is not an option for your residence, consider cooling down by visiting friends and family or locating a public space with air conditioning. (8) Passively cool your residence: Keeping heat outside and cool air inside helps to lower indoor temperatures by using strategies that require little to no energy consumption. Close your windows during the hottest parts of the day (~10 am-8 pm); If it is safe (consider air pollution, noise, crime), open windows and doors in the evening to cool your home passively; Hang wet towels or place bowls of water around your residence to increase evaporation; Passive cooling works best when you can open windows and doors opposite to each other or on different floors. You can also turn on an electric, bathroom, or kitchen fan to increase airflow."

Message 10: Car Safety

Readability

	Flesch Kincaid Reading Ease			Flesch Kincaid Grade Level			Gunning Fog Score			Smog Index			Automated Readability Index			Words			Complex Words		
**Original**	-	66	-	-	6	-	-	8	-	-	6	-	-	5	-	-	9	-	-	1	-
**Revised 1**	39	61	-	11	9	-	14	12	-	10	8	-	12	9	-	43	88	-	9	10	-
**Revised 2**	74	74	74	5	5	5	6	6	6	4	4	4	6	6	6	20	20	20	1	1	1
**Revised 3**	78	78	78	4	4	4	7	7	7	5	5	5	2	2	2	23	23	23	2	2	2
**Final**	66	66	66	6	6	6	8	8	8	6	6	6	4	4	4	25	25	25	3	3	3

Additional Comments

Respondent Comments
“Since there are several pages of messages, this one could be removed. Wording suggestions: 1st sentence can be replaced by: "Make sure you don't leave anyone in a parked vehicle."
“Not only parked vehicles but also running "closed vehicles." Suggestion: Check for children or pets before you exit your vehicle. Do not leave any person or pet inside a closed vehicle for any length of time.”
“HA: Never leave people or pets inside a parked vehicle. Check the vehicle before locking to make sure no one is left behind. (Add some sort of messaging that indicates it is life-threatening to do so?).”
"‘Never leave people or pets inside a parked vehicle’ should suffice. Second sentence is unnecessary.”
“We propose to specify: Never leave people (especially children) or animals... It would be interesting to specify that heat-related deaths among children are almost exclusively in cars parked with children left alone inside.”
“Very important message as unfortunately deaths still occur.”
“I'm not sure if this is the right place for this one, but never leave people or pets inside a parked vehicle when it is hot outdoors. Look twice before locking and leaving; the ‘when it's hot outdoors’ should be removed. It's never safe to leave pets and babies in cars, and it can still get quite hot in vehicles when it's mild outdoors.”
“Where is the ‘Look twice before locking and leaving.’ Where is the ‘look twice’ from?”
"What does ‘Look twice before locking and leaving’ mean?”
“What is the dangerous outside temperature for people in a closed car: 'hot' is too non-specific (25°C?).”
“Not hot days but heat waves.”

Message 11: Workers

Readability

	Flesch Kincaid Reading Ease			Flesch Kincaid Grade Level			Gunning Fog Score			Smog Index			Automated Readability Index			Words			Complex Words		
**Original**	-	65	-	-	7	-	-	8	-	-	6	-	-	10	-	-	11	-	-	1	-
**Revised 1**	55	62	-	11	9	-	9	11	-	6	7	-	14	11	-	23	36	-	1	3	-
**Revised 2**	55	57	40	11	9	13	9	10	14	6	7	10	14	11	16	23	31	23	1	3	3
**Revised 3**	85	78	62	4	5	10	4	6	12	2	4	10	7	6	12	9	40	22	0	2	3
**Final**	-	-	-	-	-	-	-	-	-	-	-	-	-	-	-	-	-	-	-	-	-

Additional Comments

Respondent Comments
"I don't think we need specific notices for people working outside. Protections for people in general apply to workers, and often it is company policy that will keep people at work or not during a heat event."
"Again, better as an awareness campaign with affected groups, or billboards, etc, not as warning content."
"For the message with the early warnings, I would replace the phrase, "Talk to your employer to prepare for extreme heat." with "Talk to your employer to prepare for extreme heat."
"Column 2: Consider adding " where possible" at the end."
"Take planned breaks in a shaded or cooler space with good airflow. Remove extra protective gear (if safe) and keep drinking water. When working outdoors, limit direct exposure to the sun and heat. If possible, wear lightweight, light-coloured clothing."
"The messages appear to be too long, and this piece may not be included."
"Talk to your workplace to prepare before the extreme heat > Talk to your workplace to prepare for extreme heat."
"While each individual needs to take responsibility for their wellbeing, I wonder if something like this can be directed towards the employers more so - many workers (e.g., undocumented migrant workers) cannot make such requests."
"I don't like "remove extra protective gear" wording. Also, there is some redundant wording that could be removed to shorten the message."
"…We suggest that you refer to the following link: https://www.cnesst.gouv.qc.ca/sites/default/files/publications/travailler-a-la-chaleur.pdf."
"The heat event message should be contingent on working in the heat (not applicable to offices with AC, so put a conditional statement up front). i.e., If working in the heat, take breaks in a shaded or cooler space. Limit direct exposure to the sun..."
*"Workers at Risk During Heat Event: Remove excess protective gear and continue to hydrate. * Limit: Remove excess protective gear when it is safe or resting, and keep hydrated. When working outdoors, limit direct exposure to the sun and heat. Wear lightweight, light-coloured clothing."*
"Pre-heat event/Workers at Risk: Possible alternative wording that begins with the action verb. "Learn about (OR Know, Become familiar with…) your rights, employer responsibilities, and workplace's policies concerning excessive heat."

“Particularly, some of the 'pre-event' messages are written as, or seem to be more appropriate, to pre-season/early season messaging, as they seem unlikely to be steps that one is likely to take during the immediate time before a heat warning (i.e., Workers at Risk Pre-Heat Event).”
“Maybe revisit the word “stress” for at-risk workers after the event. Anglicism?”
“Not scheduled breaks but planned breaks.”
“Concern for certain workers who can hardly demand breaks and especially not in a lying position... Reformulate and target employers and CSST and unions.”
“Workers at risk: are employers informed of the measures to take beforehand?”
“Workers at risk: A distinction between workers at risk in indoor and outdoor environments seems relevant to me. Indeed, the actions to be implemented may be different. In addition, workers are exposed to heat in indoor environments all year round. For reasons of inclusiveness, change the word “workers” to “population of workers” or “workers”. It is much too vague … we suggest applying the instructions dictated by the CNESST in the event of significant heat in the workplace (or something similar). https://www.cnesst.gouv.qc.ca/fr/prevention-securite/identifier-correct-risks/liste-information-prevention/evaluate-level-risk-by-hot-weather. This category should not even be presented here, in my opinion, because we enter the heatstroke dimension due to physical activity.”

Message 12: Medical Consultation

Readability

	Flesch Kincaid Reading Ease			Flesch Kincaid Grade Level			Gunning Fog Score			Smog Index			Automated Readability Index			Words			Complex Words		
**Original**	-	58	-	-	9	-	-	14	-	-	10	-	-	10	-	-	16	-	-	3	-
**Revised 1**	47	28	54	16	12	11	19	16	12	12	10	8	18	12	13	73	19	22	8	6	2
**Revised 2**	66	49	60	6	10	11	8	13	13	7	10	8	5	10	13	27	25	25	4	6	2
**Revised 3**	74	37	79	5	10	6	8	11	9	6	8	6	4	11	7	26	14	15	3	4	1
**Final**	-	-	-	-	-	-	-	-	-	-	-	-	-	-	-	-	-	-	-	-	-

Additional Comments

Respondent Comments
"The likelihood that this would impact a significant population is low. Don't automatically include it in ECCC alerts. It should be an option."
"Seems more educational, awareness raising ongoing not necessary to put in an alert message when other messaging can be more critical. We run the risk of over messaging and people will tune out."
"Column 1 wording suggestion: Certain health conditions and medications can increase your risk for heat illness. Talking with a health care provider before heat occurs is important to know if and how you need to take extra care. Seek advice about modifying your daily activities, medications, or fluid intake during extreme heat. Column 2: "Follow the advice of your healthcare provider." Column 3: Seek advice about modifying your daily activities, medications, or fluid intake during extreme heat."
"I am uncomfortable including this direct/specific medical information within a heat alert from ECCC. Perhaps this could change to something less direct."
"This is better suited to a different form of health messaging, other than alert messaging."
"Include "stock up on medications in case you will not be able to leave your house for a few days" this includes heart and asthma medications."
"We believe that medication use or health conditions do not put people at more risk of exposure to heat but make people more physiologically vulnerable to developing heat-related health effects. In agreement with the premises, it is proposed to add: "Controlling underlying health conditions is essential to protect yourself from the heat."
"I generally agree with the statements, but messages about specific situations might be better when that context is needed. Otherwise, someone might wonder why they are being told to continue taking meds as directed. Was there some reason why this would not be the case?"
"From my experience … this messaging is at a grade 10+ level. I think that some terminology can be adjusted to simplified terminology. For example, modify – change."
"Consider applying consistency in the wording used for health care providers- professionals, and avoid referring to doctors or pharmacists, as these can be deterrents for persons who don't have access to one."
"Pre-heat event/Medical Consultation: Avoid prepositional phrasing in sentences. Also, I think that action could be broken into two steps (check labels, then ask for help). Possible alternative wording: Review medical prescription labels and information to see how the sun and heat affect your medications, therapies, and health conditions. Consult your pharmacist or physician if unsure."

“Particularly, some of the 'pre-event' messages are written as, or seem to be more appropriate, to pre-season/early season messaging, as they seem unlikely to be steps that one is likely to take during the immediate time before a heat warning (i.e., Medical Consultation Pre-Event).”
“There is a lack of post-heat event action items aimed at preparing for the NEXT event. Post-event is where we can nudge people to be better prepared for next time. For example, the many health-related questions we should add, "If you felt unwell during the heat, consult your doctor to discuss how you can reduce your risk for the next event."
“Medical consultation - before the statement seems complex to me. Several sentences are very long.”
“Some expressions (be sure to consult a doctor) could be shorter.”
“Medical consultation - during perhaps separate the two ideas? e.g., If you haven't already done so, consult your pharmacist or doctor to find out how the sun and heat can affect you depending on your medications. In all cases, continue taking your medications as prescribed unless your healthcare professional advises otherwise.”
“Medical consultation: Before the event, Ask a healthcare professional for advice tailored to your situation for dealing with the heat. Before the event: The instruction is completely inappropriate. The most important instruction is to control the underlying disease. Rather, make sure your underlying health conditions are well controlled than providing pharmacological advice.”

Message 13: Information and Resources

Readability

	Flesch Kincaid Reading Ease			Flesch Kincaid Grade Level			Gunning Fog Score			Smog Index			Automated Readability Index			Words			Complex Words		
**Original**	-	-	-	-	-	-	-	-	-	-	-	-	-	-	-	-	-	-	-	-	-
**Revised 1**	-	-	-	-	-	-	-	-	-	-	-	-	-	-	-	-	-	-	-	-	-
**Revised 2**	46	25	40	13	14	15	15	12	17	10	12	12	14	15	16	25	17	29	3	4	4
**Revised 3**	66	40	94	7	10	2	10	7	3	7	7	2	6	9	3	23	24	17	3	4	0
**Final**	66	40	94	7	10	2	10	7	3	7	7	2	6	9	3	23	24	17	3	4	0

Additional Comments

Respondent Comments
“Wording suggestions: Before alert: Remove "and protect from heat" (not necessary). Remove references to the website and the application during the alert since the alerts have already been received. After the alert, replace with "Keep useful contacts at hand...". I would remove "To be ready for the next ..."
“For the message broadcast with the heat alert, I would replace the sentence: "Monitor for current heat alerts by checking the public weather alerts website or the WeatherCAN app." with "Monitor current heat alerts by checking the meteo.gc.ca website or the WeatherCAN app."
“… The public is encouraged to monitor for current alerts and forecasts issued by ECCC directly via the Public Weather Alerts website or o by downloading the WeatherCAN App.”
“Would it be possible to add references to help people find these resources?”
“A bit vague as to what the ‘health and emergency authorities’ that can help you stay cool and safe might be. Services might be a better word than authorities. Less charged and would encompass groups like community outreach (e.g., meals on Wheels).”
“Yukon currently does not have a public health authority. Suggested revision for the ‘released with heat warning’ Monitor current heat alerts via the Public Weather Alerts website or the WeatherCAN app. Follow the recommendations of Environment Canada or your region's public health authority.”
“I think "via the Public Weather Alerts website or the WeatherCAN app" is too vague and prescriptive. ECCC alerts, maybe, which we'd love for them to access directly from the weather office website, but realistically, those alerts are disseminated through a lot more platforms than just that.”
“We propose adding to the second column: “Monitor future heat alerts by consulting the Public Weather Alerts website.” We also suggest adding to the third column: “Monitor heat alerts by visiting the Public Weather Alerts website.” New heat episodes could still occur.”
“Pre-heat event/ Information and Resources: The advice to “Watch for heat warnings and follow the recommendations of Environment Canada or your region’s public health authority.” It is probably better placed here. As well, I don’t think “Be aware of local community resources…” is specific enough to motivate much of a response.”
“Information and resources - post-event is too late for this advice as a standalone.”
“I also feel like Information and Resources - During Event message would be more appropriate in pre-event, or that it might seem strange to suggest the public follow ECCC recommendations in an ECCC alert product. This almost seems like a pre-event message, and the during-event message would be better worded more generally about heeding official sources for information and advice.”
“Particularly, some of the 'pre-event' messages are written as, or seem to be more appropriate, to pre-season/early season messaging, as they seem unlikely to be steps that one is likely to take during the immediate time before a heat warning (i.e., Information and Resources Pre-Event).”

“Pre-Heat event messaging would be beneficial starting at the beginning of the heat season, not just with the expectation of a heat event allowing the population to prepare before the season or with more time to action the suggestions (preparing contact lists, checking with health professionals, familiarizing themselves with local community resources, etc.).”
“Information and resource: The last message differs greatly from the previous ones. We propose to modify it to: ‘keep the contacts and links that were useful to you during the heat event to be prepared for the next one.’”
“Message that may confuse. It doesn't take two messengers. As there is no consultation between the two organizations, it is better to follow the recommendations of only one organization to avoid confusion. Here, we encourage the involvement of the public health authority.”
“Information and resources - before resources are indicated, but not the type of information they could transmit or in what ways this information would be relevant. Some may also want advice on adapting their accommodation or planning a change in their habits. However, the message does not offer any clues in this regard. After: we move away from the search for information.”

Message 14: Air Quality

Readability

	Flesch Kincaid Reading Ease			Flesch Kincaid Grade Level			Gunning Fog Score			Smog Index			Automated Readability Index			Words			Complex Words		
**Original**	-	-	-	-	-	-	-	-	-	-	-	-	-	-	-	-	-	-	-	-	-
**Revised 1**	-	-	-	-	-	-	-	-	-	-	-	-	-	-	-	-	-	-	-	-	-
**Revised 2**	-	-	-	-	-	-	-	-	-	-	-	-	-	-	-	-	-	-	-	-	-
**Revised 3**	67	54	77	9	9	8	10	11	10	8	8	6	10	11	7	19	14	20	2	2	1
**Final**	25	54	77	19	9	8	19	11	10	14	8	6	22	11	7	37	14	20	6	2	1

Additional Comments

Respondent Comments
“Suggested wording: Before alert: replace with: "Check the air quality health index in your area and reduce your exposure..."
“Unlike some of the other messages, this one is critical to have as an automatic alert. More and more we are seeing wildfire smoke and poor air quality with heat.”
“For the message that goes out when the event ends, I would replace the sentence, "If the air quality has improved, open the windows and doors to let in the fresh air during the night, if it is safe." with "If the air quality has improved, if possible, open the windows and doors to let in the fresh air during the night.”
“Thank you for including these! Column 1: Suggestion: Check the AQHI and monitor your symptoms. Column 2: Suggested wording revision to include most sensitive populations: Although air pollution and smoke can harm your health, extreme heat is more dangerous. Whenever possible, keeping cool should always be your priority. Is there any masking advice that can be included in these as well? … Personal protective equipment like respirators (e.g. N95, KN95 or P100) may reduce exposure to wildfire smoke but are most effective when used as part of a multi-layered approach. Other important layers include staying indoors with doors and windows closed as long as temperatures remain comfortable, cleaning indoor air, and minimizing outdoor activities.”
“To be shared only in the event of forest fires. Provide resources to find the Air Quality Health Index. The last message mentions opening the windows afterwards. This gives the impression that they should not be opened otherwise. However, I believe indoor heat is more harmful than air quality.”
“The 'during event' message might end better with "...prioritize keeping cool, preferably indoors."
"When an extreme heat event occurs with wildfire smoke, prioritize keeping cool." I'm unsure If I understand how this is meant to change behaviour. Is it saying heat should be the number one concern, even if it means exposing oneself to wildfire smoke? Not sure if there is any data to support that.”
“Should be included if there is also a SAQS or AQA for the region. For rural settings without AQHI, the statement about AQHI doesn't apply. Suggest making this regional/ only applicable to locations with AQHI forecasting.”
“I understand the value of including AQ but wonder whether this would be more effective to deploy only in the event of a co-occurring heatwave and wildfire smoke so that the message's efficacy does not dilute. Also, a website link to AQHI should be included.”
"When an extreme heat event occurs with wildfire smoke, prioritize keeping cool." Is the intention for people to keep windows closed or open them? This statement implies that keeping cool is the priority, as with open windows, but this could harm those with cardiovascular or respiratory conditions. Proposed edit: "When there is

an extreme heat event occurring with wildfire smoke, prioritize keeping cool while limiting your exposure to smoke."
"From our point of view, the wording of the pre-event message suggests an immediate presence of forest fires. Perhaps state: "If wildfires are active in your area, plan to check the CAS…" Furthermore, we think the CAS should not be considered when a sector is on heat alert. Otherwise, it is difficult to consider which messages become a priority. Protection against heat must, of course, take precedence."
"Generally ok, but during the event, there might need to be some message about monitoring sx for people that are sensitive to smoke."
"Event ends wording applied during the event, AQ improving during the heat is just as likely as after the heat. and from my readings, it seems like heat is the most concerning for most people, but not for all medical conditions (so wouldn't checking with medical professionals be advised in the prep stage?)."
"Heat and wildfire smoke is unfortunately common in our summer months, with often overlapping events. Focused messaging with these events in mind would be well received. Additional consideration of heat events during active emergency evacuations would be appropriate, even if a link to appropriate provincial sites is provided for more information during these events. Messaging for consideration: Extreme Heat and Special Air Quality Statements (SAQS)/ or Air Quality Advisories (AQA): (1) Although air pollution and smoke can be harmful to your health, extreme heat is more dangerous. Whenever possible, keeping cool should always be your priority. (2) Run an air conditioner, if available, and recirculate air within a space. (3) If available, portable air filters can improve air quality. (4) Reduce heat entering a space by keeping blinds, curtains, and doors closed during the day. (5) Limit heat-generating appliances (stoves, ovens, televisions, etc.), as possible. (6) If air quality has improved, open windows and doors to move cool air into the nighttime space. (7) If you or those in your care are exposed to wildfire smoke, consider taking extra precautions to reduce your exposure, such as reducing or rescheduling outdoor physical activities. The Air Quality Health Index value in your region can be used to gauge appropriate actions based on AQHI level and whether you are at risk."
"I'm certain that some compounding events are not captured here or might cause some conflict in the recommendations, but these may need to be handled on a case-by-case basis. I can tell that effort has gone into making them more widely applicable."
"We have access to other cooling options like lakes that would be a good cooling option for some groups - many of our heat events might also be poor air quality events (due to wildfire smoke), and this messaging does not address air quality at all. The messaging does not account for overcrowded housing situations."
"The messages do not specifically highlight compounding events."
"If forest fire smoke, add aggravation factor if poor air quality."

Message 15: Nighttime

Readability

	Flesch Kincaid Reading Ease			Flesch Kincaid Grade Level			Gunning Fog Score			Smog Index			Automated Readability Index			Words			Complex Words		
**Original**	-	-	-	-	-	-	-	-	-	-	-	-	-	-	-	-	-	-	-	-	-
**Revised 1**	-	-	-	-	-	-	-	-	-	-	-	-	-	-	-	-	-	-	-	-	-
**Revised 2**	-	-	-	-	-	-	-	-	-	-	-	-	-	-	-	-	-	-	-	-	-
**Revised 3**	109	89	-	0	3	-	2	4	-	2	2	-	-3	3	-	11	28	-	0	0	-
**Final**	-	-	-	-	-	-	-	-	-	-	-	-	-	-	-	-	-	-	-	-	-

Additional Comments

**Respondent Comments**
“Suggestions: before the alert, change to "Plan to sleep in a cool space." During the alert, reverse the sentences, since the shower comes before sleeping: "Take a cool shower before bed and wear light and loose clothing. Sleep in the coolest part of your living space and open the windows if it's safe."
“I feel like not everyone will have access to a cool shower, etc. We should reserve text within an active alert to the highest risk statements so the text doesn't get too long and none of it is read.”
“But for reference, add ‘at night’ to both suggested Round 2 messages.”
“For the message broadcast with the heat alert, I would replace the phrase: "Sleep in the coolest part of your living space and open the windows if it is safe to do so." with "Sleep in the coolest part of your living space and open the windows if possible."
“Awareness of the impact of nighttime temperatures is important. Consider integrating the following messages in the pre-heat event messaging. Extra care and awareness are needed both indoors and outdoors. Elevated indoor temperatures, especially at night, can be hazardous to health. High indoor nighttime temperatures can be hazardous to your health because it prevents your body from fully recovering from the heat.”
“What if the accommodation does not offer this possibility?”
“It seems to me that a message should be sent for the end of the alert, especially if the indoor temperatures remain warm a few days later?”
“Again, might replace the ‘N/A’ with ‘Continue taking precautions until the temperature in your home falls to a comfortable level.’"
"‘Plan to ensure’ could be just ‘plan to’ or ‘ensure’ ‘if safe and outdoor temperatures lower than indoors.’”
“Not necessarily appropriate for those in all socio-economic conditions.”
“Watch stage wording is clunky...plan or ensure, not both.”
“I'm not sure the early warning is needed, but the heat alert is useful.”
“Add ‘if nighttime temperatures do not drop below 22°C (or below a certain level), your body's ability to adapt to the chronic exposure to heat begins to suffer, leading to adverse health outcomes’.”
“Use a light sheet to sleep on and remove the duvet from your bed. Plan to move to a cool space to spend the night if possible. Don’t hesitate to open your windows if it’s colder outside than inside. Furthermore, we do not consider it useful to issue advice regarding wearing loose, light clothing in bed.”
“I wonder about ‘cool shower’ and how this will be interpreted. Could result in peripheral vasoconstriction and not promote heat loss?”
“Lack of pragmatism, be careful: windows open at night accessible to intruders, especially for the elderly and women = add a note to ensure your security, especially at night.”

"Maybe this is where the open windows and cross-breeze comment works best?"
"It would be good to emphasize the need to Stay Cool at Night: High indoor nighttime temperatures can be hazardous to your health because they prevent your body from fully recovering from the heat. For example, whenever it is safe and possible, open windows and doors in the evening to help cool your residence. Options: (1) Sleep in the coolest part of your residence (e.g., designated 'cool room'), (2) Sleep outside if it is safe and appropriate to do so, (3) Mist bed sheets with water, (4) Wear light and loose-fitting clothes to bed, (5) Ensure at least one room in your residence remains cool to provide a designated place to escape the heat and recover. This is especially important for sleep. (6) The National Collaborating Center for Environmental Health has a guide on completing in-person or remote health checks for your heat buddy: Health Checks During Extreme Heat Events."
"Add notions of duration: if more than 24 hours, if overnight stay without respite of less than 25°, if more than three days, if more than a week."
"What about measures for nighttime when people don't have air conditioning systems?"
"Take a cool shower or bath before bedtime and, if possible, during the day."
"Cooling centers: what to do at night?"

General Comments

Respondent Comments
"The messages would benefit, at least in French, from being even simpler for the average literacy level of Canadians. Sentences should be as short as possible and situated in the action. If possible, have it reviewed by a French-speaking communicator."
"Has consideration been given to include some messaging for pets? For Livestock? Expand the water-hydration statements throughout. For the events ending, consider adding 2-5 days."
"Overall, the pattern should remain consistent: Prepare - Take Action – Continue."
"I've recommended including most, if not all, of the proposed actions in the alerts. If only a subset is to be included, perhaps based partly on the mean rankings, then providing a direct hyperlink and contact point to the complete set of recommended actions would be helpful."
"Reading through all the statements on the previous page. This statement here: Watch for the early signs of heat illness in yourself and others. It says everything that needs to be said. Most statements say the same thing; they just add more detail. You could still decrease the number of messages by comparing that statement with the others and removing the statements saying the same thing."
"I found some of the responses and actions a bit vague. For example, "Take action to protect yourself and others - extreme heat can affect everyone's health. Determining if you or your family are at greater risk of heat illness" doesn't give the individual much to go off. Especially in the ranking exercise, I felt the presented statements lacked some context. For example, the statements relevant to workers are not explicitly specified as such (I know it says "when working in the heat," but this is still somewhat ambiguous)."
"For question 43, "Do the proposed messages include the necessary conditional disclaimers needed?" to be honest, I do not know, but I could not change the selection to leave it blank. It is better to consult with MSC's tech team … to release different messages at different times. Even if it is doable, with so many different heat-health messages, to reduce the workload and confusion, it is better to have pre-selected messages at different time points, which need to be decided by warning system or forecasters."
"Thanks for this. Continue to think about rural settings when developing this with limited forecasting & infrastructure for staying cool."
"I think having direct links embedded where people can find their local cooling centres would be very valuable. Also, it would be helpful to have these messages in graphic form and multilingually."
"My overall concern is about message length. There are too many statements, and the messages are very long. Work can be done to edit them further. Research tells us warning messages need to be succinct and contain what is most important because people will stop reading."
"Folks in shelters, experiencing homelessness, living with low income will struggle to buy fans, find shade, or drink cold water. Maybe a message for housing services or targeted resources?"
"It is beautiful!"
"The ranking question was difficult to answer. Some of the statements could be put together to remove redundancies while keeping the important messages."
"We find the messages better formulated in this new version, more concise and direct. THANK YOU! We propose to discriminate between the concepts of vulnerability and heat risk. When it is proposed to refer to a thermometer or a given temperature, it seems important to us to identify benchmarks to guide the population in their decision-making. We suggest using the same terms from one message to another. For example, the entourage is sometimes replaced by family, friends and neighbours, which can be confusing. We propose clearly defining certain terms and concepts used (e.g., a symptom of exposure to heat versus effects of an illness attributable to heat). It is proposed to limit the number of messages; those presented in a post-event situation generally seem less essential to us. The majority of recommendations articulated do not appear to be based on evidence. We should take the time to highlight those based on effective communication interventions. A group of typical users could be formed to test the reception of certain messages. There is a potential for confusion when other health authorities do not give the same recommendations (for example, the CNESST concerning workers)."

"There might be an opportunity to combine the first message and the check-in message - the first message is obligatory, but it's more about assessing self/family's risk rather than taking some risk-reducing action, even though it starts with "Take action..." What seems more action-oriented is the check-in concept for others (and also oneself). Whatever the messages, grouping might help - in looking at the string of messages in the examples given; it's a lot of text; less might be more, and grouping messages might help signpost for the reader."
"This might be more important regarding the chosen medium/format and selective reach of the information rather than the content alone. Just because roughly 90 percent of Canadian households own at least one cellular device does not mean they all directly receive heat warnings and attendant protective health messages. Understanding the less formal flow of information from warnings to populations considered particularly vulnerable might be a worthwhile exercise. More generally, in times of limited resources (time, money, etc.) and where success is measured in terms of risk outcomes, it might be prudent to do the opposite (Warnings4All) and concentrate explicitly on populations and activities (times, places, situations) where vulnerability and exposure are heightened and where the capacity to respond as recommended is limited. This would address the second question more than the fairly generic messages (understandably so because they must relate to all) in the table."
"I feel that some of the pre-heat event messaging would be more valuable pre-heat season and before an event. With only 48 hours before the heat warning, some suggested activities and information may not be implemented and may require more time for the public/user to implement."
"There are instances where the messages could be made even simpler without deterring from the messaging itself."
"It is a tight space to communicate key messages without getting wordy. An additional consideration to providing a spectrum of options based on geographical location (urban vs rural) and across the economic spectrum is to ensure all populations can see themselves in the messaging."
"I think there is always more room for improvement here; I think more specific details would be beneficial, considering First Nation community needs and specific populations; however, duplicating different types of messaging with the same details written in different ways could be redundant. As written, the messaging is equitable and covers all socio-economic backgrounds."
"This assumes people have a home, car, phone. In general, yes, but how do we target and mitigate the risk of those in transient housing? Will these messages reach Indigenous individuals?"
"Outside my bailiwick, but I would assess the readability of the language at somewhere between Grade 8-10 level."
"I think these are mostly okay at a grade 6 level/minimal jargon, but I do wonder about some of the word choices like "elevated," "excess," and "modify." It feels like there are simpler words that could be used for these."
"The messages have been designed based on a content analysis (in NVivo) of the websites of HC and all other federal agencies, supplemented by articles related to heat exposure and cooling interventions in four academic databases. Although the methodology sounds inclusive, it amplifies any biases in these systems. In other words, many prevalent concerns around representation will be passed down in the future through methodology. The trend continues, and people become accustomed to being left out of the equation."
"Important! We are hearing clearly from our stakeholders that pre-heat event messaging is vital to help them be prepared for the events."
"Question 7: Some of the messages would not work in the ECCC weather system as written/proposed; some messages are repetitive and would likely be better combined (i.e., Medical Consultation Post-Season and Hydration Post-Season). There would also need to be additional coordination/consideration about when to choose which messages; there are limits/considerations to the length of warnings, especially in the context of some of the additional/adaptive methods to communicating those alerts (HelloWeather phone line, Weatheradio, etc). Forecasters might find it hard to know which messages to use on the fly while issuing alerts for heat and other weather events."
"Is the intent to package the messages in the warning and keep the order in the left column? If so, then from the top down, I see an intent to define and draw attention to the hazard (first two rows, parts of the second-last row) and then describe various actions/behaviours to reduce risk. I wonder whether the order of the rows/actions reflects the importance of the risk outcome. It might be better to emphasize the last two rows (response to acute health situations) first as they may raise concern/attention around the implications of the threat introduced in the first two rows. Then perhaps review, systematically prioritize, and reorder the remaining

recommended actions based on levels of exposure/vulnerability (most people affected and most vulnerable situations/activities/populations) and the target of the action/behaviour (individual, household, extended family/friend network, co-workers). For example, "Information and Resources" is likely much less important than other items; occupational health issues might be better directed to businesses and enforcement personnel."
"Most messages are fine inappropriateness, but they do not consider various types of vulnerabilities in the population. Suppose the intent is to inform employers and organizations of accountability and transparency. In that case, these messages are fine, but if these are designed with the public in mind, there are gaps in overlooked demographics. When certain people don't see themselves in the messaging, they lose interest, and their trust in the system will gradually erode. Also, if "many provincial/territorial and regional health authorities, as well as other public health stakeholders, have developed their own messaging, guidance and resource documents separate from Health Canada," then I think the same messages could be used here as well, rather than creating another set/ layer of messaging."
"I find that I was looking for certain messages that only appeared in separate subsections, for example, cooling centre information under the "info and resources" messages (not in the post heat wave section); "seek medical care" under the medical consultation title; signs and symptoms under hydration post heat event. I wonder if there is a way to ensure both would get published side by side."
"First, congratulations on assembling a great table/list. This is not a simple or easy endeavour, and a single 'best' solution will never exist for a general public warning. Most, if not all, of these messages seem important. The question is if the messages—whether left intact, edited, reordered, or removed—independently or collectively have any efficacy at motivating the risk-reducing behaviours of recipients and those with which they might directly or indirectly (e.g., modelling behaviour) influence (family, friends, co-workers, neighbours). The messages more specific to an individual's situation and circumstances are likely more relevant and actionable. Still, practical limits on message length mean introducing more generalized language (relevant to most of the population). This becomes a tradeoff between reaching the most people at some modest level of risk and connecting meaningfully with those at much greater risk due to one or more of the many predisposing exposure or vulnerability factors listed later in this survey instrument. Suppose a significant 'message tailoring' level can effectively sway behavioural intentions/behaviours. In that case, it makes sense to examine (costs relative to potential benefits) an alter/warning system interface that forces a top-down categorical or, ideally, self-determined assessment for the presence of key risk determinants (those factors listed later on) with commensurate tailored recommended action messaging. Thinking through the definition of an event, not in meteorological threshold terms, but rather in 'desired precautionary action and risk outcome' terms might be worthwhile. In my experience/research with other hazards, a risk 'event' begins whenever evidence suggests that an action or intervention is helpful or when looking retrospectively, an action/response was taken. Many actions or responses may be necessary well in advance (take longer, need more lead time, prerequisites for subsequent short-term responses). After a period of excessive and prolonged heat and humidity, framing messages within what looks like a forced 48-hour window before and a 24-hour window following a heat warning may limit the success of public response in terms of risk outcomes. Related to this, but keeping with the temporal partitioning, there may be a fourth distinct timeframe (pre-season and ahead of the first event of the season or particularly severe/unusual events) that might deserve explicit recognition, attention, and specific messaging. For example, actions that require greater lead time and could increase the response capacity of individuals/households, like testing/servicing/purchasing/installing air conditioners, fans, outdoor/indoor shading options (plantings, awnings, window dressings, etc.), subgrade living spaces (basements), community pool or recreation centre passes/program registrations, etc. Current sub-seasonal (2 weeks to 3 months) predictions are sufficiently valid to trigger such advanced communications potentially."
"The document is vague, does not particularly target vulnerable customers, mixes the types of heat break, gives too many messages, some of which have no evidence of being effective to the detriment of those we know to be, mixes the responsibilities of the competent authorities, and could strongly contribute to the confusion of the messages. This document should not be published."
"Sentences are sometimes very long."
"Yes, but without the references, it is difficult to assess whether the recommendations are based on evidence or at least what data. Perhaps add an appendix that explains this data and how it supports the statements?"

“Temporal sequences are not well established for certain messages: communicating only 'during' is less effective than 'before' AND 'during.'”
“Add pictogram, illustrations (tested).”
“Messages are action-oriented. Good indication of the times to carry out the actions.”
